# Omega-3 fatty acid desaturase gene family from two ω-3 sources, *Salvia hispanica* and *Perilla frutescens*: Cloning, characterization and expression

**DOI:** 10.1371/journal.pone.0191432

**Published:** 2018-01-19

**Authors:** Yufei Xue, Baojun Chen, Aung Naing Win, Chun Fu, Jianping Lian, Xue Liu, Rui Wang, Xingcui Zhang, Yourong Chai

**Affiliations:** 1 College of Agronomy and Biotechnology, Southwest University, Chongqing, China; 2 Academy of Agricultural Sciences, Southwest University, Chongqing, China; 3 Chongqing Engineering Research Center for Rapeseed, Southwest University, Chongqing, China; 4 Chongqing Key Laboratory of Crop Quality Improvement, Southwest University, Chongqing, China; 5 Engineering Research Center of South Upland Agriculture of Ministry of Education, Southwest University, Chongqing, China; Huazhong University of Science and Technology, CHINA

## Abstract

Omega-3 fatty acid desaturase (ω-3 FAD, D15D) is a key enzyme for α-linolenic acid (ALA) biosynthesis. Both chia (*Salvia hispanica*) and perilla (*Perilla frutescens*) contain high levels of ALA in seeds. In this study, the *ω-3 FAD* gene family was systematically and comparatively cloned from chia and perilla. Perilla FAD3, FAD7, FAD8 and chia FAD7 are encoded by single-copy (but heterozygous) genes, while chia FAD3 is encoded by 2 distinct genes. Only 1 chia *FAD8* sequence was isolated. In these genes, there are 1 to 6 transcription start sites, 1 to 8 poly(A) tailing sites, and 7 introns. The 5’UTRs of *PfFAD8a*/*b* contain 1 to 2 purine-stretches and 2 pyrimidine-stretches. An alternative splice variant of *ShFAD7a*/*b* comprises a 5’UTR intron. Their encoded proteins harbor an FA_desaturase conserved domain together with 4 trans-membrane helices and 3 histidine boxes. Phylogenetic analysis validated their identity of dicot microsomal or plastidial ω-3 FAD proteins, and revealed some important evolutionary features of plant *ω-3 FAD* genes such as convergent evolution across different phylums, single-copy status in algae, and duplication events in certain taxa. The qRT-PCR assay showed that the *ω-3 FAD* genes of two species were expressed at different levels in various organs, and they also responded to multiple stress treatments. The functionality of the ShFAD3 and PfFAD3 enzymes was confirmed by yeast expression. The systemic molecular and functional features of the *ω-3 FAD* gene family from chia and perilla revealed in this study will facilitate their use in future studies on genetic improvement of ALA traits in oilseed crops.

## Introduction

In the family Lamiaceae, chia (*Salvia hispanica*, 2n = 12) and perilla (*Perilla frutescens*, 2n = 40), which are annual herbaceous plants, are 2 rich sources of ω-3 polyunsaturated fatty acids (PUFAs). Chia is native to Mexico and parts of South America, and perilla originated in Asia [1–4]. Chia and perilla seeds contain 25~40% oil, and ɑ-linolenic acid (ALA, 18:3^Δ9,12,15^) proportion in their seed oil is the highest among crop sources (about 60~71%) [5–7]. ALA is known as an essential fatty acid (FA) for the human daily diet because ALA cannot be synthesized in the human body due to the absence of the *ω-3 FAD* gene [8]. ALA has a wide variety of health benefits as it is a necessary substrate for the biosynthesis of very-long-chain ω-3 PUFAs, eicosapentaenoic acid (C20:5^Δ5,8,11,14,17^, EPA) and docosahexaenoic acid (C22:6^Δ4,7,10,13,16,19^, DHA). It has been reported that EPA and DHA regulate body development and growth, promote brain development, reduce blood pressure, inhibit senescence, and also have beneficial effects on neurological, cardiovascular and cerebrovascular diseases [9]. Moreover, ALA functions as a crucial component of membrane lipids and triacylglycerol seed storage lipids in higher plants [10]. Additionally, ALA is also a precursor of FA-derived signal molecules, e.g., jasmonic acid (JA), that play important roles in plant development and stress responses [11]. With respect to the ALA biosynthesis pathway, a small portion of pivotal genes has been described in perilla [12–15], but genetic and molecular studies are relatively rare for chia [16]. Hence, systemic cloning of the *ω-3 FAD* gene family of both chia and perilla was performed in this study, which is the first report to provide the full-length cDNA and genomic DNA (gDNA) sequences of chia *ω-3 FAD* genes, as well as the full-length gDNA sequences of perilla *ω-3 FAD* genes.

In plants, ω-3 fatty acid desaturases (FAD3, FAD7 and FAD8) have been documented to be responsible for producing ALA from LA in the endoplasmic reticulum (ER, FAD3) and plastids (FAD7 and FAD8) by introducing a third double bond at the Δ15/ω-3 carbon position of LA [10]. The ALA biosynthesis in seeds is mainly catalysed by ER-type FAD3s, while in plastids by FAD7 and FAD8 [8]. Omega-3 FADs were encoded by nuclear genes [17]. Since the initial discovery of the model plant *Arabidopsis ω-3 FAD* genes, their orthologous genes have been cloned and characterized from diverse other plant species, such as flax (*Linum usitatissimum*) [18,19], soybean (*Glycine max*) [17,20,21], cotton (*Gossypium hirsutum*) [22], sunflower (*Helianthus annuus*) [23,24], rapeseed (*Brassica napus*) [25,26], safflower (*Carthamus tinctorius*) [27], and purslane (*Portulaca oleracea*) [28]. *Arabidopsis thaliana* contains only one member for each *ω-3 FAD* gene [29–31], and corresponding orthologue might harbor several copies in some other plants. Three genes encoding FAD3 were isolated and identified from 4 flax cultivars with varying ALA contents [32]. Olive contains 2 *FAD3* and 2 *FAD7* genes, and the expression levels and lipid contents in different tissues for these genes were determined [33]. Six *ω-3 FAD* genes (*OsFAD3*/*7*/*8* and *GmFAD3-1*/*-2*/*-3*) were isolated from rice and soybean, and their subcellular location, and their effects on ALA content in rice seeds, were evaluated using overexpression under the control of an endosperm-specific expression promoter, GluC, and a constitutive expression promoter, Ubi-1 [8]. Additionally, the ω-3 FAD proteins contain 3 histidine boxes (motifs) that are essential for maintaining FAD catalytic activity [34], and strong transmembrane domains that are typical characteristics of membrane-bound FADs. In general, the FAD3s possess a C-terminal ER-retrieval motif, e.g., KSKIN in AtFAD3 [35], while FAD7/8 proteins consist of an N-terminal chloroplast transit peptide leading to their subcellular location.

It has been reported that the plant *ω-3 FAD* genes play important roles in response to a variety of environment factors, including temperature [36–39], salt [40], drought [41,42], wounds [28,43–45], light [46,47], hormones [48,49], and pathogens [50]. In the leaves of birch seedlings (*Betula pendula*), the *BpFAD7* transcript was down-regulated by low temperature, whereas the expression levels of *BpFAD3* and *BpFAD8* were up-regulated and the ALA content in glycerolipids also increased [51]. Antisense-mediated depletion of tomato (*Lycopersicon esculentum*) *LeFAD3* increased the saturation degree of fatty acids and alleviated high temperature stress [52], whereas overexpression of tomato *LeFAD3* enhanced the tolerance of early seedlings to salinity stress [53]. Cells and plants of transgenic tobacco (*Nicotiana tabacum*) overexpressing *NtFAD3* or *NtFAD8* showed increased tolerance to drought and osmotic stress [54]. *Descurainia sophia DsFAD3*/*7*/*8* transcripts were significantly induced by wound stress [45]. Rapeseed (*Brassica napus*) *BnFAD3* expression was induced by abscisic acid (ABA) [55]. In soybean, JA accumulation in *GmFAD3*-silenced plants increased, which resulted in increasing the susceptibility to bean pod mottle virus (BPMV) [56].

*FAD3* cDNA was isolated from the developing seeds of perilla, and its mRNA accumulation manner was seed-specific [12]. Recently, the expression profiles of *ω-3 FAD* genes in developing seeds of perilla by transcriptome analysis have been investigated, which showed that *FAD3* and *FAD7*/*8* were determined to be pivotal genes for ALA synthesis in seeds and leaves, respectively [14]. Additionally, the subcellular location and catalytic activity of perilla *FAD3* and *FAD7-1*/*-2* were confirmed [13]. For chia, transcriptome profiles and expression analysis of Δ15/ω-3 desaturase genes in 5 different stages of developing seeds were reported [4]. Unfortunately, systemic cloning and characterization of chia *ω-3 FAD* genes as well as comparative study on the evolutionary relationship, exon/intron patterns, stress responses, and FAD3 catalytic activity between perilla and chia *ω-3 FAD* genes have not so far been conducted. The aim of this paper is to systematically clone and identify full-length sequences of the *ω-3 FAD* gene family from chia and perilla. This study can provide an important reference for dissecting the molecular mechanisms of their high ALA traits, and enrich our knowledge of the crucial roles of *ω-3 FAD* genes in response to abiotic/biotic stresses and plant hormone treatments.

## Materials and methods

### Plant materials, treatments, and nucleic acid extraction

Chia (commercial variety) and perilla (C2 cultivar) were grown in a standard experimental field of College of Agronomy and Biotechnology, Southwest University, China. For each species, roots (Ro), stems (St), leaves (Le), buds (Bu), flowers (Fl), mid-stage seeds (MS, approximately 20 days after flowering), and late-stage seeds (LS, approximately 30 days after flowering) were sampled, and early-stage seeds (ES, approximately 10 days after flowering) were also collected for chia.

For various treatments, chia and perilla seeds were grown in damp soil in plastic pots (20 seeds per pot). These pots were placed in a climatic chamber (30°C and 56% of relative humidity) with 16/8 h of light/darkness. The 5-week-old seedling leaves of chia and perilla were subjected to various stresses. Salt and drought stresses were imitated using 300 mM NaCl and 10% PEG6000 solutions (plants were irrigated), respectively. The seedlings were sprayed with 100 μM ABA, 1 mM SA, and 100 μM MeJA solutions. Cold and heat treatments were carried out in 4°C and 38°C (chia) or 42°C (perilla) chambers, respectively. Wound stress was performed as described in previous report [57]. For each stress, seedling leaves were collected at 0, 0.5, 3, 9, 24, and 48 h after treatment. All collected samples were immediately frozen in liquid nitrogen and stored at -80°C.

Total DNA was extracted from the leaves of 2 species using the CTAB method [58]. Total RNA was extracted from differential organs or seedling leaves of 2 species subjected to various stresses using the RNAprep Pure Plant Kit (Tiangen, China). The quality and concentration of genomic DNA and total RNA were detected by agarose gel electrophoresis and spectrophotometer analysis with a Nanodrop 2000 (Thermo Fisher Scientific, USA).

### Cloning of full-length sequences of *ω-3 FAD* gene family from 2 species

For both chia and perilla, 1 μg of an equally proportioned (w/w) mixture of total RNA from various organs was employed to generate first-strand total cDNA of 5’- and 3’-RACE, respectively, using the SMARTer^™^ RACE Amplification Kit (Clontech, Takara Dalian, China). Based on multi-alignment of the *ω-3 FAD* cDNAs from perilla, flax, olive and other plants, four gene-specific primers were designed to correspond to the conserved sites (S1 Table). Sense primers FPD153-1 and FPD153-2 were paired with the kit universal primers UPM and NUP, respectively, for primary and nested amplifications of 3’-RACE. With respect to primary and nested amplifications of 5’-RACE, the kit universal primers UPM and NUP were paired with antisense primers RPD155-1 and RPD155-2, respectively. In the primary amplification, 0.2 μL of first-strand total cDNA of 5’- or 3’-RACE was used as a template, whereas 0.1 μL of 5’- or 3’-RACE primary amplification product was employed as a template for the nested amplification. All 4 PCR programs adopted the following cycling parameters: 94°C for 2 min; 30 cycles of 94°C for 1 min, 58°C for 1 min, 72°C for 1 min; and 72°C for 10 min. The aforementioned PCR fragments were gel-recovered and cloned into a pGEM-T easy vector (Promega, USA) and sequenced; their identities were confirmed by NCBI BLASTn.

Based on the sequence alignment of *Arabidopsis* and perilla *ω-3 FAD* mRNAs from NCBI GenBank, as well as 5’- and 3’-RACE cDNAs of the *ω-3 FAD* genes from 2 species obtained in this study, allele/member-specific primers of the full-length *ω-3 FAD* genes in chia and perilla were designed (S1 Table). Next, 0.2 μL total cDNA of mixed organs from chia or perilla was used as a template for amplifying full-length cDNA sequences, and the corresponding genomic sequences were also isolated using 0.5 μg total genomic DNA as a template from the leaves. Primer pairs FPfFAD3+RPfFAD3a and FPfFAD3+RPfFAD3b were used to isolate perilla *FAD3* (*PfFAD3*) alleles, FPfFAD7+RPfFAD7 for perilla *FAD7* (*PfFAD7*) and FPfFAD8+RPfFAD8 for perilla *FAD8* (*PfFAD8*). Primer pairs FShFAD3-1+RShFAD3-1 and FShFAD3-2+RShFAD3-2 were used to isolate 2 members of chia *FAD3* family (*ShFAD3-1* and *ShFAD3-2*), FShFAD7a+RPfFAD7a and FShFAD7b+RShFAD7b were used for 2 alleles of chia *FAD7* (*ShFAD7a* and *ShFAD7b*), and FShFAD8+RShFAD8 was used for chia *FAD8* (*ShFAD8*). The thermal cycling parameters of these PCR reactions were as follows: 94°C for 2 min; 35 cycles of 94°C for 1 min, 60°C for 1 min, 72°C for 3 min; and 72°C for 10 min. Gel recovery, TA cloning, and sequencing of amplified genes were performed following general procedures.

### Bioinformatics analysis

Vector NTI v11.5.1 and DNAStar version 7.1.0 were used to perform sequence assembly and alignment, ORF search and translation, parameter calculation, and other bioinformatics analyses. The SMART (http://smart.embl-heidelberg.de/) and Pfam (http://pfam.xfam.org/) databases, Expasy (http://www.expasy.org), CBS (http://www.cbs.dtu.dk/services/), TOPCONS (http://topcons.net/) [59] and GSDS2.0 (http://gsds.cbi.pku.edu.cn/) [60] online websites were used to perform conserved domain (CD) detection, structural predictions of the genes or proteins, and BLAST analyses. Multi-alignment of plant ω-3 FAD proteins was carried out with the MAFFT7 program [61], and a phylogenetic tree was constructed with the Bio Neighbor-Joining (BioNJ) method in SeaView 4.0 [62]. The reliability was examined by bootstrap analysis with 1,000 replicates.

### Quantitative RT-PCR analysis

First-strand total cDNA was generated with 1 μg of each organ or seedling leaves of each stress treatment using the PrimeScript Reagent Kit with gDNA Eraser (Takara Dalian, China). Based on full-length cDNAs of chia and perilla *ω-3 FAD* genes, the corresponding primer pairs in S1 Table were designed for fluorescence real-time quantitative RT-PCR (qRT-PCR) detection of expression profiles of *PfFAD3*, *PfFAD7*, *PfFAD8*, *ShFAD3-1*, *ShFAD3-2*, *ShFAD7* and *ShFAD8*. First, the specificity of the qRT-PCR primers was validated using agarose gel electrophoresis of their PCR products. Then, qRT-PCR was performed with a FastStart Universal SYBR Green Master (Roche, Germany) in a total reaction volume of 10 μL, which comprised 5 μL of 2×SYBER Mix, 0.5 μL of each primer (10 μM) and 2.5 μL of cDNA. Chia and perilla *25SrRNA* were used as reference genes for qRT-PCR with primer pair F25SRT+R25SRT (S1 Table), and these primers were designed according to conserved regions of *25SrRNA* across the plant kingdom. The reactions were performed on CFX96 Real-time PCR system (Bio-Rad, USA) according to the manufacturer’s protocol, and a melting curve analysis was conducted to test whether additional qRT-PCR products were present. The qRT-PCR experiments were performed with 3 biological replicates. All the data were analyzed by using CFX Manager 3.1 (Bio-Rad, USA) with the 2^-ΔΔCT^ method [63].

### Yeast expression and fatty acid analysis

The ORFs of *ShFAD3-1*, *ShFAD3-2*, and *PfFAD3a*/*b* were amplified using primer pairs FShFAD3-1Y+RShFAD3-1Y, FShFAD3-2Y+RShFAD3-2Y, and FPfFAD3Y+RPfFAD3Y, respectively (S1 Table). Each of the 4 ORFs was cloned into the pGEM-T easy vector (Promega) and validated by sequencing. Then, these 4 coding regions containing *Bam*HI and *Xba*I sites were individually inserted into pYES2.0 (Invitrogen, USA) via double digestion and were confirmed by sequencing. The empty vector pYES2.0, and the recombinant plasmids pYES2-ShFAD3-1Y, pYES2-ShFAD3-2Y, pYES2-PfFAD3aY and pYES2-PfFAD3bY were transformed into *Saccharomyces cerevisiae* strain INVScl as described by the pYES2.0 Kit User Manual. Yeast cells were grown to logarithmic phase at 30°C in SC-Ura containing 2% (w/v) raffinose and 0.1% NP-40 using 0.5 mM LA as a feeding substrate. After adding 2% (w/v) galactose, the yeast cells were inducibly expressed and then incubated at 20°C for 72 h. Finally, the yeast cells were collected by centrifugation, washed with sterilized water more than 3 times, and freeze-dried on a ScanVac-Coolsafe 110–4 (Denmark) for 12 h. Separation and gas chromatography (GC) analysis of FA compositions of yeast cell samples were carried out as described in a previous report [64]. Each experiment was carried out in 3 biological replicates.

For chia and perilla *FAD7*/*8* genes, N-terminal chloroplast transit peptides and stop codons of their coding regions both were deleted, and then were fused to rapeseed ferredoxin *BnFD2* gene in N-terminal (its N-terminal signal peptide was deleted; NCBI accession No. XM_013894075.1) using T2A linker peptide [65] (S1 Table). Further, PfFAD7::T2A::BnFD2, PfFAD8::T2A::BnFD2A, ShFAD7::T2A::BnFD2, and ShFAD8::T2A::BnFD2 were individually inserted into the P_GAL1_-T_CAC1_ position of pYES2.0 vector by double digestion (S1 Table). Accordingly, 4 recombinant plasmids pYES2-PfFAD7-T2A-BnFD2, pYES2-PfFAD8-T2A-BnFD2, pYES2-ShFAD7-T2A-BnFD2, and pYES2-ShFAD8-T2A-BnFD2 were generated. As described in above procedures, yeast transformation, inducible expression, and FA GC analysis were performed.

### GenBank accession numbers

KX610645 (*ShFAD3-1* mRNA), KX610646 (*ShFAD3-2* mRNA), KX610647 (*ShFAD7a* mRNA), KX610648 (*ShFAD7b* mRNA), KX610649 (*ShFAD8* mRNA), KX610652 (*ShFAD3-1* gene), KX610653 (*ShFAD3-2* gene), KX610654 (*ShFAD7a* gene), KX610655 (*ShFAD7b* gene), KX610656 (*ShFAD8* gene), KX880387 (*PfFAD3a* gene), KX880388 (*PfFAD3a* mRNA), KX880389 (*PfFAD3b* mRNA), KX880390 (*PfFAD7a* gene), KX880391 (*PfFAD7a* mRNA), KX880392 (*PfFAD7b* mRNA), KX880393 (*PfFAD8a* gene), KX880394 (*PfFAD8a* mRNA) and KX880395 (*PfFAD8b* mRNA).

## Results

### Cloning of full-length *ω-3 FAD* gene sequences from chia and perilla

**Fig 1:**
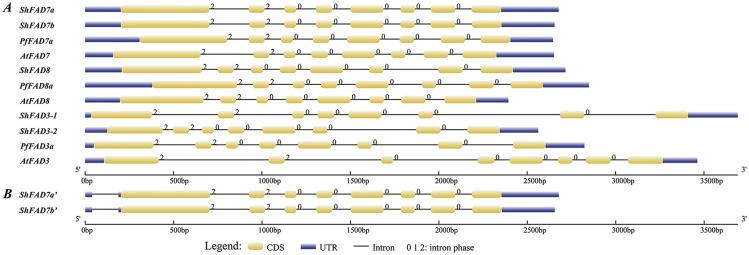
Gene structures of *ω-3 FAD* genes from chia, perilla and *Arabidopsis*. Typical *ω-3 FAD* gene structures (A) and alternative splicing transcripts of *ShFAD7a*/*b* containing a 5’UTR intron (B, S1 Fig) were generated on GSDS2.0 [60]. Exons, introns, and 5’UTR / 3’UTR are shown as yellow rectangles, black lines, and blue rectangles, respectively. Introns in phases 0, 1, and 2 are represented by the numbers 0, 1, and 2, respectively.

We isolated 2 heterozygous alleles containing some SNPs for *PfFAD3*, *PfFAD7* and *PfFAD8* from perilla, whereas 2 distinct *ShFAD3* genes, 2 heterozygous *ShFAD7* alleles, and 1 *ShFAD8* gene were cloned from chia (S1 Fig; Table 1). Full-length cDNAs of the *ω-3 FAD* genes from the 2 species, except for *PfFAD8b* (5’UTR and partial CDS included), were obtained with the longest mRNAs of 1,435~1,957 bp, 5’UTRs of 34~380 bp, ORFs of 1,152~1,323 bp, and 3’UTRs of 210~325 bp (Table 1). Except for *PfFAD3a* and *PfFAD7b*, the other *ω-3 FAD* members/alleles from the 2 species contained 2 to 6 alternative transcription start sites. *PfFAD3a*/*b*, *ShFAD3-1*/*-2*, *ShFAD7a*/*b*, and *ShFAD8* harbored 2 to 8 alternative poly(A) tailing sites, and *PfFAD7a*/*b* and *PfFAD8a* had 1; 1 to 2 typical and non-typical polyadenylation signals were present in these genes. Corresponding genomic DNAs were amplified using the total genomic DNA of leaves from the 2 species as a template, but amplification of genomic sequences for *PfFAD3b* and *PfFAD7b* failed. As shown in Fig 1, these sequences were 2,563~3,691 bp in length and all contain 8 exons and 7 introns with identical intron phases in the corresponding introns (Fig 1). The 7 introns all contained standard GT…AG splicing boundaries (S1 Fig). *ShFAD7a* and *ShFAD7b* were highly conservative in the length of introns 1 to 7 as there are 2 alleles for 1 *FAD7* gene, and the *ω-3 FAD* genes of the other members varied to some extent (Fig 1).

**Table 1:** Basic parameters of *ω-3 FAD* mRNAs from chia and perilla.

mRNA name	Longest mRNA (bp)	ORF and position (bp)	Transcription start sites ^a^	Length of 5’UTR (bp)	Poly(A) tailing sites	Length of 3’UTR (bp)	Polyadenylation signal ^b^
*PfFAD3a*	1,445	1,176	G_1_	49	T_1,399_, T_1,400_, G_1,426_, T_1,445_	174, 175, 201, 220	A_1,402_ATAAA
		50–1,225					
*PfFAD3b*	1,435	1,176	**G**_**1**_, **A**_**6**_, **G**_**10**_, G_28_	49, 44, 40, 22	T_1,418_, T_1,419_, **T**_1,427_, T_1,432_,	193, 194, 202, 207,	A_1,401_ATAAA
		50–1,225			**G**_1,435_	210	
*PfFAD7a*	1,866	1,317	**G**_**1**_, A_3,_ **G**_**90**_, G_118_,	308, 306, 219, 191,	T_1,866_	241	A_1,835_ATAAA
		309–1,625	G_292_	17			
*PfFAD7b*	1,866	1,317	G_1_	308	T_1,866_	241	A_1,835_ATAAA
		309–1,625					
*PfFAD8a*	1,957	1,317	G_1_, G_10_	380, 371	C_1,957_	260	T_2,747_ATAAA
		381–1,697					G_2,787_ATAAA
*PfFAD8b*	853	479	G_1_, G_10_, G_46_,	374, 365, 329			
		375–853					
*ShFAD3-1*	1,498	1,182	**C**_**1**_, A_7_	34, 28	T_1,374_, T_1,379_, T_1,405_, T_1,429_,	158, 163, 189, 213,	A_1,403_ATAAA
		35–1,216			T_1,465_, **T**_1,488_, T_1,490_, T_1,498_	249, 272, 274, 282	
*ShFAD3-2*	1,495	1,152	T_1_, G_70_, **C**_**72**_, A_74_	125, 56, 54, 52	T_1,492_, C_1,495_	215, 218	A_1,471_ATAAA
		126–1,277					
*ShFAD7a*	1,850	1,323	G_1_, A_146_, **G**_**192**_	202, 57, 11	**G**_1,745_, G_1,748_, G_1801_, **C**_1,824_,	220, 223, 276, 299,	T_1,725_ATAAA
		203–1,525			C_1,850_	325	A_1,812_ATAAT
*ShFAD7b*	1,825	1,323	G_1_, A_117_, G_192_	202, 86, 11	**C**_1,811_, C_1,825_	286, 300	T_1,711_ATAAA
		203–1,525					A_1,799_ATAAT
*ShFAD8*	1,794	1,290	A_1_, A_128_, T_131_, C_161_,	207, 80, 77, 47,	C_1,604_, C_1,678_, T_1,740_, G_1,747_,	107, 181, 243, 250	A_1,643_ATTAA
		208–1,497	A_166_, C_201_	42, 7	C_1,794_	297	T_1,716_ATAAA

^a^ Major types of alternative transcription start sites or poly(A) tailing sites are in bold face.

^b^ Typical polyadenylation signal “AATAAA” and a non-typical signal containing a substituted nucleotide are included.

The *ω-3 FAD* genes from the 2 species had higher G+C contents in ORFs (44.65~50.69%) than in 5’UTRs (38.78~47.06%), 3’UTRs (27.95~36.00%), and introns (20.20~40.62%) (S2 Table). In the *PfFAD8a* 5’UTR, there were 2 purine-stretches (23 bp and 24 bp) and 2 pyrimidine-stretches (21 bp and 31 bp), and *PfFAD8b* harbored a purine-stretch (24 bp) as well as 2 pyrimidine-stretches (25 bp and 27 bp) in its 5’UTR (S1 Fig). Pairwise alignment of 5’RACE cDNAs and genomic sequences of *ShFAD7a*/*b* revealed that a small intron (148 bp) was present in the 5’UTR of alternative splicing variants *ShFAD7a’* and *ShFAD7b’*, and it was 15 bp upstream of the start codon ATG, with standard GT…AG splicing boundaries (Fig 1; S1 Fig). However, the full-length cDNAs of *ShFAD7a’* and *ShFAD7b’* could not be isolated, which might be caused by the low abundance of these 2 splicing variants.

Pairwise-alignment of full-length mRNAs showed that *PfFAD3a*/*b* and *ShFAD3-1*/*-2* shared 61.8~62.2%, and 62.0~62.6% identity with *AtFAD3*, respectively (S3 Table). *PfFAD3a* was 98.0% identical to *PfFAD3b*, *ShFAD3-1* showed 86.3% identity with *ShFAD3-2*, and *PfFAD3a/b* shared 78.4~80.3% similarity to *ShFAD3-1*/*-2*. *PfFAD7a*/*b* and *ShFAD7a*/*b* shared 64.7~65.4% and 63.1~65.2% identity with *AtFAD7*/*8*, respectively (S3 Table). *PfFAD7a* was 99.8% identical to *PfFAD7b* with only 3 nucleotide changes (A-36-G, A-584-T and T-1,690-C, S2 Fig), *ShFAD7a* showed 96.4% identity with *ShFAD7b*, and *PfFAD7a*/*b* shared 79.8~80.1% similarity with *ShFAD7a*/*b*. *PfFAD8a*/*b* and *ShFAD8* shared 56.5~66.9% and 64.6~65.9% identity with *AtFAD7*/*8*, respectively (S3 Table). *PfFAD8a* was 95.7% identical to *PfFAD8b*, and *PfFAD8a* and *PfFAD8b* showed 78.4% and 73.3% identity with *ShFAD8*, respectively. Obviously, *PfFAD7a*/*b* showed a higher similarity to *ShFAD7a*/*b* than to *PfFAD8a*/*b*, whereas *PfFAD8a*/*b* shared higher identity with *ShFAD8* than with *PfFAD7a*/*b*. The sequence multi-alignment indicated that the identity of *ShFAD3-2* and *ShFAD8* corresponded to the previously reported chia Δ-15 desaturase partial gene (*ShΔ15*, 1,140 bp) and ω-3 desaturase partial gene (*Shω3*, 234 bp), respectively [4]. Previous studies [12–14] have characterized perilla *PfrFAD3-1* (NCBI accession No. AF047039.1), *PfrFAD3-2* (KX228917.1), *PfrFAD7-1* (U59477.1) and *PfrFAD7-2* (KP070824.1), which corresponded to *PfFAD3b*, *PfFAD3a*, *PfFAD7a*/*b* and *PfFAD8a*/*b* cDNAs in this study, respectively. However, these *ω-3 FAD* gene clones contained several SNPs because of different perilla cultivars or varieties from Korea and China (S2 Fig).

### Characterization of deduced ω-3 FAD proteins from chia and perilla

**Fig 2:**
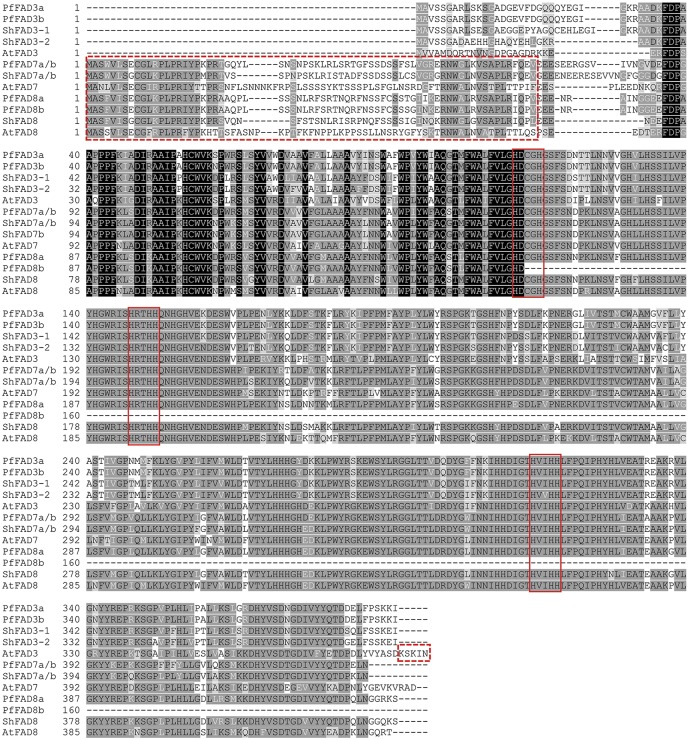
Multiple amino acid sequence alignment of ω-3 FADs from chia, perilla and *Arabidopsis*. The ω-3 FAD protein sequences among these 3 species were multi-aligned with the ClustalW method using the Vector NTI Advance 11.51 program. The predicted chloroplast transit peptide in the N-terminal and ER retrieval motifs in C-terminal are shown in the red dashed boxes. Three typical histidine boxes (motifs) found specifically in the membrane-bound FAD domain are indicated in red boxes.

Chia and perilla *ω-3 FAD* genes are more conserved at the amino acid (aa) level than at the nucleotide level (Table 2; Fig 2; S3 Table). PfFAD3a and PfFAD3b contained 391 aa with only 1 aa difference (S-66-N). PfFAD7a and PfFAD7b both were 438 aa in length and had completely identical aa sequences due to only 1 change in the degenerate codon GCA_584_-GCT_584_ within their coding regions. ShFAD7a and ShFAD7b both had 440 aa and possessed completely identical aa sequences because 11 SNPs in their ORFs all coincidentally located in degenerate codons. Hence, PfFAD3a/b, PfFAD7a/b, and ShFAD7a/b were considered as 3 heterozygous allele pairs, i.e. possibly in the gamete/genome they are all single-copy. PfFAD8a, ShFAD3-1, ShFAD3-2, and ShFAD8 were 438, 393, 383, and 429 aa in length, respectively. PfFAD3a/b, PfFAD7a/b, PfFAD8a/b, ShFAD3-1/-2, ShFAD7a/b, and ShFAD8 had a theoretical MW of 17.99~50.16 kDa and a predicted pI value of 7.50~9.51. PfFAD3a/b and ShFAD3-1/-2 showed 66.1~66.4% identity with AtFAD3. PfFAD7a/b and ShFAD7a/b, and PfFAD8a/b and ShFAD8 shared 71.7~74.8% and 55.5~73.9% identity with AtFAD7/8, respectively. PfFAD3a/b was 85.4~88.5% identical to ShFAD3-1/-2, PfFAD7a/b was 87.8% identical to ShFAD7a/b, and PfFAD8a/b showed 78.8~87.9% identity with ShFAD8. Similar to the situation of mRNA analysis, we found that ShFAD7a/b also had higher identities with PfFAD7a/b than with ShFAD8, whereas ShFAD8 also showed higher similarity to PfFAD8a/b than to ShFAD7a/b.

**Table 2:** Predicted primary parameters of deduced ω-3 FAD proteins from chia and perilla.

Name	Amino acids (aa)	MW (kDa)	pI	Conserved domain (SMART)		Subcellular location		Transmembrane helices	Phosphorylation sites
				Name	Position	Location	cTP*	(TOPCONS)	(NetPhos 2.0)
PfFAD3a	391	44.90	8.93	DUF3474	1–82	-	-	TM1: 67–87, TM2: 92–112,	S: 11, T: 3, Y: 5
				FA_desaturase	87–349			TM3: 226–246, TM4: 252–272	
PfFAD3b	391	44.93	8.93	DUF3474	1–82	-	-	TM1: 67–87, TM2: 92–112,	S: 11, T: 3, Y: 5
				FA_desaturase	86–349			TM3: 226–246, TM4: 252–272	
PfFAD7a/b	438	50.16	8.78	DUF3474	1–133	chloroplast	66	TM1: 119–139, TM2: 144–164,	S: 13, T: 2, Y: 4
				FA_desaturase	115–400			TM3: 278–298, TM4: 305–325	
PfFAD8a	438	50.01	9.13	DUF3474	1–128	chloroplast	70	TM1: 115–135, TM2: 138–158,	S: 7, T: 2, Y: 4
				FA_desaturase	134–394			TM3: 273–293, TM4: 299–319	
PfFAD8b	159	17.99	9.51	DUF3474	1–128	chloroplast	70	TM1: 114–134, TM2: 138–158	S: 7, T: 2, Y: 4
ShFAD3-1	393	44.87	7.50	DUF3474	2–84	-	-	TM1: 69–89, TM2: 94–114,	S: 14, T: 3, Y: 6
				FA_desaturase	89–351			TM3: 228–248, TM4: 253–273	
ShFAD3-2	383	43.98	7.53	DUF3474	1–74	-	-	TM1: 59–79, TM2: 84–104,	S: 11, T: 4, Y: 6
				FA_desaturase	79–341			TM3: 218–238, TM4: 244–264	
ShFAD7a/b	440	49.79	8.42	DUF3474	1–135	chloroplast	64	TM1: 59–79, TM2: 84–104,	S: 12, T: 2, Y: 3
				FA_desaturase	141–403			TM3: 218–238, TM4: 244–264	
ShFAD8	429	48.72	9.08	DUF3474	1–119	chloroplast	49	TM1: 104–124, TM2: 130–150,	S: 7, T: 3, Y: 4
				FA_desaturase	124–385			TM3: 264–284, TM4: 290–310	

* cTP, chloroplast transit peptide in the N-terminus of the ω-3 FAD proteins.

The SMART and Pfam database search revealed that ω-3 FAD proteins from perilla and chia possessed conserved domains DUF3474 (Pfam: PF11960) and FA_desaturase (PF00487; partial PfFAD8b not included) (Table 2). An uncharacterized DUF3474 domain present in bacteria and eukaryotes was found to be associated with the FA_desaturase domain [66]. Similar to *Arabidopsis* ω-3 FAD proteins, except for PfFAD8b, each member contained 3 histidine boxes (Fig 2), including HDCGH, HRTHH and HVI(V)HH, which could play essential roles in maintaining desaturase activity and forming a part of the di-iron center in which oxygen activation and hydrogen subtraction occur [67]. Unlike AtFAD3, ShFAD3-1/-2 and PfFAD3a/b lacked a C-terminal ER retrieval signal (-KSKIN, Fig 2). ChloroP 1.1 [68] predicted that an N-terminal chloroplast transit peptide (cTP, 49~70 aa) was located in PfFAD7a/b, PfFAD8a/b, ShFAD7a/b and ShFAD8, and TargetP 1.1 [69] predicted that they were targeted to the chloroplasts, similar to other FAD7/8s (Fig 2; Table 2). Predicted by TOPCONS, partial PfFAD8b had 2 strong transmembrane helices, and the other ω-3 FAD proteins of the 2 species contained four (S3 Fig; Table 2). NetPhos2.0 predicted 13–23 potential phosphorylation sites in each allele/member (7~14 S, 2~4 T, and 3~6 Y) (Table 2). Analyzed by SOPMA [70], ω-3 FAD proteins of the 2 species contained 30.43~35.45% α-helices, 14.55~21.15% extended strands, 6.29~16.45% β-turns and 30.29~45.91% random coils (S4 Fig). Alfa-helix and random coil resided in the main body of their secondary structures.

### Molecular evolution analysis

**Fig 3:**
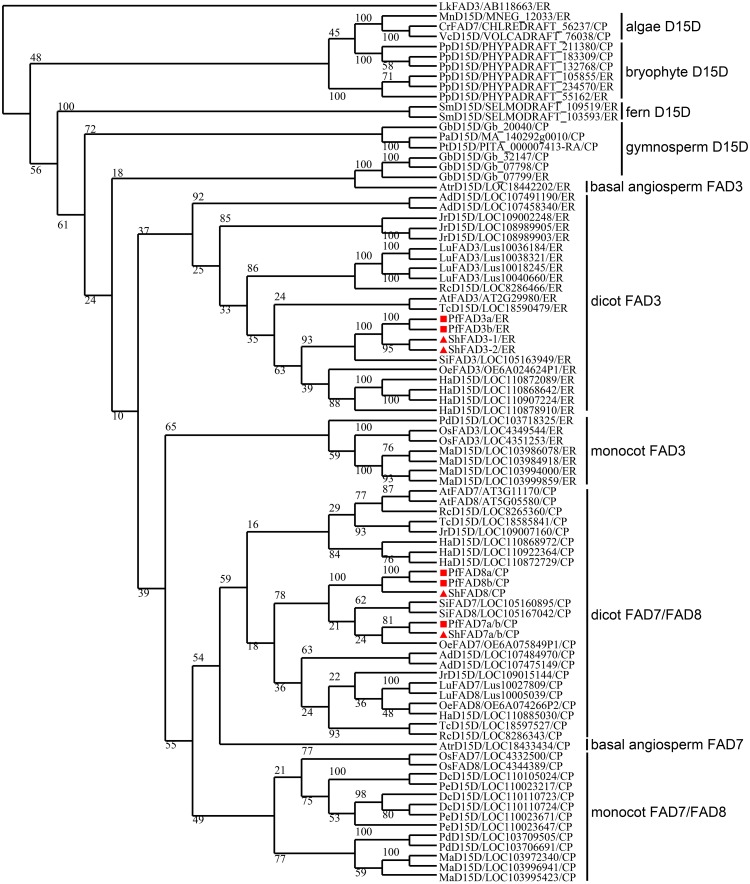
Phylogenetic relationships of plant ω-3 FAD proteins. The ω-3 FAD proteins from chia, perilla, and other plants were multi-aligned using the MAFFT7 program with default parameters. The phylogenetic tree was constructed by using the SeaView 4.0 with the BioNJ method (1000 bootstrap replicates). The organism name, accession numbers and subcellular predictions of other plant ω-3 FAD proteins are shown in S4 Table. The ω-3 FAD proteins from chia and perilla are indicated by the red squares and red triangles, respectively.

To determine the phylogenetic relationship of the ω-3 FAD proteins from chia, perilla and other plants, a phylogenetic tree was constructed using the BioNJ method in the SeaView 4.0 program with *Lachancea kluyveri* FAD3 as the out-group. As shown in Fig 3, plant ω-3 desaturases were divided into 10 subgroups: algae D15D, bryophyte D15D, fern D15D, gymnosperm D15D, basal angiosperm FAD3, dicot FAD3, monocot FAD3, dicot FAD7/FAD8, basal angiosperm FAD7, and monocot FAD7/FAD8. In these subgroups, chia and perilla FAD3s and FAD7s/FAD8s were clustered into the specific dicot FAD3 subgroup and dicot FAD7/FAD8 subgroup, respectively, demonstrating their phylogenetic origin. Chia and perilla FAD3s were first grouped together with Lamiales microsomal ω-3 FADs such as sesame SiFAD3 and olive OeFAD3, then with FAD3s from non-Laniales species such as sunflower, cocoa, *Arabidopsis*, etc. Chia and perilla FAD7s/8s were first clustered with Lamiales plastidial ω-3 FADs such as sesame SiFAD7/8 and olive OeFAD7, then with FAD7s/8s from non-Laniales species such as sunflower, wild peanut, chestnut, etc.

The tree also reveals some important evolution clues of plant ω-3 FADs. The first is the convergent evolution of FAD7/FAD8, i.e. multiple independent origination of plastidial ω-3 FADs during plant divergence process. FAD7s/FAD8s across different phylums do not cluster together to form a plant FAD7s/FAD8s large group. Rather, in most cases FAD7s/FAD8s were originated from FAD3s within respective phylums. For example, the FAD7s/FAD8s from dicot, monocot, and basal angiosperm plants form a large group, which is diverged from angiosperm FAD3s. On the other hand, this implies that dicot and monocot FAD7s/FAD8s share a common basal angiosperm FAD7 ancestor. The second is the amplification of gene numbers of ω-3 FADs in non-algae plants. Many plants contain more than 1 copy of FAD3 and/or FAD7/FAD8, especially in *Physcomitrella patens*, sunflower, flax, *Arachis duranensis*, and *Musa acuminata* subsp. *malaccensis*. The third is noticeable evolution features of ω-3 FADs in gymnosperms. *Picea abies* and *Pinus taeda* both contain only 1 plastidial ω-3 FAD and no ER-type FAD, while *Ginkgo biloba* contain both types. Besides, the 2 genes newly duplicated from ginkgo *FAD3* contain a chloroplast transit peptide, indicating their directional evolution of subcellular localization after recent gene duplication events.

### Organ-specificity of *ω-3 FAD* gene family from chia and perilla

**Fig 4:**
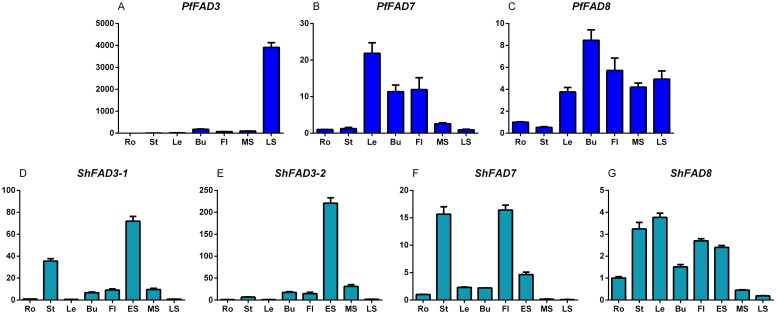
Organ-specificity expression profiles of *ω-3 FAD* gene family from chia and perilla (A-G). Relative expression levels were determined by qRT-PCR with the *25SrRNA* gene as an internal control. The expression value in root was set to “1”. The values represent the average ±SD of 3 biological replicates. Ro, root; St, stem; Le, leaves; Bu, bud; Fl, flower; ES, early-stage seeds (10 days after flowering); MS, mid-stage seeds (20 days); LS, late-stage seeds (30 days).

To shed light on the biological function of the *ω-3 FAD* genes from perilla and chia, their organ-specificity patterns were investigated. In this study, we firstly validated the specificity of the qRT-PCR primers using agarose gel electrophoresis of their PCR products, which all showed a predicted size of specific band for each *ω-3 FAD* mRNA, without cross amplification using templates of other genes. Then, qRT-PCR was carried out to examine the expression patterns of chia and perilla *ω-3 FAD* genes in various organs. The results showed that *ω-3 FAD* genes from perrila and chia were expressed in various organs, but with different levels (Fig 4A–4G). *PfFAD3* was mainly expressed in late-stage seeds (approximately 4000-fold compared to the roots). The expression level of *ShFAD3-1* was relatively higher in stems and early-stage seeds than in other organs, while *ShFAD3-2* transcripts mainly accumulated in early-stage seeds. The *PfFAD7* expression levels in leaves, buds, and flowers were higher than in other organs. A smaller amount of *PfFAD8* mRNA was accumulated in the roots and stems compared to other tissues. *ShFAD7* was transcribed more in stems and flowers, less in middle-stage and late-stage seeds, and moderately in other organs. Except for middle-stage and late-stage seeds, *ShFAD8* was expressed at relatively high levels in other tissues. These levels were relatively lower than the levels obtained in *ShFAD7*. From this expression analysis, we found that *FAD7* genes were expressed at higher levels than *FAD8* in both chia and perilla, which was also similar to the outcomes for other species, e.g., *D*. *sophia* [45] and purslane [28].

### Expression profiles of *ω-3 FAD* gene family from chia and perilla under various stress treatments

**Fig 5:**
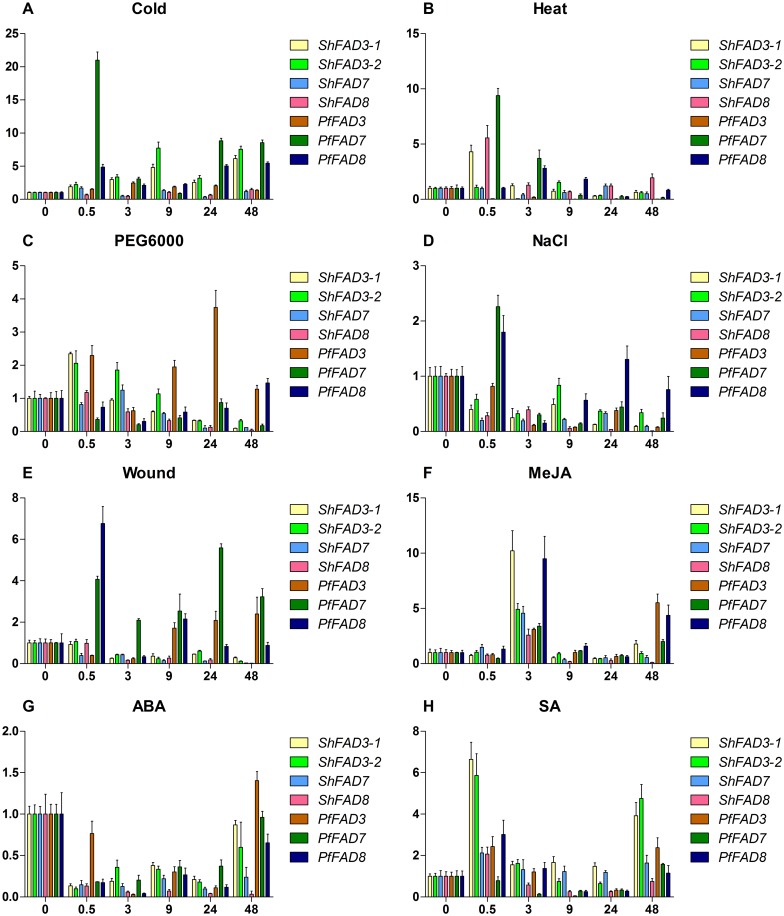
Expression patterns of chia and perilla *ω-3 FAD* genes in seedling leaves under stress treatments. Eight stresses, including cold (4°C, A), heat (B, 38°C for chia and 42°C for perilla), PEG6000 (10% w/v, C), NaCl (300 mM, D), wounding (E), MeJA (100 μM, F), ABA (100 μM, G) and SA (1 mM, H), were used, and the corresponding treatment times were 0, 0.5, 3, 9, 24, and 48 h. Relative expression levels were determined by qRT-PCR with the *25SrRNA* gene serving as an internal control, and the expression value at 0 h was set to “1”. The values represent the average ±SD of 3 biological replicates.

To examine the relationship between chia and perilla *ω-3 FAD* genes under various stresses, real-time qRT-PCR was performed to illustrate the expression profiles of the *ω-3 FAD* genes from the 2 species in seedling leaves under the biotic stress of wounding and abiotic stresses, including cold, heat, drought (PEG6000), salt (NaCl), and treatments with plant hormones including MeJA, ABA, and SA. As shown in Fig 5A–5H, each *ω-3 FAD* gene responded to multiple treatments. Under cold treatment, *PfFAD7* expression was rapidly and transiently up-regulated to 20-fold at 0.5 h, then dropped down and fluctuated, but still keeping a significant up-regulation level. *PfFAD8* was weaker in response to cold stress than *PfFAD7* (Fig 5A). However, both *ShFAD7* and *ShFAD8* showed very limited or non-distinct up-regulation. Interestingly, unlike *PfFAD3* which showed only a little up-regulation by cold, *ShFAD3-1/2* was steadily up-regulated by cold, though not as quick as *PfFAD7* and *PfFAD8* (Fig 5A). The *PfFAD3* transcript was quickly reduced to the lowest level for the whole term of heat treatment (Fig 5B). *ShFAD3-1* and *PfFAD7/8* expression were up-regulated a short time after heat treatment, but their transcripts were inhibited over the long term. *ShFAD8* expression transiently peaked at 0.5 h, but quickly dropped down to constant levels (Fig 5B). *ShFAD3-2* expression was least sensitive to cold treatment, with only a little down-regulation at 24 h and 48 h. Under drought stress (PEG treatment), *ShFAD3-1*/*-2* expression firstly increased and then decreased, whereas *PfFAD3* showed fluctuation with rounds of up-regulation and falling back. Generally, *FAD7* and *FAD8* genes from both perilla and chia were down-regulated by PEG treatment, though *PfFAD8* was less sensitive (Fig 5C). Under NaCl treatment, all *ω-3 FAD* genes from perilla and chia showed down-regulation with trends similar to those in PEG treatment, though *PfFAD7* and *PfFAD8* were transiently up-regulated at 0.5 h and *PfFAD8* was less sensitive than others (Fig 5D). Wounding stress inhibited the expression of all chia *ω-3 FAD* genes, but it enhanced *PfFAD7* transcripts (Fig 5E). After wounding stress, *PfFAD8* showed fluctuation with rounds of up-regulation and falling down, while *PfFAD3* expression was first down-regulated and then up-regulated in long term (Fig 5E). After MeJA and SA treatments, all perilla and chia *ω-3 FAD* genes showed similar dynamics, i.e. transient up-regulation followed by declining and re-up-regulation at 48 h, but SA stimulation (peaked at 0.5 h) was quicker than MeJA (peaked at 3 h) (Fig 5F/5H). Under ABA treatment, the expression of all perilla and chia *ω-3 FAD* genes was inhibited, but 48 h after treatment most of them resumed to be around basal levels except *ShFAD7/8* which still kept inhibition status (Fig 5G). These results suggest that chia and perilla *ω-3 FAD* genes all are responsive to various stresses and might play some roles in coping with adversities, but inter-specific differences between chia and perilla as well as inter-genic differences especially between FAD3 and FAD7/8 are distinct.

### Catalytic activity identification of ω-3 FADs in chia and perilla using yeast expression

**Fig 6:**
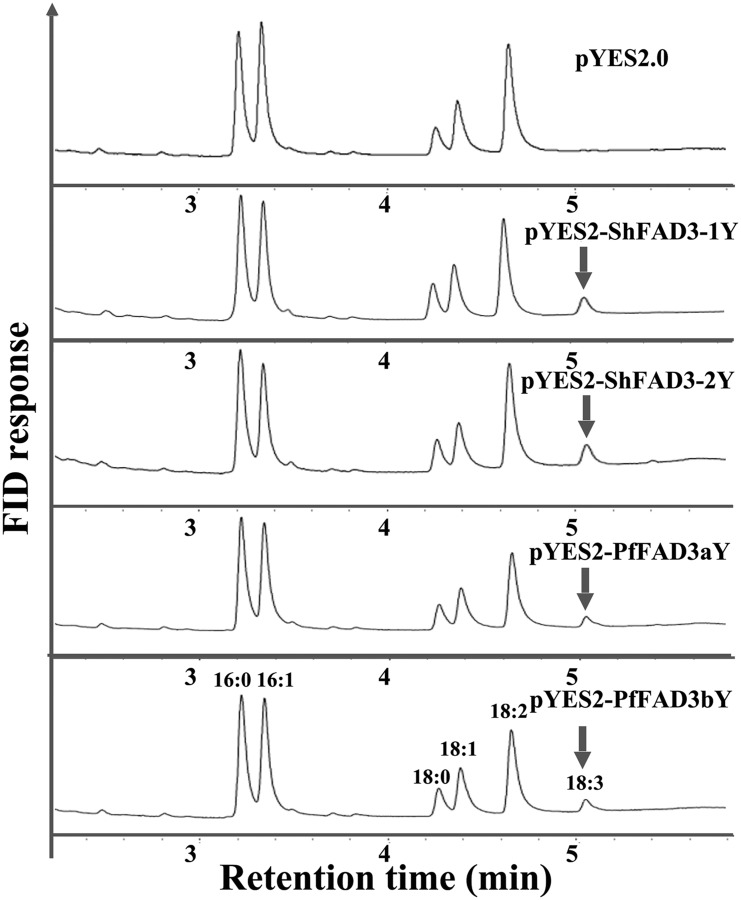
Gas chromatographic (GC) analysis of fatty acid composition of transgenic yeast strains transformed with pYES2.0, pYES2-ShFAD3-1Y, pYES2-ShFAD3-2Y, pYES2-PfFAD3aY, and pYES2-PfFAD3bY. The arrowhead shows the novel peak of α-linolenic acid (ALA). 16:0, palmitic acid; 16:1^Δ9^, palmitoleic acid; 18:0, stearic acid; 18:1^Δ9^, oleic acid; 18:2^Δ9,12^, linoleic acid; 18:3^Δ9,12,15^, α-linolenic acid.

Yeast has been shown to be an ideal model system for identifying the function of ER-located desaturases, including FAD2 and FAD3 [17,71], but it was not suitable for heterologous expression of plastidial desaturases (e.g. FAD6/7/8) due to their requirements for electron transport chains from the chloroplast [16]. To determine the function of *PfFAD3a*, *PfFAD3b*, *ShFAD3-1* and *ShFAD3*-2, corresponding ORFs were individually cloned into the expression vector pYES2.0 under an inducible GAL1 promoter and transformed into *S*. *cerevisiae*. The GC analysis of FA compositions in transformed yeast strains showed a high content of LA that is absent in wild-type yeast, which confirmed the correct uptake of supplemented substrate. As shown in Fig 6, ALA was not present in yeast cells transformed with empty vector pYES2.0, but ALA production was detected in yeast cells transformed with pYES2-ShFAD3-1Y, pYES2-ShFAD3-2Y, pYES2-PfFAD3aY, and pYES2-PfFAD3bY. The percentage of ALA in transgenic yeast cells was 8.84~16.91% of the total FA, and the conversion of LA to ALA was 8.84~16.91% (Table 3); the desaturation ratio of PfFAD3a/b was not as high as that of ShFAD3-1/-2. This result showed that yeast cells overexpressing *PfFAD3a*, *PfFAD3b*, *ShFAD3-1*, and *ShFAD3-2* performed the desaturation of LA to ALA, implying that these four *FAD3* genes all encode a functional linoleate Δ-15 desaturase. Unfortunately, ALA production was not detected in yeast cells transformed with recombinant vectors pYES2-PfFAD7-T2A-BnFD2, pYES2-PfFAD8-T2A-BnFD2, pYES2-ShFAD7-T2A-BnFD2, and pYES2-ShFAD8-T2A-BnFD2, although various conditions had already been optimized according to current theories and this experiment was repeated for many times.

**Table 3:** Fatty acid composition of *S*. *cerevisiae* strains overexpressing chia *ShFAD3* and perilla *PfFAD3* genes.

Plasmid	Fatty acid composition (mol %)						Conversion (%)
	16:0	16:1^Δ9^	18:0	18:1^Δ9^	18:2^Δ9,12^	18:3^Δ9,12,15^	
pYES2	24.18±0.49	30.31±0.18	6.18±0.23	13.13±0.48	26.19±0.19	-	-
pYES2-PfFAD3aY	23.77±0.39	29.92±0.33	5.93±0.02	12.96±0.26	24.99±0.70	2.42±0.44	8.84±1.69
pYES2-PfFAD3bY	25.47±0.97	30.16±0.15	6.18±0.34	12.93±0.28	22.59±1.04	2.68±0.05	10.61±0.55
pYES2-ShFAD3-1Y	25.94±0.10	25.17±1.40	7.48±0.19	12.76±0.77	25.35±2.29	3.30±0.06	11.60±1.04
pYES2-ShFAD3-2Y	24.95±0.15	24.20±1.04	6.95±0.21	13.04±0.61	25.65±1.56	5.20±0.59	16.91±2.22

The full names of the FAs are described in Fig 6. The data are the mean ± SD from3 biological replicates; “-”, no detection.

## Discussion

### Evolutionary features of *ω-3 FAD* genes from chia, perilla, and plant kingdom

In this study, we have systematically isolated and characterized the *ω-3 FAD* gene family from chia and perilla. Chia *ShFAD3* contains 2 member genes, including *ShFAD3-1* and *ShFAD3-2*, and a single *ShFAD8* gene was cloned from chia, while *ShFAD7* and perilla *PfFAD3*/*7*/*8* are also single-gene loci possessing 2 heterozygous allele sequences. For all chia *ω-3 FAD* genes, i.e. *ShFAD3-1/2*, *ShFAD7a/b*, and *ShFAD8*, we have obtained their both full-length cDNAs and corresponding gDNAs. For all perilla *ω-3 FAD* genes, i.e. *PfFAD3a/b*, *PfFAD7a/b*, and *PfFAD8a/b*, we isolated their full-length cDNAs (except *PfFAD8b*), but we failed to obtain the genomic sequences of *PfFAD3b*/*7b*/*8b* though we used allele-specific primers to screen numerous TA-colonies in gDNA cloning process. Nevertheless, unlike the distinct divergence between *ShFAD3-1* and *ShFAD3-2* on both nucleotide and protein levels, the nucleotide sequences of *ShFAD7a*, *PfFAD3a*, *PfFAD7a*, and *PfFAD8a* are extremely similar to *ShFAD7b*, *PfFAD3b*, *PfFAD7b*, and *PfFAD8b*, respectively, with encoded proteins completely identical to each other or differed by only 1 to 2 similar aa substitution. Until now, no completed genome data from chia and perilla could be used as an important reference. Though traditional literatures report that chia is a diploid/amphiploid while perilla is an amphidiploid [1,3], the inter-sister identities within each of *PfFAD3a/b*, *PfFAD7a/b*, and *PfFAD8a/b* pairs are as high as that of *ShFAD7a/b* pair. This result is likely that these 4 pairs are 4 heterozygous allelic pairs other than 8 independent genes, and perilla might be originated from amphidiploidization between 2 subspecies or 2 closely related species. As 1 parent of perilla is *P*. *citriodora* [3], the other parent should be sought among wild or cultivated subspecies or species with very close relationships to *P*. *citriodora*. A simply feasible way to identify this unknown parent is to clone and compare the whole set of *PfFAD3a/b*, *PfFAD7a/b*, and *PfFAD8a/b* pairs from the candidate subspecies or species.

Sequence similarity of mRNAs and proteins showed that *ShFAD7a/b* were more identical to *PfFAD7a/b* than *ShFAD8*, whereas *ShFAD8* was more similar to *PfFAD8a/b* than *ShFAD7a/b*. The evolutionary relationship showed that *ShFAD7a/b* was clustered with *PfFAD7a/b*, and *ShFAD8* was clustered with *PfFAD8a/b*. These results suggested that, in Lamiaceae family, the gene duplication event leading to *FAD7* and *FAD8* was prior to the divergence between genus *Salvia* and genus *Perilla*, i.e. possibly the whole Lamiaceae family has evolved 2 plastidial *ω-3 FAD* genes, *FAD7* and *FAD8*. Finally, though *FAD7* and *FAD8* from Lamiaceae are not respective real orthologues of *FAD7* and *FAD8* from Brassicaceae, since they are the results of respective duplications after divergence between order Lamiales and order Brassicales, we still prefer to name them as *FAD7* and *FAD8* other than as two *FAD7* genes as reported previously [13]. There are 3 reasons. Firstly, on both nucleotide and protein levels, they differ from each other significantly. Secondly, in BLAST analyses, *FAD7* and *FAD8* from perilla and chia show a little deviation toward *FAD7* and *FAD8* from *A*. *thaliana* respectively. Thirdly, many previous reports already adopted this method to name the duplicated plastidial *ω-3 FAD* genes from non-Brassicales plants.

Though the evolution of front-end desaturases as a whole has been reported previously, to date there is no systemic study on the evolution of *ω-3 FAD* genes. In studying safflower *ω-3 FAD* genes, Guan et al. constructed a phylogenetic tree of ω-3 desaturases from different plants, but the tree was still not systemic enough and they did not deeply analyse the evolution rules [27]. On the other hand, sequencing, annotation, and releasing of more and more plant genomes make it possible in this research to thoroughly identify the evolutionary features of ω-3 desaturases from various plant taxa. All the 3 algae species own a single copy ω-3 desaturase gene, either ER-type or CP-type, while all non-algae plants contain more than 1 except for the conifer species *P*. *abies* and *P*. *taeda*. In microalga *Chlamydomonas reinhardtii*, the single *FAD7* gene can impact both plastidic and extraplastidic membrane lipids [72], whether the single gene status of other algae and conifer plants have also evolved similar mechanisms deserve future study. On the contrary to these species, most plant species contain both ER-type and CP-type *D15D* genes, which means that divergently evolution and keeping of both types are necessary for most higher plants. However, our phylogenetic study distinctly indicates that plant *FAD7/FAD8* genes are convergently evolved in respective phylums, i.e. origin of *FAD7/FAD8* genes through gene duplication events after the formation of individual phylums. Furthermore, recent duplication events of *FAD3* or *FAD7/FAD8* genes in certain lower taxa are very common, i.e. in *P*. *patens*, ginkgo, wild banana, flax, sunflower, etc.

### Noticeable structural features of *ω-3 FAD* genes from chia and perilla

We found that exon/intron numbers, intron phases, and splicing boundaries between *ω-3 FAD* genes from chia, perilla, and Arabidopsis were highly conserved (Fig 1) [30,31,73], which indicates that they were derived from a common ancestral gene. Except for the partial PfFAD8b, the remaining ω-3 FADs from the two species all contain 3 histidine boxes that are essential for maintaining their catalytic activity [33], a conserved domain FA_desaturase (pfam PF00487), and 4 strong transmembrane helices, which are typical characteristics of plant membrane-bound desaturases [74,75]. Alternative splicing is an important regulatory mechanism for controlling gene expression at a post-transcriptional level, and intron retention is more prevalent in plants [76]. A pairwise alignment showed that the alternative splicing variants *ShFAD7a’/b’* contained 1 5’-UTR intron, which was consistent with soybean *GmFAD8* [17], and both variants had intron retention, but the corresponding full-length cDNA sequences could not be isolated from chia leaves. This might result from the relative low abundance of *ShFAD7a’*/*b’* transcripts due to certain environmental stress factors or during different development stages of chia. Under cold treatment, *ShFAD7a/b* expression is more similar to *GmFAD8-1* [17] compared the remaining *ω-3 FAD* genes in these 2 species, which is likely due to the existence of a 5’UTR intron. Additionally, as reported in previous studies [77,78], the 5’UTR intron also possessed promoter activity, which enhanced transcriptional expression of the target gene under various environmental factors. Therefore, it is necessary to carry out the isolation and characterization of these two *ShFAD7a*/*b* isoforms in a future study. Additionally, there were 1 to 2 purine-stretches and 2 pyrimidine-stretches in the 5’UTRs of *PfFAD8a*/*b* (S1 Fig), which suggests their possible roles in modulating the transcription and translation of *PfFAD8* genes. Except for *PfFAD8b*, the remaining *ω-3 FAD* genes for 2 species have 1 to 2 canonical or non-canonical poly(A) signals in the 3’UTRs, which may play a crucial role in determining alternative poly(A) sites.

### Functional identification of *ω-3 FAD* genes from chia and perilla

In this study, heterologous yeast expression confirms the catalytic activity of FAD3s of chia and perilla, i.e. they both encode a functional linoleate Δ-15 desaturase. The conversion ratio of ALA (8.84~10.61%) in yeast overexpressing *PfFAD3a/b* was higher than that of *PfrFAD3* (3.89~6.00%) described in previous reports [13,79], which might be due to a modified Kozak sequence containing 6 adenine nucleotides (AAAAAAATG, S1 Table) [80] in this *PfFAD3a*/*b* yeast expression system. In addition, the higher conversion ratio of LA to ALA obtained for ShFAD3-1/-2 compared to PfFAD3a/b in transformed yeast suggest that ShFAD3-1/-2 contributes to the ALA content to a greater extent than PfFAD3a/b does. Additionally, previous reports indicated that both the removal of N-terminal chloroplast transmit peptide and the ferredoxin co-expression were necessary to increase heterologous expression activity of plant plastidial FAD7/8 desaturases in yeast [23,24]. Here, although chloroplast transmit peptides of both PfFAD7/8 and ShFAD7/8 were deleted and rapeseed ferredoxin BnFD2 was meanwhile co-expressed, catalytic activity of the conversion of LA to ALA was not detected in transgenic yeast cells harbouring PfFAD7/8 or ShFAD7/8. However, functionality of perilla *PfrFAD7-1* and *PfrFAD7-2* genes were confirmed using their coding regions despite no deletion of chloroplast transmit peptide in N-terminus [13]. Hence, there is a need to identify the function of FAD7/8 of chia and perilla in yeast, using the original open reading frame without deletion of chloroplast transmit peptide, in the future study. More importantly, future study on yeast expression of plants *FAD7/FAD8* genes should further clarify the issues regarding to chloroplast transmit peptide and ferredoxin.

### Transcriptional expression characteristics of *ω-3 FAD* genes from chia and perilla

In higher plants, trienoic acids (TAs), including ALA, are structural components of membrane lipids and seed storage lipids and function as precursors of signaling molecules, e.g., JA [11,81]. FA unsaturation in the cellular membrane plays crucial role in temperature stress and adaption [82]. The JA-signaling pathway also functions in plant growth and development, as well as defense responses [83]. In Arabidopsis, the formation of TAs was catalyzed by two types of ω-3 FADs: ER-located FAD3 and chloroplast-located FAD7/8 [30,31,72]. To date, it has been reported that *ω-3 FAD* genes in a wide variety of plants function in ALA biosynthesis in various organs/tissues and in response to various environmental stimuli.

Organ-specificity expression showed that all members of chia and perilla *ω-3 FAD* genes were expressed in various organs, but there was divergence and complementation in their expression patterns. In perilla, *PfFAD3* mRNA accumulation in late-stage seeds was the most abundant (approximately 4000-fold of root, Fig 1A), which was not consistent with the seed-specific expression of *PfFAD3* [12] but imitated the expression pattern of *PfrFAD3* in various organs [13], revealing its key roles of ALA biosynthesis in seeds. Similar to *PfrFAD7-1*/*-2* [14,15], *PfFAD7*/*8* showed higher expression levels in leaves than seeds, which indicated that they play preferential roles in ALA accumulation of vegetative organs. *ShFAD3-1/-2* was mainly transcribed in early-stage seeds, whereas the difference between *ShFAD3-1* and *ShFAD3-2* could be attributed to higher transcripts of *ShFAD3-1* in stems than that of *ShFAD3-2*, which indicated that there was a small functional partition between the two copies of *ShFAD3*. A previous study [4] indicated that *ShΔ15* and *Shω-3* were mainly expressed in the early stages of seed development, which are almost in agreement with transcriptional pattern of *ShFAD3-2* and *ShFAD8*, respectively, and suggests that they both play an important role in ALA biosynthesis of early seed stages.

The current results indicate that in the chia seedling leaves, ER *ω-3 FAD* expression is tightly regulated under cold treatment, while no obvious change was detected for plastidial *ω-3 FAD*. This outcome is consistent with previous observations for soybean *GmFAD3A*, *GmFAD7-1*/*-2* and *GmFAD8-1*/*-2* [17]. In perilla, plastidial *ω-3 FADs* play more important roles in response to cold than ER *ω-3 FADs*. Heat treatment inhibits the *PfFAD3* transcripts but first up-regulates and then down-regulates *PfFAD7/8*, which suggests that low levels of TAs are critical for the heat response and tolerance over the long-term. At 48 h after heat treatment, chia *ShFAD3-1/-2* and *ShFAD7* transcripts were reduced slightly, whereas *ShFAD8* expression increased to a small extent, which indicates that *ShFAD8* might respond to heat treatment at a higher temperature or over a longer period. This hypothesis needs to be tested in further study. The different *ω-3 FAD* gene expression patterns between chia and perilla under cold and heat suggest that there is diversity between the two species for response time and speed in these opposite stresses. The lima bean *PlFAD3* transcript was induced by drought stress [42]; *ω-3 FAD* genes in the present study were also enhanced at different levels by drought. Salt stress suppressed chia *ω-3 FAD* and *PfFAD3* gene expression, which was similar to lima bean *PlFAD3* [42], but an up-regulated expression of *PfFAD7/8* at 0.5 h was obtained, which implied that processes, such as enhanced membrane FA unsaturation, are essential for perilla response to salt stress.

Wounding normally exemplifies biotic stresses, e.g., insect feeding and herbivory. Multiple structurally distinct molecules function in wound signaling, including plant hormones (e.g., JA, ABA and ethylene), oligosaccharides, and oligopeptides [84]. It has been reported that ALA for JA biosynthesis is derived from ER (FAD3) and plastid (FAD7/8) membranes [85]. In this study, *PfFAD3*/*7*/*8* expression was up-regulated by wound stress, which is consistent with the report on *ω-3 FAD* genes from *D*. *sophia* [45]. Wounds can activate the JA biosynthesis pathway and lead to an increase in JA accumulation by converting ALA to JA, which plays a critical role in the transcriptional regulation of wound-inducible genes [86,87]. However, wound treatment down-regulated the transcripts of chia *ω-3 FAD* genes. Previous reports showed that there is a complex wound signaling network in plants, which notably has species-specific variations [84]. Accordingly, the participation of any deduced signal in the activation of wound response depends on plant species, which indicates a different wound-induction mechanism for the *ω-3 FAD* genes in chia and perilla.

Plants have a set of defense mechanisms against microbial pathogen attacks in which plant hormones, e.g., JA, SA and ABA, play indispensable signaling roles [88]. In general, cooperative or antagonistic crosstalk between these hormones plays a pivotal role in maintaining the disease resistance [88]. Therefore, the induction of *ω-3 FAD* gene expression occurs under MeJA, SA and ABA treatment, which occurs due to ω-3 FAD products serving as precursors of JA biosynthesis. Under SA and MeJA treatment, *ω-3 FAD* genes from perilla and chia showed either up-regulated or down-regulated expression patterns, which indicates that they play important roles in the SA and JA signaling pathways. Moreover, ABA treatment suppressed the transcription of chia and perilla *ω-3 FAD* genes, which is almost consistent with *CsFAD7* and *CsFAD8* in the tea plant [49].

The different expression patterns observed between *FAD3* and *FAD7*/*8* under various stresses imply that there is an obvious divergence of ER-type and plastid-type *ω-3 FAD* genes in stress response and adaptation. This result also suggests that plants need to maintain membrane fluidity for each stress/treatment adaptation by modulating PUFAs (including ALA) compositions. In addition, the response variation between the two species may be associated with long-stage artificial domestication and the selection of cultivated species of chia and perilla in different places of origin, i.e., Mexico and Asia, respectively.

In conclusion, this is the first report to provide a systemic and comparative study of *ω-3 FAD* gene family from chia and perilla, which are two plant sources containing the most abundant ω-3 PUFAs (namely, ALA). In this study, we systemically isolated the ER-type *FAD3* and chloroplast-type *FAD7*/*8* genes from these two species and comparatively analyzed sequence characters, genomic organization, phylogenetic relationships, organ-specificity, stress-inducibility and enzymatic activities. This work provides a basis for revealing the molecular mechanism of high ALA traits via the FA desaturation pathway and facilitates our understanding of the chia and perilla *ω-3 FAD* genes in response to multiple stresses. Besides, this study also reveals some important evolution features of plant *ω-3 FAD* genes. In further study, it is very important to carry out transgenic manipulation of chia and perilla *ω-3 FAD* genes for exploring ALA traits in oilseed crops because these are dedicated steps for ALA biosynthesis.

## Supporting information

S1 Table Degenerate and non-degenerate primers used in this study. ^a^ N: A or G or C or T; V: A or G or C; D: G or A or T. ^b^ Both degenerated bases and the restriction sites that were introduced are underlined, and Kozak sequences [80] are italicized in bold face. ^c^ T2A sequence [65] is wave-lined, and overlapped regions between two primers are in bold face. All primers were synthesized by Genscript (Nanjing, China) and Sangon Biotech (Shanghai, China).
(DOCX)

S2 Table Basic parameters of *ω-3 FAD* genes from chia and perilla. (DOCX)

S3 Table Identities of mRNAs (italic) and proteins among ω-3 FADs from chia, perilla and Arabidopsis*. * Pairwise-alignment and identity analysis of mRNAs and proteins of ω-3 FADs from perilla, chia and Arabidopsis were performed using the ClustalW method in Vector NTI advance 11.5.1 (Invitrogen, USA).
(DOCX)

S4 Table Organism names, accession numbers and subcellular predictions of plant ω-3 FAD proteins used in this study. (XLSX)

S1 Fig Gene and protein sequences of *ω-3 FAD* gene family from chia and perilla (A-S). The start codon (ATG) and the stop codon (TAA, TAG and TGA) are in underlined in bold face. Alternative transcription sites and poly(A) tailing sites are underlined and italicized, and the major types are shown in bold face. The introns and typical and non-typical poly(A) signals are underlined. The purine-stretches (> 20 bp) and pyrimidine-stretches (> 20 bp) are highlighted by the gray background.
(DOCX)

S2 Fig Multi-alignment of perilla *ω-3 FAD* mRNAs in this study and previous reports. *PfrFAD3-1* mRNA (NCBI accession no AF047039.1), *PfrFAD3-2* mRNA (KX228917.1), *PfrFAD7-1* mRNA (U59477.1) and *PfrFAD7-2* mRNA (KP070824.1).
(DOCX)

S3 Fig Transmembrane helices of ω-3 FAD proteins from chia and perilla. They were predicted by TOPCONS (http://topcons.net/) [59], with default parameters.
(DOCX)

S4 Fig Secondary structures of ω-3 FAD proteins from chia and perilla. They were predicted by SOPMA [70]. Alfa-helix, extended strand, β-turn and random coils are shown with the longest, middle long, short and the shortest vertical bars, respectively.
(DOCX)
