# Supplementary material for: Omega-3 fatty acid desaturase gene family from two ω-3 sources, Salvia hispanica and Perilla frutescens: Cloning, characterization and expression
Source: PLoS One. 2018 Jan 19;13(1):e0191432. doi: 10.1371/journal.pone.0191432 (PMC5774782; doi:10.1371/journal.pone.0191432)
Supplement: S1 Table — a N: A or G or C or T; V: A or G or C; D: G or A or T. b Both degenerated bases and the restriction sites that were introduced are underlined, and Kozak sequences [80] are italicized in bold face. c T2A sequence [65] is wave-lined, and overlapped regions between two primers are in bold face. All primers were synthesized by Genscript (Nanjing, China) and Sangon Biotech (Shanghai, China). (DOCX) [file pone.0191432.s001.docx]

**S1 Table. Degenerate and non-degenerate used in this study**

| **Primer name** | **Sequence 5’-3’(the restriction sites)** |
| --- | --- |
| RPD155-1^a^ | TCAACATGNCCATGGTTCTGGTGGTG |
| RPD155-2 | CAATCATGNCCAAGAACAAAGAGAGCCCA |
| FPD153-1 | GGVACTCATGTGATACATCATCTCTTCCC |
| FPD153-2 | CATCATCTCTTCCCDCAGATCCCACA |
| FPfFAD3 | GTTCTAACTGAAACTCGCTAGTTTATTG |
| RPfFAD3a | AGAGGTCAACAAAATAACCCACAAAAAC |
| RPfFAD3b | CAAAGGTCAACAAAATAACCAAAAATAAG |
| FPfFAD7 | GGAGGCATAGGTGGTGGAGTC |
| RPfFAD7 | AGAGAATAATCTGGCTTTCCCTCTCA |
| FPfFAD8 | GGGAAAGAGAGAAGAAAGAGAGGCA |
| RPfFAD8 | GGAAGAACAAGAAGCCGTGGGGAA |
| FPfFAD3q | CCCACATTACCACTTAGTGGAG |
| RPfFAD3q | GGCAATCAAGAGTCCATCACTAAATC |
| FPfFAD7q | ACCACACTACCATTTGATAGAAGCA |
| RPfFAD7q | CTCGAGAACTCAATTAGAGACAGCT |
| FPfFAD8q | ACCACACTACCATTTGATAGAAGCT |
| RPfFAD8q | CACTACAGCCTAAGATTTGCGACCT |
| F25SRT | GATTTCTGCCCAGTGCTCTGAA |
| R25SRT | TCTGCCAAGCCCGTTCCCTT |
| FPfFAD3Y^b^ | GGATCC***AAAAAAATG***TCTGTTTCTTCCGGT (*Bam*HI) |
| RPfFAD3Y  FPfFAD7Y | TCTAGACTAAATCTTTTTGGAAGGAAAGAGC (*Xba*I)  GAGCTC***AAAAAAATG***TTTCAGGAGGTGGAGGAAGA (*Bam*HI) |
| RPfFAD7Y  FPfFAD8Y  RPfFAD8Y | CTCGAGATTCAGCTCAGGATCGGTCTG (*Xba*I)  GAGCTC***AAAAAAATG***ATTCAGCCAGTGGAAGAAGAG (*Bam*HI)  CTCGAGAGATTTGCGACCTCCATTGAG (*Xba*I) |
| FShFAD3-1 | CTTCAAACTGAAAACCAGCTCCTCGT |
| FShFAD3-2 | TCCTCCACAAATGTGAACACGTATATG |
| RShFAD3-1 | AACAAAATATACCAAACCTCTTCTTTTC |
| RShFAD3-2 | GATAATACAACAAGTAAACTTTATTTAAAGCAAC |
| FShFAD7 | AACTCTACACACATTCTCTCTCCCA |
| RShFAD7a | GAATAACTTAATGAATCACATTACTTGAATATAAT |
| RShFAD7b | GAATCACATAACCTGAATATAATTATTTCATAG |
| FShFAD8 | AGCCACAAATTGCTTCCACTTGCATACTTC |
| RShFAD8 | GCAATTTCAATCAAATATACAGTGTTCAATGCCA |
| FShFAD3-1q | ATCCCACACTACCATTTAGTGGAA |
| RShFAD3-1q | CTCTTTTGACGAAAATAGCTGACTATCA |
| FShFAD3-2q | ATCCCACATTACCATTTAGTGGAG |
| RShFAD3-2q | CTCTTTAGAAGAAAATAGTTCTCCATCT |
| FShFAD7q | CCACACTACCATTTGATAGAAGCA |
| RShFAD7q | GCTTAGGATCGGTCTGGTAGTACAC |
| FShFAD8q | CCACACTACAACCTAATAGAAGCT |
| RShFAD8q | CAGCTTAAGATTTTTGACCCCCAT |
| FShFAD3-1Y | GGATCC***AAAAAAATG***GCCGTTTCTTCCGGT (*Bam*HI) |
| RShFAD3-1Y | TCTAGACTAAATCTCTTTTGACGAAAATAGCTG (*Xba*I) |
| FShFAD3-2Y | GGATCC***AAAAAAATG***GCCGTCTCTTCCGGT (*Bam*HI) |
| RShFAD3-2Y | TCTAGACTAAATCTCTTTAGAAGAAAATAGTTC (*Xba*I) |
| FShFAD7Y  RShFAD7Y  FShFAD8Y  RShFAD8Y  FT2A^c^  FT2ABnFD2  RBnFD2 | GAGCTC***AAAAAAATG***TTTCAGGAGGTGGGGGAGGA (*Sac*I)  CTCGAGGTTCAGCTTAGGATCGGTCTG (*Xho*I)  GAGCTC***AAAAAAATG***ATTCAGCCACTGGAAGAAGAG (*Sac*I)  CTCGAGAGATTTTTGACCCCCATTGAG (*Xho*I)  CTCGAGAGAGCAGAAGGAAGGGGTTCTTT**GTTGACTTGTGGAGATGTTG** (*Xho*I)  **GTTGACTTGTGGAGATGTTG**AGGAGAATCCAGGACCAGCTACATACAAGGTCAAGTTC  TCTAGATTAAACAATGTCATCTTCTCTGTGG (*Xba*I) |

^a^ N: A or G or C or T; V: A or G or C; D: G or A or T. ^b^ Both degenerated bases and the restriction sites that were introduced are underlined, and Kozak sequences [80] are italicized in bold face. ^c^ T2A sequence [65] is wave-lined, and overlapped regions between two primers are in bold face. All primers were synthesized by Genscript (Nanjing, China) and Sangon Biotech (Shanghai, China).
