# Supplementary material for: Omega-3 fatty acid desaturase gene family from two ω-3 sources, Salvia hispanica and Perilla frutescens: Cloning, characterization and expression
Source: PLoS One. 2018 Jan 19;13(1):e0191432. doi: 10.1371/journal.pone.0191432 (PMC5774782; doi:10.1371/journal.pone.0191432)
Supplement: S2 Table — (DOCX) [file pone.0191432.s002.docx]

**S2 Table. Basicparameters of the *ω-3 FAD* genes from chia and perilla**

| Gene name | Genomic sequence (bp) | Intron length (upper) and position (lower) | | | | | | |  | G+C content (%) | | | |
| --- | --- | --- | --- | --- | --- | --- | --- | --- | --- | --- | --- | --- | --- |
|  |  | Intron 1 | Intron 2 | Intron 3 | Intron 4 | Intron 5 | Intron 6 | Intron 7 |  | ORF | 5’UTR | Intron | 3’UTR |
| *PfFAD3a* | 2,824 | 237 | 83 | 121 | 126 | 150 | 375 | 287 |  | 48.47 | 38.78 | 22.89-38.67 | 30.00 |
|  |  | 388-624 | 715-797 | 865-985 | 1,079-1,204 | 1,391-1,540 | 1,622-1,996 | 2,135-2,421 |  |  |  |  |  |
| *PfFAD7a* | 2,646 | 123 | 93 | 113 | 100 | 112 | 151 | 88 |  | 44.65 | 39.29 | 23.86-36.00 | 34.02 |
|  |  | 803-925 | 1,016-1,108 | 1,176-1,288 | 1,382-1,481 | 1,668-1,779 | 1,861-2,011 | 2,150-2,237 |  |  |  |  |  |
| *PfFAD8a* | 2,849 | 90 | 136 | 91 | 104 | 188 | 187 | 96 |  | 47.23 | 46.32 | 28.34-36.46 | 35.00 |
|  |  | 860-949 | 1,040-1,175 | 1,243-1,333 | 1,427-1,530 | 1,717-1,904 | 1,986-2,172 | 2,311-2,406 |  |  |  |  |  |
| *ShFAD3-1* | 3,691 | 372 | 329 | 77 | 82 | 209 | 719 | 403 |  | 50.51 | 47.06 | 22.89-38.67 | 28.37 |
|  |  | 379-752 | 843-1,171 | 1,239-1,315 | 1,409-1,490 | 1,677-1,885 | 1,967-2,685 | 2,824-3,226 |  |  |  |  |  |
| *ShFAD3-2* | 2,563 | 59 | 74 | 78 | 103 | 99 | 503 | 152 |  | 50.69 | 43.20 | 20.20-29.82 | 30.28 |
|  |  | 440-498 | 589-662 | 730-807 | 901-1,003 | 1,190-1,288 | 1,370-1,872 | 2,011-2,162 |  |  |  |  |  |
| *ShFAD7a* | 2,678 | 223 | 112 | 111 | 103 | 97 | 90 | 92 |  | 47.47 | 40.59 | 22.32-39.18 | 35.38 |
|  |  | 703-925 | 1,016-1,127 | 1,195-1,305 | 1,399-1,501 | 1,688-1,784 | 1,866-1,955 | 2,094-2,185 |  |  |  |  |  |
| *ShFAD7b* | 2,655 | 226 | 109 | 111 | 103 | 96 | 90 | 95 |  | 47.77 | 39.60 | 25.69-40.62 | 36.00 |
|  |  | 703-928 | 1,019-1,127 | 1,195-1,305 | 1,399-1,501 | 1,688-1,783 | 1,865-1,954 | 2,093-2,187 |  |  |  |  |  |
| *ShFAD8* | 2,717 | 91 | 99 | 95 | 79 | 147 | 310 | 102 |  | 47.05 | 44.44 | 29.29-39.24 | 27.95 |
|  |  | 660-750 | 841-939 | 1,007-1,101 | 1,195-1,273 | 1,460-1,606 | 1,688-1,997 | 2,136-2,237 |  |  |  |  |  |
