# Supplementary material for: Omega-3 fatty acid desaturase gene family from two ω-3 sources, Salvia hispanica and Perilla frutescens: Cloning, characterization and expression
Source: PLoS One. 2018 Jan 19;13(1):e0191432. doi: 10.1371/journal.pone.0191432 (PMC5774782; doi:10.1371/journal.pone.0191432)
Supplement: S3 Table — * Pairwise-alignment and identity analysis of mRNAs and proteins of ω-3 FADs from perilla, chia and Arabidopsis were performed using the ClustalW method in Vector NTI advance 11.5.1 (Invitrogen, USA). (DOCX) [file pone.0191432.s003.docx]

**S3 Table. Identities of mRNAs (Italic) and proteins among the ω-3 FADs from chia, perilla and Arabidopsis^*^**

|  | AtFAD3 (%) | AtFAD7 (%) | AtFAD8 (%) | PfFAD3a (%) | PfFAD3b (%) | PfFAD7a (%) | PfFAD7b (%) | PfFAD8a (%) | PfFAD8b (%) | ShFAD3-1 (%) | ShFAD3-2 (%) | ShFAD7a (%) | ShFAD7b (%) | ShFAD8 (%) |
| --- | --- | --- | --- | --- | --- | --- | --- | --- | --- | --- | --- | --- | --- | --- |
| AtFAD3 |  | *62.3* | *63.3* | *61.8* | *62.2* | *61.5* | *61.5* | *61.0* | *46.2* | *62.6* | *62.0* | *61.2* | *61.6* | *61.6* |
| AtFAD7 | 56.3 |  | *75.1* | *61.9* | *62.7* | *64.7* | *65.1* | *65.0* | *56.5* | *60.6* | *59.7* | *63.3* | *63.1* | *64.6* |
| AtFAD8 | 58.4 | 77.6 |  | *63.2* | *63.3* | *65.4* | *65.4* | *66.9* | *58.4* | *61.8* | *61.0* | *65.2* | *64.9* | *65.9* |
| PfFAD3a | 66.4 | 57.4 | 57.6 |  | *98.0* | *61.4* | *61.4* | *62.7* | *45.6* | *80.3* | *78.4* | *60.0* | *60.7* | *62.6* |
| PfFAD3b | 66.2 | 57.6 | 57.8 | 99.7 |  | *61.3* | *61.3* | *63.3* | *45.8* | *80.2* | *78.8* | *60.3* | *61.1* | *61.7* |
| PfFAD7a | 57.6 | 71.7 | 73.3 | 59.1 | 59.1 |  | *99.8* | *68.7* | *63.1* | *59.8* | *59.5* | *79.8* | *80.1* | *68.9* |
| PfFAD7b | 57.6 | 71.7 | 73.3 | 59.1 | 59.1 | 100.0 |  | *68.7* | *63.2* | *59.7* | *59.4* | *79.8* | *80.0* | *68.8* |
| PfFAD8a | 59.6 | 71.5 | 73.9 | 60.3 | 60.3 | 81.3 | 81.3 |  | *95.7* | *62.2* | *60.6* | *68.6* | *68.6* | *78.4* |
| PfFAD8b | 47.2 | 55.5 | 57.1 | 47.2 | 47.2 | 71.3 | 71.3 | 98.7 |  | *46.1* | *45.7* | *60.6* | *60.6* | *73.3* |
| ShFAD3-1 | 66.1 | 56.1 | 56.4 | 88.5 | 88.5 | 60.0 | 60.0 | 60.3 | 46.3 |  | *86.3* | *60.7* | *61.1* | *61.7* |
| ShFAD3-2 | 66.2 | 54.8 | 55.3 | 85.4 | 85.4 | 58.2 | 58.2 | 58.9 | 51.3 | 90.3 |  | *59.9* | *59.7* | *59.8* |
| ShFAD7a | 57.3 | 72.7 | 74.8 | 59.3 | 59.6 | 87.8 | 87.8 | 79.4 | 63.1 | 59.7 | 58.0 |  | *96.4* | *67.7* |
| ShFAD7b | 57.3 | 72.7 | 74.8 | 59.3 | 59.6 | 87.8 | 87.8 | 79.4 | 63.1 | 59.7 | 58.0 | 100.0 |  | *67.5* |
| ShFAD8 | 58.8 | 72.6 | 73.4 | 60.4 | 60.4 | 78.8 | 78.8 | 87.9 | 78.8 | 60.8 | 58.5 | 78.7 | 78.7 |  |

^*^Pairwise-alignment and identity analysis of mRNAs and proteins of ω-3 FADs from perilla, chia and Arabidopsis were performed using ClustalW method in Vector NTI advance 11.5.1 (Invitrogen, USA).
