# Supplementary material for: Omega-3 fatty acid desaturase gene family from two ω-3 sources, Salvia hispanica and Perilla frutescens: Cloning, characterization and expression
Source: PLoS One. 2018 Jan 19;13(1):e0191432. doi: 10.1371/journal.pone.0191432 (PMC5774782; doi:10.1371/journal.pone.0191432)
Supplement: S1 Fig — The start codon (ATG) and the stop codon (TAA, TAG and TGA) are in underlined in bold face. Alternative transcription sites and poly(A) tailing sites are underlined and italicized, and the major types are shown in bold face. The introns and typical and non-typical poly(A) signals are underlined. The purine-stretches (> 20 bp) and pyrimidine-stretches (> 20 bp) are highlighted by the gray background. (DOCX) [file pone.0191432.s005.docx]

                                                  M  A  V  S  S  G  A  R  L  S  K  S  G  A  D  G  E

       1 *G*TTCTAACTGAAACTCGCTAGTTTATTGATTCAAACCCTCCCCAAAAGA**ATG**GCCGTTTCTTCCGGTGCCCGCCTCTCGAAGAGTGGCGCTGATGGAGAG

 V  F  D  G  Q  Q  Q  Y  E  G  I  G  K  R  A  A  D  K  F  D  P  A  A  P  P  P  F  K  I  A  D  I  R

     101 GTTTTCGACGGCCAACAACAATACGAAGGAATCGGAAAGCGTGCGGCCGACAAATTCGACCCCGCCGCGCCGCCGCCCTTCAAGATCGCCGACATCCGAG

A  A  I  P  A  H  C  W  V  K  S  P  W  R  S  L  S  Y  V  V  W  D  V  A  A  V  F  A  L  L  A  A  A  V

     201 CGGCCATACCGGCGCATTGCTGGGTGAAGAGTCCGTGGCGCTCATTGAGCTACGTCGTTTGGGACGTCGCCGCCGTATTCGCGCTGCTCGCCGCCGCCGT

  Y  I  N  S  W  A  F  W  P  V  Y  W  I  A  Q  G  T  M  F  W  A  L  F  V  L  G  H  D  C

     301 TTACATCAACAGCTGGGCGTTTTGGCCGGTTTACTGGATTGCTCAGGGCACCATGTTTTGGGCCCTTTTCGTTCTCGGGCATGATTGGTAATTCCTTTAA

     401 TGCCATATTTTTATAAAATGTACCATCTTCCGTTTATTTATTTAATTGTAAGATATGTATATAATGTTCTATTATAGTTCAATCTCGTATTTTATATCGG

     501 ATTAGTTTTTTTTTAATGCTCATTGTACCTACATTGATAAATCTAGCGCTAGTGGTGTAGTGCGGGTATCGTGGTAGAAAATAGTGTTTTAATTTAGTGA

                          G  H  G  S  F  S  D  N  T  T  L  N  N  V  V  G  H  V  L  H  S  S  I  L  V

     601 AAATCTAATATTGGTTAATTGCAGTGGGCACGGGAGTTTTTCAGACAATACCACGCTGAATAACGTGGTGGGACATGTGCTTCACTCATCAATTCTTGTA

 P  Y  H  G  W

                                                                                                   R

     701 CCGTATCATGGATGGTTAGTTCTTGGAAATAAATCTGAGTTCATTTTAATAATTTTGTGCGATTATTTTCAAATATTTCAATTACATTTTACTACAGGCG

  I  S  H  R  T  H  H  Q  N  H  G  H  V  E  K  D  E  S  W  V  P

     801 AATCAGCCATAGAACTCACCACCAAAACCATGGACATGTCGAGAAGGACGAGTCGTGGGTGCCGGTAACGTTTTATTTTTTATTTTTATTTTCGTTCTAA

                                                                                      L  P  E  N  L

     901 ATGAGATACGTAATTCATCCTGCATTATTGAATTTTGTGTTTCGATATTAATCACCTCTATTTTATTCCCTAAATATTATTGCAGTTGCCTGAGAATTTG

 Y  K  K  L  D  F  S  T  K  F  L  R  Y  K  I  P  F  P  M  F  A  Y  P  L  Y  L

    1001 TACAAGAAGTTGGATTTTTCCACCAAATTCTTGAGATACAAAATCCCATTCCCCATGTTTGCATATCCTTTATATTTGGTAATCTCCCTCCCTCCCTCTC

    1101 TCTCTCTCTATATATATATATATAGAAATAAAATTGAGATTTACAACTGATTGTATTGTTTATGTGTTTTTTTTATAAATAAAAAAATTTGTTTATTGTT

     W  Y  R  S  P  G  K  T  G  S  H  F  N  P  Y  S  D  L  F  K  P  N  E  R  G  L  I  V  T  S  T  M

    1201 TCAGTGGTATAGAAGTCCGGGAAAAACTGGATCTCACTTCAACCCTTACAGCGATTTGTTTAAACCAAATGAGAGGGGCTTGATAGTGACTTCAACAATG

 C  W  A  A  M  G  V  F  L  L  Y  A  S  T  I  V  G  P  N  M  M  F  K  L  Y  G  V  P  Y  L

    1301 TGCTGGGCTGCAATGGGTGTTTTCCTCCTCTATGCCTCCACCATTGTTGGTCCAAACATGATGTTCAAGCTCTACGGCGTACCATATTTGGTAACGAACA

    1401 TGAAACACAATATGATACGATACTTTGATGTTCGAGTTCAGAGATCTCTGAACTCGAATCCCACCAAACCACCACATCACATTAATTTATCGTTGTTCTG

                                         I  F  V  M  W  L  D  T  V  T  Y  L  H  H  H  G  Y  D  K  K

    1501 AGTACTGTCTGACGAGATTTCTTTTTATTTTGTCGGGCAGATATTCGTGATGTGGTTGGACACGGTAACATACTTACACCACCACGGTTATGACAAGAAA

 L  P  W  Y  R  S  K

    1601 CTCCCTTGGTACCGCAGCAAGGTAAAATTTCATTACTATTTTTTTTTACTTATGCCTCTTACCATATATAAAATTATATATATTCTTCAAGATTGATGCA

    1701 GACTATTAAATTTGAGACAAATATATGTACGTGCAATCCGAGATATTTGTTGAGTTGTAACGGTCACCTACCGCATTAGCATGTGTCACTATTTGACAAT

    1801 ATAACAGTATCATATATAAACTAATATGTGGCTGGAATTCATTTTTTTCTTCACCTGCAACGCCTTATAGATGGAGTTTGTGACACAATGTTTTCCTAAT

                                                                                                 E

    1901 GTAGACGAGGACAGCTGATGGTGCACTGTAAAGCACACTAACGAATATGTGGCCTTAAAAGCAGTAGTTGATGTTGTTTTTGTGTATAATTTGCAGGAAT

W  S  Y  L  R  G  G  L  T  T  V  D  Q  D  Y  G  F  F  N  K  I  H  H  D  I  G  T  H  V  I  H  H  L  F

    2001 GGAGTTATTTACGAGGAGGATTGACGACCGTAGATCAAGATTATGGATTTTTTAATAAAATTCACCACGATATTGGCACCCATGTTATACACCATCTATT

  P  Q  I  P  H  Y  H  L  V  E  A

    2101 CCCTCAGATCCCACATTACCACTTAGTGGAGGCGGTGAGATCTCTCTCTAAACCACATTATTTATAAACCCTAATCACGTGATCATCGCAATTTAATATT

    2201 ACTTCTAATTTAATTAGCTGTAGCACCTCTTACTCTGTCACTATTTATAGGAACTGTCATTTGAGATTCAAATTTTAAATAAGACTAAAATCTTGGTCTA

    2301 GTTTGTACTAATAGTAATTTGTTTTACGTAGATTCTCTTCTTTCAGGTGTGCATATTTTTTATTATTGATAGTGAGATAATAAAATACGAGATTAAAACA

                      T  R  E  A  K  R  V  L  G  N  Y  Y  R  E  P  R  K  S  G  P  V  P  L  H  L  I

    2401 CGAGTTTGGCTGTATTTGCAGACAAGGGAGGCGAAAAGGGTGCTGGGGAATTACTACAGGGAGCCCAGAAAATCTGGGCCAGTTCCACTGCACTTAATTC

P  A  L  L  K  S  L  G  R  D  H  Y  V  S  D  N  G  D  I  V  Y  Y  Q  T  D  D  E  L  F  P  S  K  K  I

    2501 CTGCCTTGTTGAAAAGTCTTGGTAGAGATCATTATGTTAGTGATAATGGAGACATAGTTTATTATCAAACAGATGATGAGCTCTTTCCTTCCAAAAAGAT

    2601 T**TAG**TGATGGACTCTTGATTGCCAAATTAGATTTAATTTACAGTAGTCCTTTGTGCCACAATATTTTGTTTAGGCCAGGAAATATTGTGTGCACAAATTA

    2701 AATAACTCTAGTGAGTTTTTTTTTTTGGATCAAGTGTTTGTTACCTTTTCTTTTTCCTGTGATAAATGTAATGTTCC*TT*AAATAAACTTATTTTTGGTTT

    2801 TTGT*G*GGTTATTTTGTTGACCTC*T*

A: *PfFAD3a* gene

                                                  M  A  V  S  S  G  A  R  L  S  K  S  G  A  D  G  E

       1 *G*TTCTAACTGAAACTCGCTAGTTTATTGATTCAAACCCTCCCCAAAAGA**ATG**GCCGTTTCTTCCGGTGCCCGCCTCTCGAAGAGTGGCGCTGATGGAGAG

 V  F  D  G  Q  Q  Q  Y  E  G  I  G  K  R  A  A  D  K  F  D  P  A  A  P  P  P  F  K  I  A  D  I  R   ·

     101 GTTTTCGACGGCCAACAACAATACGAAGGAATCGGAAAGCGTGCGGCCGACAAATTCGACCCCGCCGCGCCGCCGCCCTTCAAGATCGCCGACATCCGAG

A  A  I  P  A  H  C  W  V  K  S  P  W  R  S  L  S  Y  V  V  W  D  V  A  A  V  F  A  L  L  A  A  A  V ·

     201 CGGCCATACCGGCGCATTGCTGGGTGAAGAGTCCGTGGCGCTCATTGAGCTACGTCGTTTGGGACGTCGCCGCCGTATTCGCGCTGCTCGCCGCCGCCGT

  Y  I  N  S  W  A  F  W  P  V  Y  W  I  A  Q  G  T  M  F  W  A  L  F  V  L  G  H  D  C  G  H  G  S

     301 TTACATCAACAGCTGGGCGTTTTGGCCGGTTTACTGGATTGCTCAGGGCACCATGTTTTGGGCCCTTTTCGTTCTCGGGCATGATTGTGGGCACGGGAGT

 F  S  D  N  T  T  L  N  N  V  V  G  H  V  L  H  S  S  I  L  V  P  Y  H  G  W  R  I  S  H  R  T  H   ·

     401 TTTTCAGACAATACCACGCTGAATAACGTGGTGGGACATGTGCTTCACTCATCAATTCTTGTACCGTATCATGGATGGCGAATCAGCCATAGAACTCACC

H  Q  N  H  G  H  V  E  K  D  E  S  W  V  P  L  P  E  N  L  Y  K  K  L  D  F  S  T  K  F  L  R  Y  K ·

     501 ACCAAAACCATGGACATGTCGAGAAGGACGAGTCGTGGGTGCCGTTGCCTGAGAATTTGTACAAGAAGTTGGATTTTTCCACCAAATTCTTGAGATACAA

  I  P  F  P  M  F  A  Y  P  L  Y  L  W  Y  R  S  P  G  K  T  G  S  H  F  N  P  Y  S  D  L  F  K  P

     601 AATCCCATTCCCCATGTTTGCATATCCTTTATATTTGTGGTATAGAAGTCCGGGAAAAACTGGATCTCACTTCAACCCTTACAGCGATTTGTTTAAACCA

 N  E  R  G  L  I  V  T  S  T  M  C  W  A  A  M  G  V  F  L  L  Y  A  S  T  I  V  G  P  N  M  M  F   ·

     701 AATGAGAGGGGCTTGATAGTGACTTCAACAATGTGCTGGGCTGCAATGGGTGTTTTCCTCCTCTATGCCTCCACCATTGTTGGTCCAAACATGATGTTCA

K  L  Y  G  V  P  Y  L  I  F  V  M  W  L  D  T  V  T  Y  L  H  H  H  G  Y  D  K  K  L  P  W  Y  R  S ·

     801 AGCTCTACGGCGTACCATATTTGATATTCGTGATGTGGTTGGACACGGTAACATACTTACACCACCACGGTTATGACAAGAAACTCCCTTGGTACCGCAG

  K  E  W  S  Y  L  R  G  G  L  T  T  V  D  Q  D  Y  G  F  F  N  K  I  H  H  D  I  G  T  H  V  I  H

     901 CAAGGAATGGAGTTATTTACGAGGAGGATTGACGACCGTAGATCAAGATTATGGATTTTTTAATAAAATTCACCACGATATTGGCACCCATGTTATACAC

 H  L  F  P  Q  I  P  H  Y  H  L  V  E  A  T  R  E  A  K  R  V  L  G  N  Y  Y  R  E  P  R  K  S  G   ·

    1001 CATCTATTCCCTCAGATCCCACATTACCACTTAGTGGAGGCGACAAGGGAGGCGAAAAGGGTGCTGGGGAATTACTACAGGGAGCCCAGAAAATCTGGGC

P  V  P  L  H  L  I  P  A  L  L  K  S  L  G  R  D  H  Y  V  S  D  N  G  D  I  V  Y  Y  Q  T  D  D  E ·

    1101 CAGTTCCACTGCACTTAATTCCTGCCTTGTTGAAAAGTCTTGGTAGAGATCATTATGTTAGTGATAATGGAGACATAGTTTATTATCAAACAGATGATGA

  L  F  P  S  K  K  I  *

    1201 GCTCTTTCCTTCCAAAAAGATT**TAG**TGATGGACTCTTGATTGCCAAATTAGATTTAATTTACAGTAGTCCTTTGTGCCACAATATTTTGTTTAGGCCAGG

    1301 AAATATTGTGTGCACAAATTAAATAACTCTAGTGAGTTTTTTTTTTTGGATCAAGTGTTTGTTACCTTTTCTTTTTCCTGTGATAAATGTAATGTTCC*TT*

    1401 AAATAAACTTATTTTTGGTTTTTGT*G*GGTTATTTTGTTGACCTC*TAAAAAAAAAAAAAAAAAAAAAAAAAAAAAAA*

B: *PfFAD3a*mRNA

                                                  M  A  V  S  S  G  A  R  L  S  K  S  G  A  D  G  E

       1 ***G***TTCT***A***ACT***G***AAACTCGCTAGTTTATT*G*ATTCAAACCCTCCCCAAAAGA**ATG**GCCGTTTCTTCCGGTGCCCGCCTCTCGAAGAGTGGCGCTGATGGAGAG

 V  F  D  G  Q  Q  Q  Y  E  G  I  G  K  R  A  A  D  K  F  D  P  A  A  P  P  P  F  K  I  A  D  I  R   ·

     101 GTTTTCGACGGCCAACAACAATACGAAGGAATCGGAAAACGTGCGGCCGACAAATTCGACCCCGCCGCGCCGCCGCCGTTCAAGATCGCCGACATCCGAG

A  A  I  P  A  H  C  W  V  K  N  P  W  R  S  L  S  Y  V  V  W  D  V  A  A  V  F  A  L  L  A  A  A  V ·

     201 CGGCCATACCGGCGCATTGCTGGGTGAAGAATCCGTGGCGCTCATTGAGCTACGTCGTTTGGGACGTCGCCGCCGTCTTCGCGCTGCTCGCCGCCGCCGT

  Y  I  N  S  W  A  F  W  P  V  Y  W  I  A  Q  G  T  M  F  W  A  L  F  V  L  G  H  D  C  G  H  G  S

     301 TTACATCAACAGCTGGGCGTTTTGGCCGGTTTACTGGATTGCTCAGGGAACCATGTTTTGGGCCCTTTTCGTTCTTGGGCATGATTGTGGGCACGGGAGT

 F  S  D  N  T  T  L  N  N  V  V  G  H  V  L  H  S  S  I  L  V  P  Y  H  G  W  R  I  S  H  R  T  H   ·

     401 TTTTCGGACAATACCACGCTGAATAACGTGGTGGGACATGTGCTTCACTCATCAATTCTTGTACCGTATCATGGATGGCGAATCAGCCATAGAACTCACC

H  Q  N  H  G  H  V  E  K  D  E  S  W  V  P  L  P  E  N  L  Y  K  K  L  D  F  S  T  K  F  L  R  Y  K ·

     501 ACCAAAACCATGGACATGTCGAGAAGGACGAGTCGTGGGTGCCGTTGCCTGAGAATTTGTACAAGAAGTTGGATTTTTCCACCAAATTCTTGAGATACAA

  I  P  F  P  M  F  A  Y  P  L  Y  L  W  Y  R  S  P  G  K  T  G  S  H  F  N  P  Y  S  D  L  F  K  P

     601 AATCCCATTCCCCATGTTTGCATACCCTTTATATTTGTGGTATAGAAGTCCGGGAAAAACTGGATCTCACTTCAACCCTTACAGCGATTTGTTTAAACCA

 N  E  R  G  L  I  V  T  S  T  M  C  W  A  A  M  G  V  F  L  L  Y  A  S  T  I  V  G  P  N  M  M  F   ·

     701 AATGAGAGGGGTTTGATAGTGACTTCAACAATGTGCTGGGCTGCAATGGGTGTTTTCCTCCTCTATGCCTCCACCATTGTTGGTCCAAACATGATGTTCA

K  L  Y  G  V  P  Y  L  I  F  V  M  W  L  D  T  V  T  Y  L  H  H  H  G  Y  D  K  K  L  P  W  Y  R  S ·

     801 AGCTCTACGGCGTACCGTATTTGATATTCGTGATGTGGTTGGACACGGTAACATACTTACACCACCACGGTTATGACAAGAAACTCCCTTGGTACCGCAG

  K  E  W  S  Y  L  R  G  G  L  T  T  V  D  Q  D  Y  G  F  F  N  K  I  H  H  D  I  G  T  H  V  I  H

     901 CAAGGAATGGAGTTATTTACGAGGAGGATTGACGACCGTAGATCAAGATTATGGATTTTTTAATAAAATTCACCACGATATTGGCACCCATGTTATACAC

 H  L  F  P  Q  I  P  H  Y  H  L  V  E  A  T  R  E  A  K  R  V  L  G  N  Y  Y  R  E  P  R  K  S  G   ·

    1001 CATCTATTCCCTCAGATCCCACATTACCACTTAGTGGAGGCGACAAGGGAGGCGAAAAGGGTGCTGGGGAATTACTACAGGGAGCCCAGAAAATCTGGGC

P  V  P  L  H  L  I  P  A  L  L  K  S  L  G  R  D  H  Y  V  S  D  N  G  D  I  V  Y  Y  Q  T  D  D  E ·

    1101 CAGTTCCACTACACTTAATTCCTGCCTTGTTGAAAAGTCTTGGTAGAGATCATTATGTTAGTGATAATGGAGACATAGTTTATTATCAAACAGATGATGA

  L  F  P  S  K  K  I  *

    1201 GCTCTTTCCTTCCAAAAAGATT**TAG**TGATGGACTCTTGATTGCCAAATTAGATTTAATTTACAGTAGTCCTTTGTGCCACAATATTTTGTTTAGGCCAGG

    1301 AAATATTGTGTGCACAAATTAAATAACTCTAGTGAGTTTTTTTTGGATCAAGTGTTTGTTACCTTTTTTTTTTTTCCTGTGATAAATGTAATGTTCCTTA

    1401 AATAAACTTATTTTTGG*TT*ATTTTGT***T***GACC*T*TT***G****AAAAAAAAAAAAAAAAAAAAAAAAAAAAAA*

C: *PfFAD3b*mRNA

       1 ***G***G*A*GGCATAGGTGGTGGAGTCGGAAAGAGAGAAGAAATAGAGGCTGAAAATTGTGTATTTAAATGAATCACATTCTGCTCGGGTTATAA***G***CCTCAAGAAA

     101 ATCCAAATCAACAAGTT*G*GGATTCTTGGAAGCCATAAATTTGAGGAGAGAATCCCTTGTATTATCTACTAACTTAAGCATATACATACATATTCTGCAAA

     201 CCCCTCAAGTCCACACACTATTCTCTCTCTTTCTCTCTCATTCTAGTGAGAGAACCCCAATAAAGGTGAAATCTTGATTACTTTCAGAGTT*G*GGGCTCTC

         M  A  S  W  V  L  S  E  C  G  L  R  P  L  P  R  I  Y  P  K  P  R  T  G  Q  Y  L  S  N  S  N ·

     301 TCTCTGAA**ATG**GCGAGTTGGGTGTTATCAGAATGTGGTTTAAGGCCACTTCCAAGAATATACCCCAAGCCAAGAACTGGCCAGTATCTCTCCAATTCCAA

  P  S  K  L  R  L  S  R  T  G  F  S  S  D  S  S  F  S  L  V  G  R  E  R  N  W  G  L  K  V  S  A  P

     401 CCCCTCAAAGTTGAGACTCTCAAGAACAGGTTTTTCAAGTGATTCCTCATTCAGTTTGGTTGGTAGAGAGAGAAACTGGGGTTTGAAGGTGAGTGCTCCA

 L  R  F  Q  E  V  E  E  E  S  E  E  R  G  S  V  I  V  N  G  V  D  E  F  D  P  G  A  P  P  P  F  K   ·

     501 CTGAGATTTCAGGAGGTGGAGGAAGAGAGTGAAGAGAGAGGGAGTGTAATAGTAAATGGTGTTGATGAATTCGACCCTGGTGCACCACCACCATTCAAGC

L  S  D  I  R  A  A  I  P  K  H  C  W  V  K  D  P  W  R  S  M  S  Y  V  V  R  D  V  V  V  V  F  G  L ·

     601 TGTCTGATATTCGGGCAGCCATTCCTAAACATTGTTGGGTTAAGGATCCATGGAGGTCTATGAGCTATGTTGTAAGAGATGTTGTTGTTGTTTTTGGATT

  A  A  A  A  A  Y  F  N  N  W  A  V  W  P  I  Y  W  F  A  Q  S  T  M  F  W  A  L  F  V  L  G  H  D

     701 GGCTGCCGCTGCAGCCTATTTCAACAACTGGGCTGTTTGGCCTATCTACTGGTTCGCTCAGTCAACTATGTTTTGGGCTCTGTTTGTTCTTGGACATGAT

 C

     801 TGGTAAATTTTTCTTTTTTCCTTTTTTTTTCGTTTTTTTTTTTTTGTGGATTCTGATTCTGGTTTGTGTTTGTATTAGTACATTTAGAGGTGTAGTTTTT

                           G  H  G  S  F  S  N  D  P  K  L  N  S  V  A  G  H  L  L  H  S  S  I  L  V ·

     901 TTTAATGATTCTTGAATTGTTGCAGTGGACATGGGAGCTTTTCTAATGATCCCAAGTTGAATAGTGTTGCTGGTCACCTGCTTCACTCTTCCATTCTTGT

  P  Y  H  G  W

    1001 TCCTTATCATGGATGGTATGTTTGAATCTCAAAATCATAATTTATGTTTGTGTGTGTGTGTGTGAACCAAAATTGATTTAACAAACTGTGTAATGGTGAA

          R  I  S  H  R  T  H  H  Q  N  H  G  H  V  E  N  D  E  S  W  H  P

    1101 TGATTTAGGAGAATTAGTCACAGGACACACCATCAGAACCATGGACATGTTGAAAATGATGAATCATGGCATCCAGTATGTTGTTTCTACCTTTGTTATG

                                                                                         I  P  E  K

    1201 TGGAAATTTAGTTGCATTCTTCCTTTTTTTCCCTCGAAAAATGAAAGTTTAGGTGTTTGTTTAATTTATATCACATTCTTTGTTCCAGATACCTGAGAAG

 I  Y  R  T  L  D  F  A  T  K  K  L  R  F  T  L  P  F  P  M  L  A  Y  P  F  Y  L

    1301 ATATACAGGACTTTGGATTTTGCCACCAAGAAGTTGAGGTTCACTTTGCCTTTCCCCATGCTGGCTTATCCCTTCTATCTGGTAGCCTACTTCCTTCAAA

                                                                                  W  G  R  S  P  G   ·

    1401 ATTAGAATTTTCACTGAAATTTTCTCATAGTGGTCTCTCCTGATCAATGCTGTTGATCCTGTATGATTCATTTCATGACAGTGGGGAAGAAGTCCTGGCA

K  K  G  S  H  F  H  P  D  S  D  L  F  V  P  N  E  R  K  D  V  I  T  S  T  V  C  W  T  A  M  V  A  I ·

    1501 AGAAAGGCTCTCATTTCCATCCAGACAGTGATTTGTTCGTTCCAAACGAGAGGAAAGATGTTATCACCTCAACTGTTTGTTGGACAGCAATGGTTGCAAT

  L  A  G  L  S  F  V  M  G  P  V  Q  L  L  K  L  Y  G  I  P  Y  I

    1601 ACTTGCAGGACTATCTTTTGTTATGGGTCCTGTTCAGTTGCTTAAACTCTATGGCATACCTTATATTGTTAGTTCCTTCTACTCCTTTAGATTGTCTCAC

                                                                                G  F  V  A  W  L  D

    1701 TTTTTTCATACATATATTGTTTAGCTTGTCTGAGTTCCATGCTCCATTGTATAACTTGAAAATGGAATTGGGCTTGCAGGGATTTGTGGCATGGCTTGAT

 L  V  T  Y  L  H  H  H  G  H  D  E  K  L  P  W  Y  R  G  K

    1801 TTAGTTACCTACTTACACCACCATGGCCACGATGAGAAGCTTCCTTGGTACCGAGGAAAGGTAAAATCAAACCTCAACATTGACATTAACTCTACTTAAA

    1901 GACCTGTAACTCCAGAAAGGATGAGTTAACAGCAAGAGACATATGGAAGAATGTTGAAGTTTTGTTGTTTCAATGTTCTTGCTGGCTCGTCTAAATTCGT

            E  W  S  Y  L  R  G  G  L  T  T  L  D  R  D  Y  G  W  I  N  N  I  H  H  D  I  G  T  H  V ·

    2001 TGAAAATGCAGGAATGGAGTTACCTGAGAGGGGGGCTCACGACACTTGATCGCGATTATGGATGGATAAACAACATCCACCATGACATAGGGACGCATGT

  I  H  H  L  F  P  Q  I  P  H  Y  H  L  I  E  A

    2101 TATACATCACCTCTTCCCACAAATACCACACTACCATTTGATAGAAGCAGTAAATATCAAATCTCACTAGCTTTTGGAAAACTATTTGATATTAGTTACA

                                      T  A  A  A  K  P  V  L  G  K  Y  Y  K  E  P  K  K  S  G  P  F

    2201 GATTATTTACTGATAATTGCTAATTATTTATATGCAGACTGCAGCAGCTAAGCCAGTTCTAGGAAAATATTACAAGGAGCCTAAGAAATCAGGCCCCTTT

 P  F  Y  L  L  G  V  L  Q  K  S  M  K  K  D  H  Y  V  S  D  T  G  D  I  V  Y  Y  Q  T  D  P  E  L   ·

    2301 CCATTCTACTTGTTGGGAGTCCTCCAAAAAAGCATGAAAAAGGATCACTATGTGAGTGACACGGGCGATATCGTTTACTACCAGACCGATCCTGAGCTGA

N  *

    2401 AT**TGA**ACTCAAGAATGATAAGAGTTTGAATGTTGTTATCAGTATATGTAAAAGCTGTCTCTAATTGAGTTCTCGAGACCTCTAAGGATCGCGTTCAGCTG

    2501 CAGAATTGATATATATATTTTTTTCTCTTCAACGATGGAGAGGCGATACTAGAGATTACAGAATATTGATGTGTATTTGTATGAGGATGTACTGATGCTA

    2601 TAATGTATCTTGATAATTAATGAGAGGGAAAGCCAGATTATTCTC*T*

D: *PfFAD7a*gene

       1 ***G***G*A*GGCATAGGTGGTGGAGTCGGAAAGAGAGAAGAAATAGAGGCTGAAAATTGTGTATTTAAATGAATCACATTCTGCTCGGGTTATAA***G***CCTCAAGAAA

     101 ATCCAAATCAACAAGTT*G*GGATTCTTGGAAGCCATAAATTTGAGGAGAGAATCCCTTGTATTATCTACTAACTTAAGCATATACATACATATTCTGCAAA

     201 CCCCTCAAGTCCACACACTATTCTCTCTCTTTCTCTCTCATTCTAGTGAGAGAACCCCAATAAAGGTGAAATCTTGATTACTTTCAGAGTT*G*GGGCTCTC

         M  A  S  W  V  L  S  E  C  G  L  R  P  L  P  R  I  Y  P  K  P  R  T  G  Q  Y  L  S  N  S  N ·

     301 TCTCTGAA**ATG**GCGAGTTGGGTGTTATCAGAATGTGGTTTAAGGCCACTTCCAAGAATATACCCCAAGCCAAGAACTGGCCAGTATCTCTCCAATTCCAA

  P  S  K  L  R  L  S  R  T  G  F  S  S  D  S  S  F  S  L  V  G  R  E  R  N  W  G  L  K  V  S  A  P

     401 CCCCTCAAAGTTGAGACTCTCAAGAACAGGTTTTTCAAGTGATTCCTCATTCAGTTTGGTTGGTAGAGAGAGAAACTGGGGTTTGAAGGTGAGTGCTCCA

 L  R  F  Q  E  V  E  E  E  S  E  E  R  G  S  V  I  V  N  G  V  D  E  F  D  P  G  A  P  P  P  F  K   ·

     501 CTGAGATTTCAGGAGGTGGAGGAAGAGAGTGAAGAGAGAGGGAGTGTAATAGTAAATGGTGTTGATGAATTCGACCCTGGTGCACCACCACCATTCAAGC

L  S  D  I  R  A  A  I  P  K  H  C  W  V  K  D  P  W  R  S  M  S  Y  V  V  R  D  V  V  V  V  F  G  L ·

     601 TGTCTGATATTCGGGCAGCCATTCCTAAACATTGTTGGGTTAAGGATCCATGGAGGTCTATGAGCTATGTTGTAAGAGATGTTGTTGTTGTTTTTGGATT

  A  A  A  A  A  Y  F  N  N  W  A  V  W  P  I  Y  W  F  A  Q  S  T  M  F  W  A  L  F  V  L  G  H  D

     701 GGCTGCCGCTGCAGCCTATTTCAACAACTGGGCTGTTTGGCCTATCTACTGGTTCGCTCAGTCAACTATGTTTTGGGCTCTGTTTGTTCTTGGACATGAT

 C  G  H  G  S  F  S  N  D  P  K  L  N  S  V  A  G  H  L  L  H  S  S  I  L  V  P  Y  H  G  W  R  I   ·

     801 TGTGGACATGGGAGCTTTTCTAATGATCCCAAGTTGAATAGTGTTGCTGGTCACCTGCTTCACTCTTCCATTCTTGTTCCTTATCATGGATGGAGAATTA

S  H  R  T  H  H  Q  N  H  G  H  V  E  N  D  E  S  W  H  P  I  P  E  K  I  Y  R  T  L  D  F  A  T  K ·

     901 GTCACAGGACACACCATCAGAACCATGGACATGTTGAAAATGATGAATCATGGCATCCAATACCTGAGAAGATATACAGGACTTTGGATTTTGCCACCAA

  K  L  R  F  T  L  P  F  P  M  L  A  Y  P  F  Y  L  W  G  R  S  P  G  K  K  G  S  H  F  H  P  D  S

    1001 GAAGTTGAGGTTCACTTTGCCTTTCCCCATGCTGGCTTATCCCTTCTATCTGTGGGGAAGAAGTCCTGGCAAGAAAGGCTCTCATTTCCATCCAGACAGT

 D  L  F  V  P  N  E  R  K  D  V  I  T  S  T  V  C  W  T  A  M  V  A  I  L  A  G  L  S  F  V  M  G   ·

    1101 GATTTGTTCGTTCCAAACGAGAGGAAAGATGTTATCACCTCAACTGTTTGTTGGACAGCAATGGTTGCAATACTTGCAGGACTATCTTTTGTTATGGGTC

P  V  Q  L  L  K  L  Y  G  I  P  Y  I  G  F  V  A  W  L  D  L  V  T  Y  L  H  H  H  G  H  D  E  K  L ·

    1201 CTGTTCAGTTGCTTAAACTCTATGGCATACCTTATATTGGATTTGTGGCATGGCTTGATTTAGTTACCTACTTACACCACCATGGCCACGATGAGAAGCT

  P  W  Y  R  G  K  E  W  S  Y  L  R  G  G  L  T  T  L  D  R  D  Y  G  W  I  N  N  I  H  H  D  I  G

    1301 TCCTTGGTACCGAGGAAAGGAATGGAGTTACCTGAGAGGGGGGCTCACGACACTTGATCGCGATTATGGATGGATAAACAACATCCACCATGACATAGGG

 T  H  V  I  H  H  L  F  P  Q  I  P  H  Y  H  L  I  E  A  T  A  A  A  K  P  V  L  G  K  Y  Y  K  E   ·

    1401 ACGCATGTTATACATCACCTCTTCCCACAAATACCACACTACCATTTGATAGAAGCAACTGCAGCAGCTAAGCCAGTTCTAGGAAAATATTACAAGGAGC

P  K  K  S  G  P  F  P  F  Y  L  L  G  V  L  Q  K  S  M  K  K  D  H  Y  V  S  D  T  G  D  I  V  Y  Y ·

    1501 CTAAGAAATCAGGCCCCTTTCCATTCTACTTGTTGGGAGTCCTCCAAAAAAGCATGAAAAAGGATCACTATGTGAGTGACACGGGCGATATCGTTTACTA

  Q  T  D  P  E  L  N  *

    1601 CCAGACCGATCCTGAGCTGAAT**TGA**ACTCAAGAATGATAAGAGTTTGAATGTTGTTATCAGTATATGTAAAAGCTGTCTCTAATTGAGTTCTCGAGACCT

    1701 CTAAGGATCGCGTTCAGCTGCAGAATTGATATATATATTTTTTTCTCTTCAACGATGGAGAGGCGATACTAGAGATTACAGAATATTGATGTGTATTTGT

    1801 ATGAGGATGTACTGATGCTATAATGTATCTTGATAATTAATGAGAGGGAAAGCCAGATTATTCTC*TAAAAAAAAAAAAAAAAAAAAAAAAAAAAA*

E: *PfFAD7a*mRNA

       1 *G*GAGGCATAGGTGGTGGAGTCGGAAAGAGAGAAGAGATAGAGGCTGAAAATTGTGTATTTAAATGAATCACATTCTGCTCGGGTTATAAGCCTCAAGAAA

     101 ATCCAAATCAACAAGTTGGGATTCTTGGAAGCCATAAATTTGAGGAGAGAATCCCTTGTATTATCTACTAACTTAAGCATATACATACATATTCTGCAAA

     201 CCCCTCAAGTCCACACACTATTCTCTCTCTTTCTCTCTCATTCTAGTGAGAGAACCCCAATAAAGGTGAAATCTTGATTACTTTCAGAGTTGGGGCTCTC

         M  A  S  W  V  L  S  E  C  G  L  R  P  L  P  R  I  Y  P  K  P  R  T  G  Q  Y  L  S  N  S  N ·

     301 TCTCTGAA**ATG**GCGAGTTGGGTGTTATCAGAATGTGGTTTAAGGCCACTTCCAAGAATATACCCCAAGCCAAGAACTGGCCAGTATCTCTCCAATTCCAA

· P  S  K  L  R  L  S  R  T  G  F  S  S  D  S  S  F  S  L  V  G  R  E  R  N  W  G  L  K  V  S  A  P

     401 CCCCTCAAAGTTGAGACTCTCAAGAACAGGTTTTTCAAGTGATTCCTCATTCAGTTTGGTTGGTAGAGAGAGAAACTGGGGTTTGAAGGTGAGTGCTCCA

 L  R  F  Q  E  V  E  E  E  S  E  E  R  G  S  V  I  V  N  G  V  D  E  F  D  P  G  A  P  P  P  F  K   ·

     501 CTGAGATTTCAGGAGGTGGAGGAAGAGAGTGAAGAGAGAGGGAGTGTAATAGTAAATGGTGTTGATGAATTCGACCCTGGTGCTCCACCACCATTCAAGC

L  S  D  I  R  A  A  I  P  K  H  C  W  V  K  D  P  W  R  S  M  S  Y  V  V  R  D  V  V  V  V  F  G  L ·

     601 TGTCTGATATTCGGGCAGCCATTCCTAAACATTGTTGGGTTAAGGATCCATGGAGGTCTATGAGCTATGTTGTAAGAGATGTTGTTGTTGTTTTTGGATT

  A  A  A  A  A  Y  F  N  N  W  A  V  W  P  I  Y  W  F  A  Q  S  T  M  F  W  A  L  F  V  L  G  H  D

     701 GGCTGCCGCTGCAGCCTATTTCAACAACTGGGCTGTTTGGCCTATCTACTGGTTCGCTCAGTCAACTATGTTTTGGGCTCTGTTTGTTCTTGGACATGAT

 C  G  H  G  S  F  S  N  D  P  K  L  N  S  V  A  G  H  L  L  H  S  S  I  L  V  P  Y  H  G  W  R  I   ·

     801 TGTGGACATGGGAGCTTTTCTAATGATCCCAAGTTGAATAGTGTTGCTGGTCACCTGCTTCACTCTTCCATTCTTGTTCCTTATCATGGATGGAGAATTA

S  H  R  T  H  H  Q  N  H  G  H  V  E  N  D  E  S  W  H  P  I  P  E  K  I  Y  R  T  L  D  F  A  T  K ·

     901 GTCACAGGACACACCATCAGAACCATGGACATGTTGAAAATGATGAATCATGGCATCCAATACCTGAGAAGATATACAGGACTTTGGATTTTGCCACCAA

  K  L  R  F  T  L  P  F  P  M  L  A  Y  P  F  Y  L  W  G  R  S  P  G  K  K  G  S  H  F  H  P  D  S

    1001 GAAGTTGAGGTTCACTTTGCCTTTCCCCATGCTGGCTTATCCCTTCTATCTGTGGGGAAGAAGTCCTGGCAAGAAAGGCTCTCATTTCCATCCAGACAGT

 D  L  F  V  P  N  E  R  K  D  V  I  T  S  T  V  C  W  T  A  M  V  A  I  L  A  G  L  S  F  V  M  G   ·

    1101 GATTTGTTCGTTCCAAACGAGAGGAAAGATGTTATCACCTCAACTGTTTGTTGGACAGCAATGGTTGCAATACTTGCAGGACTATCTTTTGTTATGGGTC

P  V  Q  L  L  K  L  Y  G  I  P  Y  I  G  F  V  A  W  L  D  L  V  T  Y  L  H  H  H  G  H  D  E  K  L ·

    1201 CTGTTCAGTTGCTTAAACTCTATGGCATACCTTATATTGGATTTGTGGCATGGCTTGATTTAGTTACCTACTTACACCACCATGGCCACGATGAGAAGCT

  P  W  Y  R  G  K  E  W  S  Y  L  R  G  G  L  T  T  L  D  R  D  Y  G  W  I  N  N  I  H  H  D  I  G

    1301 TCCTTGGTACCGAGGAAAGGAATGGAGTTACCTGAGAGGGGGGCTCACGACACTTGATCGCGATTATGGATGGATAAACAACATCCACCATGACATAGGG

 T  H  V  I  H  H  L  F  P  Q  I  P  H  Y  H  L  I  E  A  T  A  A  A  K  P  V  L  G  K  Y  Y  K  E   ·

    1401 ACGCATGTTATACATCACCTCTTCCCACAAATACCACACTACCATTTGATAGAAGCAACTGCAGCAGCTAAGCCAGTTCTAGGAAAATATTACAAGGAGC

P  K  K  S  G  P  F  P  F  Y  L  L  G  V  L  Q  K  S  M  K  K  D  H  Y  V  S  D  T  G  D  I  V  Y  Y ·

    1501 CTAAGAAATCAGGCCCCTTTCCATTCTACTTGTTGGGAGTCCTCCAAAAAAGCATGAAAAAGGATCACTATGTGAGTGACACGGGCGATATCGTTTACTA

  Q  T  D  P  E  L  N  *

    1601 CCAGACCGATCCTGAGCTGAAT**TGA**ACTCAAGAATGATAAGAGTTTGAATGTTGTTATCAGTATATGTAAAAGCTGTCTCTAATTGAGTCCTCGAGACCT

    1701 CTAAGGATCGCGTTCAGCTGCAGAATTGATATATATATTTTTTTCTCTTCAACGATGGAGAGGCGATACTAGAGATTACAGAATATTGATGTGTATTTGT

    1801 ATGAGGATGTACTGATGCTATAATGTATCTTGATAATTAATGAGAGGGAAAGCCAGATTATTCTC*TAAAAAAAAAAAAAAAAAAAAAAAAAAAAA*

F: *PfFAD7b*mRNA

       1 *G*GGGAAAGA*G*AGAAGAAAGAGAGGCAGATGCGAATTGCGATGGCTGTGGCAGTGTCTATATATATATCACCCATCTCCTCTCTCTCTCTCTCTCTCTCCT

     101 CCCTAAAAACAACCCACAACGCCAACTTGTGTCCACTTGCGGCCCTAACGTGAGAGGAGAGGAGAGGAGAGGAGCCCTTTTATCATCAACTGCTCACCTC

     201 TCAATCCAGCGGAATCATCTGCATTCCTGCACATAAAATACGTGTATACATACAAGCAATATTCTGCCAAGCCCTTAAGTCCACAAGTTTCTCTAGAGAG

                                                                                 M  A  S  F  V  I  S ·

     301 AGAAAGAGAGAGCTTTTTTAAAAGGTGAATTCTTGGTTGGGTTCTGAATTGGGCCGCCTCTCTTTCTTTCCCTCTCTGTA**ATG**GCGAGTTTCGTTATATC

  E  C  G  L  K  P  L  P  R  I  Y  P  K  P  R  A  A  Q  P  L  S  S  S  N  L  R  F  S  R  T  N  Q  R

     401 AGAATGTGGCTTGAAGCCACTTCCAAGAATCTATCCCAAACCAAGAGCTGCCCAGCCTCTCTCGAGTTCTAATCTGAGATTTTCAAGAACAAATCAACGG

 F  N  S  S  F  C  S  S  T  G  I  I  K  E  R  N  W  A  L  R  V  S  A  P  L  R  I  Q  P  V  E  E  E   ·

     501 TTTAATTCTTCATTCTGTTCATCAACTGGGATTATTAAGGAACGGAATTGGGCTTTGAGAGTGAGTGCCCCATTAAGAATTCAGCCAGTGGAAGAAGAGA

N  R  A  I  N  G  G  E  E  F  D  P  A  A  P  P  P  F  K  L  S  D  I  K  A  A  I  P  K  H  C  W  V  K ·

     601 ACAGAGCGATAAACGGCGGCGAAGAATTCGACCCGGCGGCGCCGCCTCCGTTTAAGTTGTCCGATATAAAGGCAGCCATTCCGAAGCATTGTTGGGTGAA

  D  P  W  R  S  V  S  Y  V  V  R  D  V  V  A  V  F  G  M  A  A  A  A  A  Y  F  N  N  W  L  V  W  P

     701 GGACCCATGGAGGTCTGTGAGCTATGTGGTGAGGGATGTGGTGGCGGTTTTTGGGATGGCGGCGGCGGCGGCCTATTTCAACAATTGGCTTGTTTGGCCT

 L  Y  W  F  A  Q  S  T  L  F  W  A  L  F  V  L  G  H  D  C

     801 TTGTATTGGTTTGCTCAGAGCACCTTATTCTGGGCTCTCTTTGTTCTTGGCCATGACTGGTAATTTATTTATTTTTTGAAGAAAGTCTTTATTTTTGTTG

                                                   G  H  G  S  F  S  N  N  P  K  L  N  S  V  F  G  H ·

     901 GTGTGTGTGTGAGATAAAGATTCAATTTTGGTGGTTTTTGTTGGTGCAGTGGTCATGGAAGCTTTTCAAACAACCCCAAGCTGAATAGTGTGTTTGGCCA

  L  L  H  S  S  I  L  V  P  Y  H  G  W

    1001 TCTTCTTCACTCTTCAATTCTGGTGCCCTACCATGGATGGTATTTTTTCATGTCTAGTATTTGATATTTCACTGTTTGTGTTTGTGTTTATTGTTAGTTA

                                                                             R  I  S  H  R  T  H  H

    1101 GGTAGACTTTGTTAAGTAACTAAAAGGAGCCTAGGACAGCTCTGTTAATATCATGTTGATTGTTGAATGATGCAGGAGAATTAGCCATAGAACTCATCAT

 Q  N  H  G  H  V  E  N  D  E  S  W  H  P

    1201 CAGAACCATGGACATGTTGAGAATGATGAATCTTGGCACCCGGTAAGTTTTTGTCGAATCGAAAATCTTAAAATCTAGATTGTTGATGCATTGTAGAAGC

                                  L  P  E  K  I  Y  N  S  L  D  N  N  T  K  M  L  R  F  T  L  P  F   ·

    1301 CAGGAGTCAATGCTGTTCCTATCTTAATTGCAGTTACCTGAGAAGATTTACAATAGCTTGGATAATAATACCAAGATGTTGAGGTTCACATTGCCTTTCC

P  M  L  A  Y  P  F  Y  L

    1401 CTATGTTGGCATACCCCTTTTATCTGGTAAGTTGCTTACTTCTTTGATATGGAACATCTTTTCTGTGCGATTTCTTCAATGAACATGTTTGCCTGAATTT

                               W  S  R  S  P  G  K  K  G  S  H  F  H  P  E  S  D  L  F  V  P  N  E   ·

    1501 GTTTTTCGTGTTCTTTGTGTTTAATTGTAGTGGAGTAGAAGTCCCGGGAAGAAAGGCTCTCATTTCCACCCAGAGAGTGATTTGTTTGTGCCAAATGAGA

R  K  D  V  I  T  S  T  V  C  W  T  A  M  A  A  L  L  V  G  L  S  F  V  I  G  P  L  Q  L  L  K  L  Y ·

    1601 GGAAAGACGTTATTACCTCAACAGTTTGTTGGACTGCAATGGCTGCATTGCTCGTAGGACTATCTTTTGTTATCGGTCCACTCCAGCTGCTCAAACTATA

  G  V  P  Y  L

    1701 CGGCGTTCCTTACTTGGTTAGTGATTTGAATCCATTGATTCTGTAAATACTTTTTCCGTCTTCATGAAGCAAAGACTGGTTATTTGATATATCAAGTGCG

    1801 GATTTTTTTGTTTTTCGTCTTGTCAAAGATTTGAACGATGCTTTAGGATTATCTATCTGTCTTGGACTTCTTGGTAAAACCTGGAAATTTGACTTTGTCT

     G  F  V  A  W  L  D  L  V  T  Y  L  H  H  H  G  H  E  D  K  L  P  W  Y  R  G  K

    1901 GCAGGGATTCGTAGCGTGGCTTGATCTTGTGACCTATTTGCATCACCACGGGCATGAAGATAAGCTCCCTTGGTACCGTGGAAAGGTATACTAAACAACT

    2001 TAGAATATTTTGAACCTTTTAAAATCCCAGGAAGACGAAAACAGAGGTCTTTTGTTGAAAATAATCAACTTGGAAATATAATTATGCTCGACTTATATCA

                                                                         E  W  S  Y  L  R  G  G  L   ·

    2101 AAGTTGTCATGATTCACTTAACTTGTTTAGTCTCTTGAAAGTTGGTTTCTTTTTTCTTTTTTGGTAAAATAGGAATGGAGTTATCTGAGAGGGGGGCTCA

T  T  L  D  R  D  Y  G  L  I  N  N  I  H  H  D  I  G  T  H  V  I  H  H  L  F  P  Q  I  P  H  Y  H  L ·

    2201 CGACACTTGATCGTGACTACGGATTGATCAACAACATCCACCATGACATAGGAACTCATGTCATACACCACCTCTTCCCCCAAATCCCACACTACCATTT

  I  E  A

    2301 GATAGAAGCTGTAAGTTATCCATTTTGCTCGAAAAGCTTTACTTCGACGATCAAATTTATGTTGCAGCTTTTTGACTCTTAACTTTGGCCAATTCTTCGA

       T  E  A  A  K  G  V  L  G  K  Y  Y  R  E  P  K  K  S  G  P  L  P  L  H  L  L  G  D  L  L  R   ·

    2401 ATGCAGACTGAAGCAGCTAAGGGGGTATTAGGCAAGTACTACAGGGAGCCGAAAAAGTCGGGCCCTCTACCGTTACACTTGTTGGGAGACCTCCTGAGAA

S  M  K  K  D  H  Y  V  S  D  T  G  D  I  V  Y  Y  Q  T  D  P  Q  L  N  G  G  R  K  S  *

    2501 GCATGAAGAAGGATCACTACGTGAGCGACACCGGCGACATTGTCTATTATCAGACAGATCCTCAGCTCAATGGAGGTCGCAAATCT**TAG**GCTGTAGTGAA

    2601 AGAATTTATCGATTCTTTGTCAGCTGAGTCTAATTATTAGTGGCTGTTAAGGATAGTGTATGAGCTCATATCATACTACCATGCCAACATAGGTAATTTT

    2701 TTTCGAATACAATTGATTTTTGCTTCAATCCATTGATGGAGCACCGTATAAATACGTATTTATTGCATTTGTCCTAGAATATGAATGATAAAGTAATGAA

    2801 TTTCCATGTCGAAGGGAAACTCTTTTTCCCCACGGCTTCTTGTTCTTC*C*

G: *PfFAD8a*gene

       1 *G*GGGAAAGA*G*AGAAGAAAGAGAGGCAGATGCGAATTGCGATGGCTGTGGCAGTGTCTATATATATATCACCCATCTCCTCTCTCTCTCTCTCTCTCTCCT

     101 CCCTAAAAACAACCCACAACGCCAACTTGTGTCCACTTGCGGCCCTAACGTGAGAGGAGAGGAGAGGAGAGGAGCCCTTTTATCATCAACTGCTCACCTC

     201 TCAATCCAGCGGAATCATCTGCATTCCTGCACATAAAATACGTGTATACATACAAGCAATATTCTGCCAAGCCCTTAAGTCCACAAGTTTCTCTAGAGAG

                                                                                 M  A  S  F  V  I  S ·

     301 AGAAAGAGAGAGCTTTTTTAAAAGGTGAATTCTTGGTTGGGTTCTGAATTGGGCCGCCTCTCTTTCTTTCCCTCTCTGTA**ATG**GCGAGTTTCGTTATATC

  E  C  G  L  K  P  L  P  R  I  Y  P  K  P  R  A  A  Q  P  L  S  S  S  N  L  R  F  S  R  T  N  Q  R

     401 AGAATGTGGCTTGAAGCCACTTCCAAGAATCTATCCCAAACCAAGAGCTGCCCAGCCTCTCTCGAGTTCTAATCTGAGATTTTCAAGAACAAATCAACGG

 F  N  S  S  F  C  S  S  T  G  I  I  K  E  R  N  W  A  L  R  V  S  A  P  L  R  I  Q  P  V  E  E  E   ·

     501 TTTAATTCTTCATTCTGTTCATCAACTGGGATTATTAAGGAACGGAATTGGGCTTTGAGAGTGAGTGCCCCATTAAGAATTCAGCCAGTGGAAGAAGAGA

N  R  A  I  N  G  G  E  E  F  D  P  A  A  P  P  P  F  K  L  S  D  I  K  A  A  I  P  K  H  C  W  V  K ·

     601 ACAGAGCGATAAACGGCGGCGAAGAATTCGACCCGGCGGCGCCGCCTCCGTTTAAGTTGTCCGATATAAAGGCAGCCATTCCGAAGCATTGTTGGGTGAA

  D  P  W  R  S  V  S  Y  V  V  R  D  V  V  A  V  F  G  M  A  A  A  A  A  Y  F  N  N  W  L  V  W  P

     701 GGACCCATGGAGGTCTGTGAGCTATGTGGTGAGGGATGTGGTGGCGGTTTTTGGGATGGCGGCGGCGGCGGCCTATTTCAACAATTGGCTTGTTTGGCCT

 L  Y  W  F  A  Q  S  T  L  F  W  A  L  F  V  L  G  H  D  C  G  H  G  S  F  S  N  N  P  K  L  N  S   ·

     801 TTGTATTGGTTTGCTCAGAGCACCTTATTCTGGGCTCTCTTTGTTCTTGGCCATGACTGTGGTCATGGAAGCTTTTCAAACAACCCCAAGCTGAATAGTG

V  F  G  H  L  L  H  S  S  I  L  V  P  Y  H  G  W  R  I  S  H  R  T  H  H  Q  N  H  G  H  V  E  N  D ·

     901 TGTTTGGCCATCTTCTTCACTCTTCAATTCTGGTGCCCTACCATGGATGGAGAATTAGCCATAGAACTCATCATCAGAACCATGGACATGTTGAGAATGA

  E  S  W  H  P  L  P  E  K  I  Y  N  S  L  D  N  N  T  K  M  L  R  F  T  L  P  F  P  M  L  A  Y  P

    1001 TGAATCTTGGCACCCGTTACCTGAGAAGATTTACAATAGCTTGGATAATAATACCAAGATGTTGAGGTTCACATTGCCTTTCCCTATGTTGGCATACCCC

 F  Y  L  W  S  R  S  P  G  K  K  G  S  H  F  H  P  E  S  D  L  F  V  P  N  E  R  K  D  V  I  T  S   ·

    1101 TTTTATCTGTGGAGTAGAAGTCCCGGGAAGAAAGGCTCTCATTTCCACCCAGAGAGTGATTTGTTTGTGCCAAATGAGAGGAAAGACGTTATTACCTCAA

T  V  C  W  T  A  M  A  A  L  L  V  G  L  S  F  V  I  G  P  L  Q  L  L  K  L  Y  G  V  P  Y  L  G  F ·

    1201 CAGTTTGTTGGACTGCAATGGCTGCATTGCTCGTAGGACTATCTTTTGTTATCGGTCCACTCCAGCTGCTCAAACTATACGGCGTTCCTTACTTGGGATT

  V  A  W  L  D  L  V  T  Y  L  H  H  H  G  H  E  D  K  L  P  W  Y  R  G  K  E  W  S  Y  L  R  G  G

    1301 CGTAGCGTGGCTTGATCTTGTGACCTATTTGCATCACCACGGGCATGAAGATAAGCTCCCTTGGTACCGTGGAAAGGAATGGAGTTATCTGAGAGGGGGG

 L  T  T  L  D  R  D  Y  G  L  I  N  N  I  H  H  D  I  G  T  H  V  I  H  H  L  F  P  Q  I  P  H  Y   ·

    1401 CTCACGACACTTGATCGTGACTACGGATTGATCAACAACATCCACCATGACATAGGAACTCATGTCATACACCACCTCTTCCCCCAAATCCCACACTACC

H  L  I  E  A  T  E  A  A  K  G  V  L  G  K  Y  Y  R  E  P  K  K  S  G  P  L  P  L  H  L  L  G  D  L ·

    1501 ATTTGATAGAAGCTACTGAAGCAGCTAAGGGGGTATTAGGCAAGTACTACAGGGAGCCGAAAAAGTCGGGCCCTCTACCGTTACACTTGTTGGGAGACCT

  L  R  S  M  K  K  D  H  Y  V  S  D  T  G  D  I  V  Y  Y  Q  T  D  P  Q  L  N  G  G  R  K  S  *

    1601 CCTGAGAAGCATGAAGAAGGATCACTACGTGAGCGACACCGGCGACATTGTCTATTATCAGACAGATCCTCAGCTCAATGGAGGTCGCAAATCT**TAG**GCT

    1701 GTAGTGAAAGAATTTATCGATTCTTTGTCAGCTGAGTCTAATTATTAGTGGCTGTTAAGGATAGTGTATGAGCTCATATCATACTACCATGCCAACATAG

    1801 GTAATTTTTTTCGAATACAATTGATTTTTGCTTCAATCCATTGATGGAGCACCGTATAAATACGTATTTATTGCATTTGTCCTAGAATATGAATGATAAA

    1901 GTAATGAATTTCCATGTCGAAGGGAAACTCTTTTTCCCCACGGCTTCTTGTTCTTC*CAAAAAAAAAAAAAAAAAAAAAAAAAAA*

H: *PfFAD8a*mRNA

       1 *G*GGGAAAGA*G*AGAAGAAAGAGAGGCAGATGCGAATTGCGATGGCT*G*TAGCACTGTGTGTATATATATCAGCCATCTCCTCTCTCTCTCTCTCCTCCCTAG

     101 AAAAAACCCACAACACCAACTTGTGTCCACTTGCGGCCCTAACGTGAGAGGAGAGGAGAGGAGCCCTTTTATCATCAACTACTCACCTCTCAATCCAGCG

     201 GAATCATCTGCATTCCTGCACATAAATACGTGTGTACATACAAACAATATTCTGCCAAGCCCTTAAGTCCACAAGTTTCTCTAGAGAGAGAAAGAGGGAG

                                                                           M  A  S  F  V  I  S  E  C ·

     301 CTTTTTTAAAAGGTGAATTCTTGGTTGGGTTCTGAATTGGGCCGCCTCTCTCTCTCTTTCTTTCCCTCTCTGTAATGGCGAGTTTCGTTATATCAGAATG

· G  L  K  P  L  P  R  I  Y  P  K  P  R  A  A  Q  P  L  S  S  S  N  L  R  F  S  R  T  N  Q  R  F  N

     401 TGGCTTGAAGCCACTTCCAAGAATCTATCCCAAACCGAGAGCTGCCCAGCCTCTCTCGAGTTCTAATCTGAGATTTTCAAGAACAAATCAACGGTTTAAT

 S  S  F  C  S  S  S  G  I  N  K  E  R  N  W  A  L  R  V  S  A  P  L  R  I  Q  P  V  E  E  E  N  R   ·

     501 TCTTCATTCTGTTCATCAAGTGGGATTAATAAGGAACGGAATTGGGCTTTGAGAGTGAGTGCCCCATTAAGAATTCAGCCAGTGGAAGAAGAGAACAGAG

A  I  N  G  G  E  E  F  D  P  A  A  P  P  P  F  K  L  S  D  I  K  A  A  I  P  K  H  C  W  V  K  D  P ·

     601 CGATAAACGGCGGCGAAGAATTCGACCCGGCGGCGCCGCCTCCGTTTAAGTTGTCCGATATTAAGGCAGCCATTCCGAAGCATTGTTGGGTGAAGGACCC

  W  R  S  V  S  Y  V  V  R  D  V  V  A  V  F  G  M  A  A  A  A  A  Y  F  N  N  W  L  V  W  P  L  Y

     701 ATGGAGGTCTGTGAGCTATGTGGTGAGGGATGTGGTGGCGGTTTTTGGGATGGCGGCGGCGGCGGCCTATTTCAATAATTGGCTTGTTTGGCCTTTGTAT

 W  F  A  Q  S  T  L  F  W  A  L  F  V  L  G  H  D

     801 TGGTTTGCTCAGAGCACCTTATTCTGGGCTCTCTTTGTTCTTGGGCATGATTG

I: *PfFAD8b*mRNA

                                   M  A  V  S  S  G  A  R  L  S  E  S  G  A  E  G  G  E  P  Y  A  G

       1 ***C***TTCAA*A*CTGAAAACCAGCTCCTCGTCCCCAAAA**ATG**GCCGTTTCTTCCGGTGCCCGTCTCTCCGAGAGTGGCGCTGAAGGAGGAGAGCCCTACGCCGGC

 Q  C  E  H  L  E  G  I  G  K  R  A  A  D  K  F  D  P  A  A  P  P  P  F  K  I  A  D  I  R  A  A  I

     101 CAATGCGAGCACCTCGAAGGAATCGGAAAGCGCGCCGCCGACAAATTCGACCCCGCCGCGCCGCCGCCGTTCAAGATCGCCGACATCCGCGCCGCGATCC

P  P  H  C  W  V  K  D  P  L  R  S  L  S  Y  V  A  W  D  L  I  A  V  A  A  L  L  A  A  A  A  Y  F  D

     201 CGCCGCATTGCTGGGTGAAGGACCCCCTCCGCTCCCTCAGCTACGTCGCCTGGGATCTCATCGCCGTCGCCGCGCTCCTCGCCGCCGCCGCCTACTTCGA

· S  W  I  F  W  P  I  Y  W  A  A  Q  G  T  M  F  W  A  L  F  V  L  G  H  D  C

     301 CAGCTGGATCTTCTGGCCGATCTACTGGGCGGCCCAGGGCACCATGTTTTGGGCCTTGTTCGTCCTCGGCCACGATTGGTAATTTCATTTCCTGTTTTTT

     401 TTTTTTTTTTTTCTCGATTAAATCGAATCACATTTTACTTTATTCAGTTATAAATTAAATCTCACTAATACAGTCATTTCTCATTTGCTATTTAATTGAT

     501 TTATTTATATCTCATTTTATAAAGATCTTTTCTTTATTTATTAATATACGTATCAATGTCAATTTCTACAATTAATGAGAAGTAGAAAGAGTACTATAAT

     601 TGGTGTTGAAGCTACATTGACAAATTGAACAATAATTTTGTAGGTAGCTCCTATAATTGTAGTATATGTATGGATGCATTTTATTTTGTTTAGTTTATTG

                                                      G  H  G  S  F  S  D  S  T  T  L  N  N  V  V  G

     701 TGGTATAAACTTTGTATTGGTATTCAATTTAGTGCATGCTGATTCATTGCAGTGGGCACGGGAGTTTTTCAGACAGCACCACGCTAAATAACGTGGTGGG

· H  I  L  H  S  S  I  L  V  P  Y  H  G  W

     801 ACACATACTACATTCTTCAATTCTTGTACCTTATCATGGATGGTTAGTATTTCGACTTAAAAACTTTTTATTTATGTTTTTGCATCATCTACCTCCGTTC

     901 ACAAATAATAGTCTTGTATGTTTTTTTAGCCCGGTCATAAAAATCATAGCTTGTTTCCATTTATTCTGTATCGATCTTTAAGATGAGACATTTTTCTTCG

    1001 CAATGCTACAATTACTTTTGTTAAAAATTGATCTCGATTCTCGATCGGTTTATAAACATATTCTTATTCGGTCATTCTTAGTAAAACTTGTGCTGATAAA

                                                                         R  I  S  H  R  T  H  H  Q

    1101 GGATGACAATATTATATTTTGTCACAAATGTCACACAAATATGGCTAATATAGATCTGTATTTTTACTTAGGCGAATTAGCCATCGAACACACCACCAGA

N  H  G  H  V  E  K  D  E  S  W  V  P

    1201 ATCATGGTCACGTGGAGAAGGACGAGTCATGGGTGCCGGTAAACATTTTTTTCTACAAAAACATCTTGAATCTTTTATCATAGAGATTGATTAATAAGTT

                L  P  E  N  L  Y  K  Q  L  D  F  S  T  K  F  L  R  Y  K  I  P  F  P  M  F  A  Y  P

    1301 AATTTATTAATGCAGCTGCCTGAGAATTTATACAAGCAGCTAGATTTCTCAACTAAATTCTTGAGATACAAAATCCCATTCCCCATGTTTGCCTACCCTC

L  Y  L                                                                                    W  Y  R

    1401 TCTACTTGGTATGGTTCTCTCTCTCATCAAATTTAAGTAATTGAAATTTCCAACATGGATATTAAATTAAGTTTATTTATTTAATTTCAGTGGTATAGAA

S  P  G  K  T  G  S  H  F  N  P  D  S  S  L  F  K  P  N  E  R  D  L  V  I  T  S  T  V  C  W  A  A  M

    1501 GTCCTGGAAAAACTGGATCTCATTTCAACCCAGATAGCAGTTTGTTCAAACCCAATGAGAGAGATTTGGTCATCACTTCCACCGTTTGTTGGGCTGCAAT

· V  A  F  L  L  Y  A  S  T  I  V  G  P  T  M  L  F  K  L  Y  G  V  P  Y  L

    1601 GGTTGCTTTTCTCCTTTATGCTTCCACCATTGTTGGCCCAACCATGTTGTTCAAGCTCTACGGCGTGCCTTATTTGGTAAGACACTGTCACACTACTATG

    1701 AAATCGAGAGTATTGCTATTTCGAATCGAGTCCAAATGAGACACAATACGAAATAGAGAATATTGTTATACTCCTATCAAATAAATCATAGGGATAATAG

                                                                                      I  F  V  V  W

    1801 CTATTCATGGAATTATCAAATTTTGTAATTTAAATAACTATCATCTTAGAATAAAATTCTGAAGTCATGGGTTCGAATCTTGTAGATATTCGTTGTGTGG

 L  D  T  V  T  Y  L  H  H  H  G  Y  D  K  K  L  P  W  Y  R  S  K

    1901 TTGGACACGGTGACATATTTGCATCACCATGGTTACGACAAGAAGCTGCCATGGTACCGCAGCAAGGTATTATTTTAATTCAGTATTGTTTCTATTTTTG

    2001 TTTAAGTTCTGTCACTAGCATATGTTTCCCAATTTTAATGCCACATTAACTAACACAAATATCTACATTGAAATCGACTATATTAGTATTTGTTTAGCTG

    2101 AAAATCTCACCTACCCATCCAGTGTATTTTAATATATGTTCACAAAAGATGTATTTTACTTTTGTCCTAGATCTATCTCTTTTATTATTATGATTCATAA

    2201 ATTTTAAACATTAATTCAAAAATCGTCACATATACTTAAATTAGGTATTTTTTCTAATTCTTGTTTTAATTTGGTAACATCTCCTTGTTATAATTGGGAA

    2301 AATATTCTGAACTATTAGTTCAAAATATTGCGATCGTTCAATTTCCACTATTGCTATGACTAGAGATTATGTTCCTTACTTTTTGTTGTTTGGTTGCCAT

    2401 TTTTTCTCGAAAACTGAATCAATGACACTGTATCTTGACTTAGTGTTAAGATCATCTTAGCCAATTTATTTCGGCTTATCTCATACCGTATCTCATTATT

    2501 ATAATGTCTAGTGAGTCGAATGACCGTGGTCCAGTCAAGAGTGACGATGTCTACTCAAAATATCTTGAATTAGAATCTCACCAACCTTTTCTAAATCAGA

                                                                                      E  W  S  Y  L

    2601 GATTTCCAGTCGTGTGCTCCGACACAAGTCATGTGCTATGAAAGTAGTAGTTATTAATGGTTCCTTTTTTGTCTTGTAATAACAGGAATGGAGTTATTTA

 R  G  G  L  T  T  V  D  Q  D  Y  G  I  F  N  K  I  H  H  D  I  G  T  H  V  I  H  H  L  F  P  Q  I

    2701 AGAGGAGGATTGACAACAGTAGATCAAGACTATGGAATATTCAACAAAATTCACCATGATATTGGCACCCATGTTATACACCACCTCTTCCCTCAGATCC

P  H  Y  H  L  V  E  A

    2801 CACACTACCATTTAGTGGAAGCGGTGAGATCTCTATTCATAATTAAATTTATTTAATTAGTCCCTAATCACGTGATCATCTTAGCATAACAACAACATTA

    2901 AATCTTATATATTCTTGCACTAGAAATATTATGCTATATATCTATGTTTAGATTTTTTAACTTAGTAGTACTATCATTTGAAACTGACTATTATTTTTAT

    3001 AAATGACATAAAAATCTTAGTATGATTTGTTCTTTATTCACATTTTGGAAATTAGTGAGAAAATGGACAAATCATTCTTTGATTTTTGTGCAACTTTGTA

    3101 ACATCAATGACTTAATGATATATAAAAACATAAGTTATGTAGCATTACATTCCAATACCTGAAAATTCAAATTTTTTTAAACATGTTTTTTACCGATGAA

                           T  R  E  A  K  R  V  L  G  N  Y  Y  R  E  P  R  K  S  G  P  V  P  F  H  L

    3201 ATGATATACGAGAGTGGTGTTCACAGACGAGGGAGGCGAAGAGGGTGCTCGGAAATTACTACAGAGAGCCCAGAAAATCTGGACCAGTTCCATTTCACTT

· I  P  T  L  L  K  S  L  S  R  D  H  Y  V  S  D  N  G  D  I  V  Y  Y  Q  T  D  S  Q  L  F  S  S  K

    3301 GATTCCCACATTGTTGAAAAGTCTAAGCAGAGATCATTATGTCAGTGATAATGGAGACATAGTTTACTATCAAACTGATAGTCAGCTATTTTCGTCAAAA

 E  I  *

    3401 GAGATT**TAG**TGATGGGTTCTAAAATAATCAAATTGTATTAGATTTATTTCATGGTAGCTTTTTGGGGCCACATTGTTTTAATTAGAGAAATGATTGTGTG

    3501 CCTCAAGAAGAATTTTAAATAATTGTATCGAGTTTTTCAGAATGCAATTTTGCTCCCCCTTGTTTT*T*TGTT*T*TATGATTATGTAATATCCCTTTA*AATAA*

    3601 *A*GTTTTTAGGTGATTGTGTGC*C*AGTTTTGTGGACTTTTGAAGATTACTTGTATTATT*T*AAAAAGAAAAGAAGAGGTTTGG***T***A*T*ATTTTGT*T*

J: *ShFAD3-1*gene

                                   M  A  V  S  S  G  A  R  L  S  E  S  G  A  E  G  G  E  P  Y  A  G

       1 ***C***TTCAA*A*CTGAAAACCAGCTCCTCGTCCCCAAAA**ATG**GCCGTTTCTTCCGGTGCCCGTCTCTCCGAGAGTGGCGCTGAAGGAGGAGAGCCCTACGCCGGC

 Q  C  E  H  L  E  G  I  G  K  R  A  A  D  K  F  D  P  A  A  P  P  P  F  K  I  A  D  I  R  A  A  I

     101 CAATGCGAGCACCTCGAAGGAATCGGAAAGCGCGCCGCCGACAAATTCGACCCCGCCGCGCCGCCGCCGTTCAAGATCGCCGACATCCGCGCCGCGATCC

P  P  H  C  W  V  K  D  P  L  R  S  L  S  Y  V  A  W  D  L  I  A  V  A  A  L  L  A  A  A  A  Y  F  D

     201 CGCCGCATTGCTGGGTGAAGGACCCCCTCCGCTCCCTCAGCTACGTCGCCTGGGATCTCATCGCCGTCGCCGCGCTCCTCGCCGCCGCCGCCTACTTCGA

  S  W  I  F  W  P  I  Y  W  A  A  Q  G  T  M  F  W  A  L  F  V  L  G  H  D  C  G  H  G  S  F  S  D

     301 CAGCTGGATCTTCTGGCCGATCTACTGGGCGGCCCAGGGCACCATGTTTTGGGCCTTGTTCGTCCTCGGCCACGATTGTGGGCACGGGAGTTTTTCAGAC

 S  T  T  L  N  N  V  V  G  H  I  L  H  S  S  I  L  V  P  Y  H  G  W  R  I  S  H  R  T  H  H  Q  N

     401 AGCACCACGCTAAATAACGTGGTGGGACACATACTACATTCTTCAATTCTTGTACCTTATCATGGATGGCGAATTAGCCATCGAACACACCACCAGAATC

H  G  H  V  E  K  D  E  S  W  V  P  L  P  E  N  L  Y  K  Q  L  D  F  S  T  K  F  L  R  Y  K  I  P  F

     501 ATGGTCACGTGGAGAAGGACGAGTCATGGGTGCCGCTGCCTGAGAATTTATACAAGCAGCTAGATTTCTCAACTAAATTCTTGAGATACAAAATCCCATT

  P  M  F  A  Y  P  L  Y  L  W  Y  R  S  P  G  K  T  G  S  H  F  N  P  D  S  S  L  F  K  P  N  E  R

     601 CCCCATGTTTGCCTACCCTCTCTACTTGTGGTATAGAAGTCCTGGAAAAACTGGATCTCATTTCAACCCAGATAGCAGTTTGTTCAAACCCAATGAGAGA

 D  L  V  I  T  S  T  V  C  W  A  A  M  V  A  F  L  L  Y  A  S  T  I  V  G  P  T  M  L  F  K  L  Y

     701 GATTTGGTCATCACTTCCACCGTTTGTTGGGCTGCAATGGTTGCTTTTCTCCTTTATGCTTCCACCATTGTTGGCCCAACCATGTTGTTCAAGCTCTACG

G  V  P  Y  L  I  F  V  V  W  L  D  T  V  T  Y  L  H  H  H  G  Y  D  K  K  L  P  W  Y  R  S  K  E  W

     801 GCGTGCCTTATTTGATATTCGTTGTGTGGTTGGACACGGTGACATATTTGCATCACCATGGTTACGACAAGAAGCTGCCATGGTACCGCAGCAAGGAATG

  S  Y  L  R  G  G  L  T  T  V  D  Q  D  Y  G  I  F  N  K  I  H  H  D  I  G  T  H  V  I  H  H  L  F

     901 GAGTTATTTAAGAGGAGGATTGACAACAGTAGATCAAGACTATGGAATATTCAACAAAATTCACCATGATATTGGCACCCATGTTATACACCACCTCTTC

 P  Q  I  P  H  Y  H  L  V  E  A  T  R  E  A  K  R  V  L  G  N  Y  Y  R  E  P  R  K  S  G  P  V  P

    1001 CCTCAGATCCCACACTACCATTTAGTGGAAGCGACGAGGGAGGCGAAGAGGGTGCTCGGAAATTACTACAGAGAGCCCAGAAAATCTGGACCAGTTCCAT

F  H  L  I  P  T  L  L  K  S  L  S  R  D  H  Y  V  S  D  N  G  D  I  V  Y  Y  Q  T  D  S  Q  L  F  S

    1101 TTCACTTGATTCCCACATTGTTGAAAAGTCTAAGCAGAGATCATTATGTCAGTGATAATGGAGACATAGTTTACTATCAAACTGATAGTCAGCTATTTTC

  S  K  E  I  *

    1201 GTCAAAAGAGATT**TAG**TGATGGGTTCTAAAATAATCAAATTGTATTAGATTTATTTCATGGTAGCTTTTTGGGGCCACATTGTTTTAATTAGAGAAATGA

    1301 TTGTGTGCCTCAAGAAGAATTTTAAATAATTGTATCGAGTTTTTCAGAATGCAATTTTGCTCCCCCTTGTTTT*T*TGTT*T*TATGATTATGTAATATCCCTT

    1401 TA*AATAAA*GTTTTTAGGTGATTGTGTGC*C*AGTTTTGTGGACTTTTGAAGATTACTTGTATTATT*T*AAAAAGAAAAGAAGAGGTTTGG***T***A*T*ATTTTGT*TAA*

    1501 *AAAAAAAAAAAAAAAAAAAAA*

K: *ShFAD3-1*mRNA

       1 *T*CCTCCACAAATGTGAACACGTATATGAATTCCAGTCTATATATACGCATACATGCCCCACTCACTCAC*G*C***C***T*A*TTCCATTCCTTTTGTAACTGAAAACT

                          M  A  V  S  S  G  A  D  A  E  H  H  G  H  A  Q  Y  E  H  L  G  K  R  A  A

     101 CTCGATTCGAAACCCCCTCCGGAAA**ATG**GCCGTCTCTTCCGGTGCCGACGCTGAGCACCACGGCCACGCCCAATACGAGCACCTCGGCAAGCGCGCCGCC

 D  K  F  D  P  A  A  P  P  P  F  K  I  A  D  I  R  A  A  I  P  P  H  C  W  V  K  D  P  L  R  S  L   ·

     201 GACAAATTCGACCCGGCCGCGCCTCCTCCGTTCAAGATCGCCGACATCCGCGCCGCCATCCCGCCGCATTGCTGGGTCAAGGACCCCCTCCGCTCCCTCA

S  Y  V  A  W  D  V  F  V  V  A  A  L  L  A  A  A  A  F  F  D  S  W  I  F  W  P  I  Y  W  A  A  Q  G ·

     301 GCTACGTCGCCTGGGATGTGTTCGTCGTCGCCGCGCTCCTCGCCGCCGCCGCCTTTTTCGACAGCTGGATCTTCTGGCCCATCTACTGGGCCGCCCAGGG

  T  M  F  W  A  L  F  V  L  G  H  D  C

                                                                                                     ·

     401 CACCATGTTTTGGGCCTTGTTCGTCCTCGGCCACGATTGGTAATACTAATCCCATTTTTAGTAATACTGAAATTACTACTAATTTTGATTTATTGCAGTG

G  H  G  S  F  S  D  N  T  T  L  N  N  V  V  G  H  V  L  H  S  S  I  L  V  P  Y  H  G  W

     501 GGCACGGGAGTTTTTCGGACAATACCACGCTGAATAACGTGGTGGGACATGTACTACATTCCTCAATTCTTGTACCTTATCATGGATGGTTAGTCTCCCA

                                                                R  I  S  H  R  T  H  H  Q  N  H  G   ·

     601 CTTTAAATTAATTTTTTTTAGAAATTTCAATGAGTAATATAGATCTGCATTTTACTACGTAGGCGAATAAGCCATCGAACACACCACCAGAATCATGGTC

H  V  E  N  D  E  S  W  V  P

     701 ATGTGGAGAACGACGAGTCATGGGTTCCGGTAATTAACATTTTTTAAATAAATTTGGAATTGGAAAATGGAAATGGATGTTTAAATAATTGCATTTGCAT

        L  T  E  N  L  Y  K  Q  L  D  F  S  T  K  F  L  R  Y  K  I  P  F  P  M  F  A  Y  P  L  Y  L

     801 TTTGCAGCTGACTGAGAATTTATACAAGCAGCTGGATTTCTCCACCAAATTCTTGAGATACAAAATCCCATTCCCCATGTTTGCCTACCCCCTATACTTG

     901 GTACTCTTTTTTCTCTCTCATACTAATATAAGTTATAAATTCACATTAATTTCCAAGACATAGGAAATTACTAAAAGATTGAATTGAATTGATTTAATTT

    W  Y  R  S  P  G  K  S  G  S  H  F  N  P  Y  S  S  L  F  K  P  N  E  R  D  L  V  I  T  S  T  I   ·

    1001 CAGTGGTATAGAAGCCCCGGAAAAAGTGGATCTCACTTCAACCCATATAGTAGTTTGTTCAAACCCAATGAGAGAGATTTGGTGATCACTTCCACCATAT

C  W  A  A  M  V  A  C  L  L  Y  A  S  T  I  V  G  P  T  M  L  F  K  L  Y  G  V  P  Y  L

    1101 GTTGGGCTGCAATGGTTGCTTGTCTCCTCTATGCTTCCACCATTGTTGGCCCAACCATGTTGTTCAAGCTCTACGGCGTTCCTTATTTGGTAACAACAAT

                                                                                         I  F  V  V

    1201 ATTAAATTATTACTACTACTTCATCAAACTCAATTACTATCATTTTATTAGTATATATATATATTCTATTTCAATATTTATGGGACAGATATTCGTTGTG

 W  L  D  T  V  T  Y  L  H  H  H  G  Y  D  K  K  L  P  W  Y  R  S  K

    1301 TGGTTGGACACGGTTACATATCTGCACCACCATGGTTACGACAAGAAACTCCCTTGGTACCGCAGCAAGGTAGTACTATTATTATTATGTACTAAATTAA

    1401 TTTTAATTTTAATTTCACTCCTGTCACTACCATATGTTCCCTGGTATTAATGTTGCATTGCCATTGTCAACACAAATATCTATGTTCGATCAACTATTAT

    1501 AGTATTATTTGTTTGGCTTAAAAATCCCACTTACCCATTAATTACTTTCTAATACTAGTGCTTTTTTAGTTTTCATTTCCCAAAGTATTTATATTTGCTT

    1601 TTTACCTAGATCTACCTTTATTAATTTTCCCTAATTCTTTTAATAACTTGGTTATGTCTCTTGGTTACAATTAGAAAGAGAATCGGGCCCAGTTGTCCAA

    1701 AACTCTGACTATTCACTGTATAGTTTAATTGTATGGTAGTATTAAAGTGGTCCAGTCAAAGAGTGACAATGTCCACTCAAAATATCATGAATTGGTTTAT

                                                                         E  W  S  Y  L  R  G  G  L   ·

    1801 TCTAAATCTAGATCGTTCATTCTGTTTCACAAGTAGTTGTAGTAATTTATGCCTTTTGTCTTGTAATTGCAGGAATGGAGTTATTTACGTGGAGGATTGA

T  T  V  D  Q  D  Y  G  I  F  N  K  I  H  H  D  I  G  T  H  V  V  H  H  L  F  P  Q  I  P  H  Y  H  L ·

    1901 CGACAGTAGATCAAGACTATGGAATATTCAACAAAATTCACCACGATATTGGCACCCATGTTGTTCACCACCTATTCCCTCAGATCCCACATTACCATTT

  V  E  A

    2001 AGTGGAGGCGGTGAGAATTAATAGATCTCTAATCATTAATTAATTATCTAATTAAACCCTAATCATGTGGCCAACACTGGCATTATTACCGTTTATTTGT

                                                               T  R  E  A  K  R  V  L  G  N  Y  Y  R ·

    2101 CCCTTTGTATGATTTTTTTATTTTAAATTAATTGATGAAATAAAATACAATGGATTTTGCAGACGAGGGAGGCGAAAAGGGTGCTCGGAAATTACTACAG

  E  P  R  K  S  G  A  V  P  F  H  L  V  P  T  L  L  K  S  L  S  R  D  H  Y  V  S  D  N  G  D  I  V

    2201 AGAGCCCAGAAAATCTGGAGCCGTTCCGTTTCACTTGGTTCCGACGTTGTTGAAAAGTCTAAGTAGAGATCATTATGTGAGTGACAATGGAGACATAGTT

 Y  Y  Q  T  D  G  E  L  F  S  S  K  E  I  *

    2301 TACTATCAAACAGATGGAGAACTATTTTCTTCTAAAGAGATT**TAG**TGATGGGCTCTAAACACCAAAGCATAGTAGATTTATTTAAACTAGCTTTTTGGGG

    2401 CCACATTATTTTCACAAGACAAATAATTGTGTGCAACAATAAGCCTTTTAAATAACTGTAGTAAGTTTTTTAGGTTGCAGTTTTGCTCTCCCTTGTTTTT

    2501 TTGTCTTTCCTTATGTTCAATTATGTAATGTTGCTTTAAATAAAGTTTACTTGTTGTAT*T*AT*C*

L: *ShFAD3-2*gene

       1 *T*CCTCCACAAATGTGAACACGTATATGAATTCCAGTCTATATATACGCATACATGCCCCACTCACTCAC*G*C***C***T*A*TTCCATTCCTTTTGTAACTGAAAACT

                          M  A  V  S  S  G  A  D  A  E  H  H  G  H  A  Q  Y  E  H  L  G  K  R  A  A

     101 CTCGATTCGAAACCCCCTCCGGAAA**ATG**GCCGTCTCTTCCGGTGCCGACGCTGAGCACCACGGCCACGCCCAATACGAGCACCTCGGCAAGCGCGCCGCC

 D  K  F  D  P  A  A  P  P  P  F  K  I  A  D  I  R  A  A  I  P  P  H  C  W  V  K  D  P  L  R  S  L   ·

     201 GACAAATTCGACCCGGCCGCGCCTCCTCCGTTCAAGATCGCCGACATCCGCGCCGCCATCCCGCCGCATTGCTGGGTCAAGGACCCCCTCCGCTCCCTCA

S  Y  V  A  W  D  V  F  V  V  A  A  L  L  A  A  A  A  F  F  D  S  W  I  F  W  P  I  Y  W  A  A  Q  G ·

     301 GCTACGTCGCCTGGGATGTGTTCGTCGTCGCCGCGCTCCTCGCCGCCGCCGCCTTTTTCGACAGCTGGATCTTCTGGCCCATCTACTGGGCCGCCCAGGG

  T  M  F  W  A  L  F  V  L  G  H  D  C  G  H  G  S  F  S  D  N  T  T  L  N  N  V  V  G  H  V  L  H

     401 CACCATGTTTTGGGCCTTGTTCGTCCTCGGCCACGATTGTGGGCACGGGAGTTTTTCGGACAATACCACGCTGAATAACGTGGTGGGACATGTACTACAT

 S  S  I  L  V  P  Y  H  G  W  R  I  S  H  R  T  H  H  Q  N  H  G  H  V  E  N  D  E  S  W  V  P  L   ·

     501 TCCTCAATTCTTGTACCTTATCATGGATGGCGAATAAGCCATCGAACACACCACCAGAATCATGGTCATGTGGAGAACGACGAGTCATGGGTTCCGCTGA

T  E  N  L  Y  K  Q  L  D  F  S  T  K  F  L  R  Y  K  I  P  F  P  M  F  A  Y  P  L  Y  L  W  Y  R  S ·

     601 CTGAGAATTTATACAAGCAGCTGGATTTCTCCACCAAATTCTTGAGATACAAAATCCCATTCCCCATGTTTGCCTACCCCCTATACTTGTGGTATAGAAG

  P  G  K  S  G  S  H  F  N  P  Y  S  S  L  F  K  P  N  E  R  D  L  V  I  T  S  T  I  C  W  A  A  M

     701 CCCCGGAAAAAGTGGATCTCACTTCAACCCATATAGTAGTTTGTTCAAACCCAATGAGAGAGATTTGGTGATCACTTCCACCATATGTTGGGCTGCAATG

 V  A  C  L  L  Y  A  S  T  I  V  G  P  T  M  L  F  K  L  Y  G  V  P  Y  L  I  F  V  V  W  L  D  T   ·

     801 GTTGCTTGTCTCCTCTATGCTTCCACCATTGTTGGCCCAACCATGTTGTTCAAGCTCTACGGCGTTCCTTATTTGATATTCGTTGTGTGGTTGGACACGG

V  T  Y  L  H  H  H  G  Y  D  K  K  L  P  W  Y  R  S  K  E  W  S  Y  L  R  G  G  L  T  T  V  D  Q  D ·

     901 TTACATATCTGCACCACCATGGTTACGACAAGAAACTCCCTTGGTACCGCAGCAAGGAATGGAGTTATTTACGTGGAGGATTGACGACAGTAGATCAAGA

  Y  G  I  F  N  K  I  H  H  D  I  G  T  H  V  V  H  H  L  F  P  Q  I  P  H  Y  H  L  V  E  A  T  R

    1001 CTATGGAATATTCAACAAAATTCACCACGATATTGGCACCCATGTTGTTCACCACCTATTCCCTCAGATCCCACATTACCATTTAGTGGAGGCGACGAGG

 E  A  K  R  V  L  G  N  Y  Y  R  E  P  R  K  S  G  A  V  P  F  H  L  V  P  T  L  L  K  S  L  S  R   ·

    1101 GAGGCGAAAAGGGTGCTCGGAAATTACTACAGAGAGCCCAGAAAATCTGGAGCCGTTCCGTTTCACTTGGTTCCGACGTTGTTGAAAAGTCTAAGTAGAG

D  H  Y  V  S  D  N  G  D  I  V  Y  Y  Q  T  D  G  E  L  F  S  S  K  E  I  *

    1201 ATCATTATGTGAGTGACAATGGAGACATAGTTTACTATCAAACAGATGGAGAACTATTTTCTTCTAAAGAGATT**TAG**TGATGGGCTCTAAACACCAAAGC

    1301 ATAGTAGATTTATTTAAACTAGCTTTTTGGGGCCACATTATTTTCACAAGACAAATAATTGTGTGCAACAATAAGCCTTTTAAATAACTGTAGTAAGTTT

    1401 TTTAGGTTGCAGTTTTGCTCTCCCTTGTTTTTTTGTCTTTCCTTATGTTCAATTATGTAATGTTGCTTTAAATAAAGTTTACTTGTTGTAT*T*AT*CAAAAA*

    1501 *AAAAAAAAAAAAAAAAAA*

M: *ShFAD3-2*mRNA

       1 *G*AGCCTCAACAAAATCAAGTTGGGATTCTTGAATTTGAG*GTGAGTATCACTTCCATTATCCATCCATTTTCTGCTGATTATACATACACATCTACATACA*

     101 *ATTCTGCAAGCCCCTCAACTCCACACACATTCTCTTTCCCACCCCAGTTACAGAAACTGATAAAGGTGCGATCTTTACCATTTTCAG*ATTT***G***GGGTGGTG

   M  A  S  W  V  L  S  G  C  G  L  K  P  L  P  R  I  Y  P  M  P  R  T  V  S  S  P  N  P  S  K  L  R ·

     201 AA**ATG**GCCAGTTGGGTTCTATCAGGATGTGGTCTAAAGCCACTTCCAAGAATCTACCCTATGCCAAGAACTGTCTCCAGTCCCAACCCCTCAAAGCTGAG

  I  S  T  A  D  F  S  S  D  S  S  S  L  C  S  V  G  R  G  R  N  W  G  L  N  V  S  A  P  L  R  F  Q

     301 AATTTCAACAGCAGATTTCTCCAGTGATTCCAGCTCATTGTGCAGTGTTGGGAGAGGGAGGAATTGGGGTTTGAATGTGAGTGCCCCACTGAGATTTCAG

 E  V  G  E  E  E  N  E  E  R  E  S  E  V  V  N  G  F  G  G  G  D  G  F  D  P  G  A  P  P  P  F  K   ·

     401 GAGGTGGGGGAGGAGGAGAATGAGGAGAGGGAGAGTGAGGTGGTAAATGGTTTTGGTGGTGGTGATGGATTTGACCCTGGTGCACCACCCCCATTCAAGC

L  A  D  I  R  A  A  I  P  K  H  C  W  V  K  N  P  W  K  S  M  S  Y  V  V  R  D  V  A  V  V  F  G  L ·

     501 TGGCTGATATTAGGGCAGCCATTCCTAAACATTGTTGGGTCAAGAATCCATGGAAGTCCATGAGCTATGTTGTGAGAGATGTTGCTGTGGTTTTTGGATT

  A  A  A  A  A  Y  L  N  N  W  A  V  W  P  L  Y  W  F  A  Q  G  T  M  F  W  A  L  F  V  L  G  H  D

     601 GGCTGCTGCTGCTGCCTATTTGAACAACTGGGCTGTGTGGCCTCTCTACTGGTTTGCTCAGGGAACTATGTTTTGGGCTCTGTTTGTTCTTGGCCATGAT

 C

     701 TGGTAAAGTTCTAATCTTTTTTTTTGTGGAGTGGTTTTGGTTGTTTTTTTCTAATTCTTGAATTGTTTTGGGCTCTGTTTTTGTGTTTGTGTGATTCTTT

     801 TTAATTATTCTTTTGGGCTCTGTTTGTTCTTGGCTATGATTGGTAAATTTGTAATCTTTTTTTGTGAGGTGGTTTGGTTTTGTGTTTGTTTGATTCCTTT

                           G  H  G  S  F  S  N  D  P  K  L  N  S  V  A  G  H  L  L  H  S  S  I  L  V ·

     901 TGATGATTCTTGAATTGTGTTTCAGTGGACATGGGAGCTTTTCCAATGATCCAAAGTTGAATAGTGTTGCTGGCCACCTGCTTCACTCTTCCATCCTTGT

  P  Y  H  G  W

    1001 TCCTTATCATGGATGGTAAGTTTAAACCTTAAAACCATAACTTTTTTATGTATGATATATTAATATTCATATGTATGTAATAAACCAAAATTGATGTTAA

                             R  I  S  H  R  T  H  H  Q  N  H  G  H  V  E  N  D  E  S  W  H  P

    1101 CAAATTGTGTAATGGTGAATGATTCAGGAGAATTAGTCACAGGACACACCATCAGAACCATGGACATGTTGAGAATGATGAATCTTGGCATCCAGTATGT

    1201 AGTTTTTCCACTTTTTTATGAAGATAATTTAGTTGCATTTTTCGCGTTTTTGTTGATAGAACGTGCAAGTTGAGATGTCTGTTTTGTTCATGTTGTTTGT

      L  S  E  K  I  Y  K  Q  L  D  F  V  T  K  K  L  R  F  T  L  P  F  P  M  L  A  Y  P  I  Y  L

    1301 TCCAGTTGTCTGAGAAGATATACAAGCAATTGGATTTTGTGACCAAGAAGTTGAGGTTCACATTGCCTTTTCCTATGTTGGCTTATCCCATCTATCTGGT

    1401 TGGCTTACTTTCTTCAGAATTCGAACTTTTGCATGTGAAATGTATGGTATTAGGCTCTCTTGATTGAAACCATTGCTCCTTTATGGTCCATGTAATGGCA

  W  S  R  S  P  G  K  K  G  S  H  F  H  P  D  S  D  L  F  V  P  N  E  R  K  D  V  I  T  S  T  V  C

    1501 GTGGAGCAGAAGTCCAGGGAAGAAAGGCTCTCATTTCCATCCAGACAGCGATTTGTTCGTTCCAAACGAGAGGAAAGATGTCATTACATCGACAGTTTGT

 W  T  A  M  V  A  I  L  A  G  L  S  F  V  M  G  P  L  Q  L  L  K  L  Y  G  I  P  Y  F

    1601 TGGACAGCAATGGTTGCAATTCTCGCAGGGCTCTCTTTCGTTATGGGTCCTCTTCAGTTGCTCAAACTCTATGGCATACCTTACTTCGTTAGTTCCTCCC

                                                                                     G  F  V  A  W   ·

    1701 GTTCCTCTCCTCTCAATAGCTCCACGTTTTCTTCAACGTTGTTTTACCTTGTATAACTTGAAAAAAAATGGGATTCTGATGTAGGGATTCGTGGCGTGGC

L  D  L  V  T  Y  L  H  H  H  G  H  E  D  K  L  P  W  Y  R  G  K

    1801 TTGATCTAGTTACCTACCTGCATCACCATGGCCACGAGGATAAGCTCCCTTGGTACCGAGGAAAGGTAAATTTAGCTTCTTGAATACACGAAACTCCACA

                                                        E  W  S  Y  L  R  G  G  L  T  T  L  D  R  D

    1901 AAGAATGTTGAAGTGTTCTTGTTTCAATCCCGGATTGATTTCGTTGGCGATGCAGGAGTGGAGTTATCTGAGAGGGGGGCTCACGACGCTTGATCGCGAC

 Y  G  W  I  N  N  I  H  H  D  I  G  T  H  V  I  H  H  L  F  P  Q  I  P  H  Y  H  L  I  E  A

    2001 TATGGATGGATAAACAACATCCACCACGACATAGGGACACATGTTATACATCACCTCTTCCCCCAAATCCCGCACTACCATTTGATAGAAGCAGTAAACA

                                                                                      T  E  A  A  K

    2101 TCCATCTTCTTCAAGAAAACTTGCTTCTTATATACAGTTTCACAGATTATTAATATTCACTGACCATTTGTGATTCTTGCAACAGACGGAAGCAGCAAAG

 P  V  L  G  K  Y  Y  K  E  P  Q  K  S  G  P  L  P  L  Y  L  L  G  V  L  A  K  S  M  K  K  D  H  Y   ·

    2201 CCGGTGCTCGGAAAATATTATAAGGAGCCTCAGAAATCAGGCCCTCTTCCATTATACTTGTTGGGAGTCCTCGCAAAGAGCATGAAAAAGGATCATTATG

V  S  D  T  G  D  I  V  Y  Y  Q  T  D  P  K  L  N  *

    2301 TGAGTGACACGGGTGACATAGTGTACTACCAGACCGATCCTAAGCTGAAC**TAA**ACCTCAACATGTCCATCGGTATATCTATAACAAATCGATAAATATGA

    2401 GACGTGGAGGCTGCTGGGCTCGGAACCTCTAAGGGGTCGTCTTCTAGCTGCAGAGCCGATTTTTTCGCCCTCCGGTGATGGAGACTTATATAGTTATTAC

    2501 AAGATTAAAAAAGATTGATGTGTATTTCTATAAAGGATGTAATGATTGTCTCTATAAAGGATGTGATTTGTT***G***AT*G*ATATGCTATTGTAGTAGTGATATG

    2601 TTTCCATGTAAACTAGTGTTTGGCATAT*G*AATACTATGGAATAATTATATT***C***AAGTAATGTGATTCATTAAGTTATT*C*

N: *ShFAD7a*gene

       1 *G*AGCCTCAACAAAATCAAGTTGGGATTCTTGAATTTGAG*GTGAGTATCACTTCCATTATCCATCCATTTTCTGCTGATTATACATACACATCTACATACA*

     101 *ATTCTGCAAGCCCCTCAACTCCACACACATTCTCTTTCCCACCCCAGTTACAGAAACTGATAAAGGTGCGATCTTTACCATTTTCAG*ATTT***G***GGGTGGTG

   M  A  S  W  V  L  S  G  C  G  L  K  P  L  P  R  I  Y  P  M  P  R  T  V  S  S  P  N  P  S  K  L  R ·

     201 AA**ATG**GCCAGTTGGGTTCTATCAGGATGTGGTCTAAAGCCACTTCCAAGAATCTACCCTATGCCAAGAACTGTCTCCAGTCCCAACCCCTCAAAGCTGAG

  I  S  T  A  D  F  S  S  D  S  S  S  L  C  S  V  G  R  G  R  N  W  G  L  N  V  S  A  P  L  R  F  Q

     301 AATTTCAACAGCAGATTTCTCCAGTGATTCCAGCTCATTGTGCAGTGTTGGGAGAGGGAGGAATTGGGGTTTGAATGTGAGTGCCCCACTGAGATTTCAG

 E  V  G  E  E  E  N  E  E  R  E  S  E  V  V  N  G  F  G  G  G  D  G  F  D  P  G  A  P  P  P  F  K   ·

     401 GAGGTGGGGGAGGAGGAGAATGAGGAGAGGGAGAGTGAGGTGGTAAATGGTTTTGGTGGTGGTGATGGATTTGACCCTGGTGCACCACCCCCATTCAAGC

L  A  D  I  R  A  A  I  P  K  H  C  W  V  K  N  P  W  K  S  M  S  Y  V  V  R  D  V  A  V  V  F  G  L ·

     501 TGGCTGATATTAGGGCAGCCATTCCTAAACATTGTTGGGTCAAGAATCCATGGAAGTCCATGAGCTATGTTGTGAGAGATGTTGCTGTGGTTTTTGGATT

  A  A  A  A  A  Y  L  N  N  W  A  V  W  P  L  Y  W  F  A  Q  G  T  M  F  W  A  L  F  V  L  G  H  D

     601 GGCTGCTGCTGCTGCCTATTTGAACAACTGGGCTGTGTGGCCTCTCTACTGGTTTGCTCAGGGAACTATGTTTTGGGCTCTGTTTGTTCTTGGCCATGAT

 C  G  H  G  S  F  S  N  D  P  K  L  N  S  V  A  G  H  L  L  H  S  S  I  L  V  P  Y  H  G  W  R  I   ·

     701 TGTGGACATGGGAGCTTTTCCAATGATCCAAAGTTGAATAGTGTTGCTGGCCACCTGCTTCACTCTTCCATCCTTGTTCCTTATCATGGATGGAGAATTA

S  H  R  T  H  H  Q  N  H  G  H  V  E  N  D  E  S  W  H  P  L  S  E  K  I  Y  K  Q  L  D  F  V  T  K ·

     801 GTCACAGGACACACCATCAGAACCATGGACATGTTGAGAATGATGAATCTTGGCATCCATTGTCTGAGAAGATATACAAGCAATTGGATTTTGTGACCAA

  K  L  R  F  T  L  P  F  P  M  L  A  Y  P  I  Y  L  W  S  R  S  P  G  K  K  G  S  H  F  H  P  D  S

     901 GAAGTTGAGGTTCACATTGCCTTTTCCTATGTTGGCTTATCCCATCTATCTGTGGAGCAGAAGTCCAGGGAAGAAAGGCTCTCATTTCCATCCAGACAGC

 D  L  F  V  P  N  E  R  K  D  V  I  T  S  T  V  C  W  T  A  M  V  A  I  L  A  G  L  S  F  V  M  G   ·

    1001 GATTTGTTCGTTCCAAACGAGAGGAAAGATGTCATTACATCGACAGTTTGTTGGACAGCAATGGTTGCAATTCTCGCAGGGCTCTCTTTCGTTATGGGTC

P  L  Q  L  L  K  L  Y  G  I  P  Y  F  G  F  V  A  W  L  D  L  V  T  Y  L  H  H  H  G  H  E  D  K  L ·

    1101 CTCTTCAGTTGCTCAAACTCTATGGCATACCTTACTTCGGATTCGTGGCGTGGCTTGATCTAGTTACCTACCTGCATCACCATGGCCACGAGGATAAGCT

  P  W  Y  R  G  K  E  W  S  Y  L  R  G  G  L  T  T  L  D  R  D  Y  G  W  I  N  N  I  H  H  D  I  G

    1201 CCCTTGGTACCGAGGAAAGGAGTGGAGTTATCTGAGAGGGGGGCTCACGACGCTTGATCGCGACTATGGATGGATAAACAACATCCACCACGACATAGGG

 T  H  V  I  H  H  L  F  P  Q  I  P  H  Y  H  L  I  E  A  T  E  A  A  K  P  V  L  G  K  Y  Y  K  E   ·

    1301 ACACATGTTATACATCACCTCTTCCCCCAAATCCCGCACTACCATTTGATAGAAGCAACGGAAGCAGCAAAGCCGGTGCTCGGAAAATATTATAAGGAGC

P  Q  K  S  G  P  L  P  L  Y  L  L  G  V  L  A  K  S  M  K  K  D  H  Y  V  S  D  T  G  D  I  V  Y  Y ·

    1401 CTCAGAAATCAGGCCCTCTTCCATTATACTTGTTGGGAGTCCTCGCAAAGAGCATGAAAAAGGATCATTATGTGAGTGACACGGGTGACATAGTGTACTA

  Q  T  D  P  K  L  N  *

    1501 CCAGACCGATCCTAAGCTGAAC**TAA**ACCTCAACATGTCCATCGGTATATCTATAACAAATCGATAAATATGAGACGTGGAGGCTGCTGGGCTCGGAACCT

    1601 CTAAGGGGTCGTCTTCTAGCTGCAGAGCCGATTTTTTCGCCCTCCGGTGATGGAGACTTATATAGTTATTACAAGATTAAAAAAGATTGATGTGTATTTC

    1701 TATAAAGGATGTAATGATTGTCTCTATAAAGGATGTGATTTGTT***G***AT*G*ATATGCTATTGTAGTAGTGATATGTTTCCATGTAAACTAGTGTTTGGCATAT

    1801 *G*AATACTATGGAATAATTATATT***C***AAGTAATGTGATTCATTAAGTTATT*CAAAAAAAAAAAAAAAAAAAAAAAAAAA*

O: *ShFAD7a*mRNA

       1 *G*AGCCTCAACAAAATCAAGTTGGGATTCTTGAATTTGAG*GTTTGTATCACTTCCATTATCCATCCATTTTCTGCTGATTATACATACACATCTACATACA*

     101 *TATTCTGCAAGCCCTCAACTCCACACACATTCTCTCTCCCACCCCAGTTACAGAAACTGATAAAGGTGCGATCTTTACCATTTTCAG*ATTT*G*GGGTGTTG

   M  A  S  W  V  L  S  G  C  G  L  K  P  L  P  R  I  Y  P  M  P  R  T  V  S  S  P  N  P  S  K  L  R ·

     201 AA**ATG**GCCAGTTGGGTTCTGTCAGGATGTGGTCTAAAGCCACTTCCAAGAATCTACCCTATGCCAAGAACTGTCTCCAGTCCCAACCCCTCAAAGCTGAG

  I  S  T  A  D  F  S  S  D  S  S  S  L  C  S  V  G  R  G  R  N  W  G  L  N  V  S  A  P  L  R  F  Q

     301 AATTTCAACAGCAGATTTCTCCAGTGATTCCAGCTCATTGTGCAGTGTTGGGAGAGGGAGGAATTGGGGTTTGAATGTGAGTGCCCCACTGAGATTTCAG

 E  V  G  E  E  E  N  E  E  R  E  S  E  V  V  N  G  F  G  G  G  D  G  F  D  P  G  A  P  P  P  F  K   ·

     401 GAGGTGGGGGAGGAGGAGAATGAGGAGAGGGAGAGTGAGGTGGTAAATGGTTTTGGTGGTGGTGATGGATTTGACCCTGGTGCACCACCCCCATTCAAGC

L  A  D  I  R  A  A  I  P  K  H  C  W  V  K  N  P  W  K  S  M  S  Y  V  V  R  D  V  A  V  V  F  G  L ·

     501 TGGCTGATATTAGGGCAGCCATTCCTAAACATTGTTGGGTCAAGAATCCATGGAAGTCCATGAGCTATGTTGTGAGAGATGTTGCTGTGGTTTTTGGATT

  A  A  A  A  A  Y  L  N  N  W  A  V  W  P  L  Y  W  F  A  Q  G  T  M  F  W  A  L  F  V  L  G  H  D

     601 GGCTGCTGCTGCTGCCTATTTGAACAACTGGGCTGTGTGGCCTCTCTACTGGTTTGCTCAGGGAACTATGTTTTGGGCTCTGTTTGTTCTTGGCCATGAT

 C

     701 TGGTAAATTTCTAATCTTTTTTTTTTGGGAGTGGTTTTGGTTGTGTTTTTCTAATTCTTGAATTGTTTTGGGCTCTGTTTTTGTGTTTGTGTGATTCTTT

     801 GTCATGATTCTTGAATTGTTTTGGGCTCTGTTTGTTCTTGGCCATCATTGGTAAATTTTGAATCTTTTTTTTGAAGGGTGGTTTTGTGTTTGTTTGTTCC

                              G  H  G  S  F  S  N  D  P  K  L  N  S  V  A  G  H  L  L  H  S  S  I  L ·

     901 TTCTGATGATTCTTGAATTGTGTTTCAGTGGACATGGGAGCTTTTCCAATGATCCAAAGCTGAATAGTGTTGCTGGTCACCTGCTTCACTCTTCCATCCT

  V  P  Y  H  G  W

    1001 TGTTCCTTATCATGGATGGTAAGTTTGAACCTTAAAATGATAGTAGTAACTTTTTATGTATAATATGCAATTGTATGTAAAAACCAAAATTGATGTGTAA

                             R  I  S  H  R  T  H  H  Q  N  H  G  H  V  E  N  D  E  S  W  H  P

    1101 CAAATTGTGTAATGGTGAATGATTCAGGAGAATTAGTCACAGGACACACCATCAGAACCATGGACATGTTGAGAATGATGAATCTTGGCATCCAGTATGT

    1201 AGTTTTTCCACTTTGTTATGAAGATAATTTAGTTGCATTTTTCGCGTTTTTGTTGATAGAAAGTGCAAGTTGAGATGTTTGTTTCTGCCATGTTGTTTGT

      L  S  E  K  I  Y  K  Q  L  D  F  V  T  K  K  L  R  F  T  L  P  F  P  M  L  A  Y  P  I  Y  L

    1301 TCCAGTTGTCTGAGAAGATATACAAGCAATTGGATTTTGTGACCAAGAAGTTGAGGTTCACATTGCCTTTTCCCATGTTGGCTTATCCCATCTATCTGGT

    1401 TGGCTTACTTTCTTCAAAATTCGAACTTTTTCGTATGAAATATATGGTATTAGGCTTTCTTGATTGAAACTATTGCTCCTTTATGGTCCATGTAATGGCA

  W  S  R  S  P  G  K  K  G  S  H  F  H  P  D  S  D  L  F  V  P  N  E  R  K  D  V  I  T  S  T  V  C

    1501 GTGGAGCAGAAGTCCAGGGAAGAAAGGCTCTCATTTCCATCCAGACAGCGATTTGTTCGTTCCCAACGAGAGGAAAGATGTCATTACATCGACAGTTTGT

 W  T  A  M  V  A  I  L  A  G  L  S  F  V  M  G  P  L  Q  L  L  K  L  Y  G  I  P  Y  F

    1601 TGGACAGCAATGGTTGCAATTCTCGCAGGGCTATCTTTCGTTATGGGTCCTCTTCAGTTGCTCAAACTCTATGGCATACCTTACTTCGTTAGTTCCTCCC

                                                                                    G  F  V  A  W  L ·

    1701 GTCCCTCTCCTCTCAATAGCTCCACGTTTTCTTCAACGTTGTTTTACCTTGTATAACTTGAAAAAAATGGGATTCTGATGTAGGGATTCGTGGCGTGGCT

  D  L  V  T  Y  L  H  H  H  G  H  E  D  K  L  P  W  Y  R  G  K

    1801 TGATCTAGTTACCTACCTGCATCACCATGGCCACGAGGATAAGCTCCCTTGGTACCGAGGAAAGGTAAATTTAGCTTCTTGAATACACGAAACTCCACAA

                                                       E  W  S  Y  L  R  G  G  L  T  T  L  D  R  D  Y ·

    1901 AGAATGTTGAAGTGTTCTTGTTTCAATCCCGGATTGATTTCGTTGGCGATGCAGGAGTGGAGTTACCTGAGAGGGGGGCTCACAACGCTTGATCGCGACT

·  G  W  I  N  N  I  H  H  D  I  G  T  H  V  I  H  H  L  F  P  Q  I  P  H  Y  H  L  I  E  A

    2001 ATGGATGGATAAACAACATCCACCACGACATAGGGACACACGTTATACATCACCTCTTCCCCCAAATCCCGCACTACCATTTGATAGAAGCAGTAAACAT

                                                                                        T  E  A  A   ·

    2101 CCATCTTCTTCAAGAAAACTCGCTTCTTATATACAGTTTCACAGATTATTACTAATATTCACTGACCATTTGTGATTCTTGCAACAGACGGAAGCAGCAA

K  P  V  L  G  K  Y  Y  K  E  P  Q  K  S  G  P  L  P  L  Y  L  L  G  V  L  A  K  S  M  K  K  D  H  Y ·

    2201 AGCCGGTGCTCGGAAAATATTATAAGGAGCCTCAGAAATCAGGCCCTCTTCCATTATACTTGTTGGGAGTCCTCGCAAAGAGCATGAAAAAGGATCATTA

  V  S  D  T  G  D  I  V  Y  Y  Q  T  D  P  K  L  N  *

    2301 TGTGAGTGACACGGGCGACATTGTGTACTACCAGACCGATCCTAAGCTGAAC**TAA**ACCTCAACATGTCCATCGGTATATCTATAACAAATCGATAAATAT

    2401 GAGACGTGGAGGCTGCTGGGCTCGGAATCAGCTCTCGGAACCTCTAAGGGGTTGTCTTCTAGCTGCAGACTCGATTTTTTCGCCCTCCGGTGGAGAGTTA

    2501 TATAGTTATTACAAGATTAAAAAAGATTGATGTGTATTTCTATAAAGATGTAATGACTGATTTCTTGATGCTATTGTAGTAGTGATATGTTTACATGTAA

    2601 ACTAGTGTTTGACATATGAATACTATGAAATAATTATATT***C***AGGTTATGTGATT*C*

P: *ShFAD7b*gene

       1 *G*AGCCTCAACAAAATCAAGTTGGGATTCTTGAATTTGAG*GTTTGTATCACTTCCATTATCCATCCATTTTCTGCTGATTATACATACACATCTACATACA*

     101 *TATTCTGCAAGCCCTCAACTCCACACACATTCTCTCTCCCACCCCAGTTACAGAAACTGATAAAGGTGCGATCTTTACCATTTTCAG*ATTT*G*GGGTGTTG

   M  A  S  W  V  L  S  G  C  G  L  K  P  L  P  R  I  Y  P  M  P  R  T  V  S  S  P  N  P  S  K  L  R ·

     201 AA**ATG**GCCAGTTGGGTTCTGTCAGGATGTGGTCTAAAGCCACTTCCAAGAATCTACCCTATGCCAAGAACTGTCTCCAGTCCCAACCCCTCAAAGCTGAG

  I  S  T  A  D  F  S  S  D  S  S  S  L  C  S  V  G  R  G  R  N  W  G  L  N  V  S  A  P  L  R  F  Q

     301 AATTTCAACAGCAGATTTCTCCAGTGATTCCAGCTCATTGTGCAGTGTTGGGAGAGGGAGGAATTGGGGTTTGAATGTGAGTGCCCCACTGAGATTTCAG

 E  V  G  E  E  E  N  E  E  R  E  S  E  V  V  N  G  F  G  G  G  D  G  F  D  P  G  A  P  P  P  F  K   ·

     401 GAGGTGGGGGAGGAGGAGAATGAGGAGAGGGAGAGTGAGGTGGTAAATGGTTTTGGTGGTGGTGATGGATTTGACCCTGGTGCACCACCCCCATTCAAGC

L  A  D  I  R  A  A  I  P  K  H  C  W  V  K  N  P  W  K  S  M  S  Y  V  V  R  D  V  A  V  V  F  G  L ·

     501 TGGCTGATATTAGGGCAGCCATTCCTAAACATTGTTGGGTCAAGAATCCATGGAAGTCCATGAGCTATGTTGTGAGAGATGTTGCTGTGGTTTTTGGATT

  A  A  A  A  A  Y  L  N  N  W  A  V  W  P  L  Y  W  F  A  Q  G  T  M  F  W  A  L  F  V  L  G  H  D

     601 GGCTGCTGCTGCTGCCTATTTGAACAACTGGGCTGTGTGGCCTCTCTACTGGTTTGCTCAGGGAACTATGTTTTGGGCTCTGTTTGTTCTTGGCCATGAT

 C  G  H  G  S  F  S  N  D  P  K  L  N  S  V  A  G  H  L  L  H  S  S  I  L  V  P  Y  H  G  W  R  I   ·

     701 TGTGGACATGGGAGCTTTTCCAATGATCCAAAGCTGAATAGTGTTGCTGGTCACCTGCTTCACTCTTCCATCCTTGTTCCTTATCATGGATGGAGAATTA

S  H  R  T  H  H  Q  N  H  G  H  V  E  N  D  E  S  W  H  P  L  S  E  K  I  Y  K  Q  L  D  F  V  T  K ·

     801 GTCACAGGACACACCATCAGAACCATGGACATGTTGAGAATGATGAATCTTGGCATCCATTGTCTGAGAAGATATACAAGCAATTGGATTTTGTGACCAA

  K  L  R  F  T  L  P  F  P  M  L  A  Y  P  I  Y  L  W  S  R  S  P  G  K  K  G  S  H  F  H  P  D  S

     901 GAAGTTGAGGTTCACATTGCCTTTTCCCATGTTGGCTTATCCCATCTATCTGTGGAGCAGAAGTCCAGGGAAGAAAGGCTCTCATTTCCATCCAGACAGC

 D  L  F  V  P  N  E  R  K  D  V  I  T  S  T  V  C  W  T  A  M  V  A  I  L  A  G  L  S  F  V  M  G   ·

    1001 GATTTGTTCGTTCCCAACGAGAGGAAAGATGTCATTACATCGACAGTTTGTTGGACAGCAATGGTTGCAATTCTCGCAGGGCTATCTTTCGTTATGGGTC

P  L  Q  L  L  K  L  Y  G  I  P  Y  F  G  F  V  A  W  L  D  L  V  T  Y  L  H  H  H  G  H  E  D  K  L ·

    1101 CTCTTCAGTTGCTCAAACTCTATGGCATACCTTACTTCGGATTCGTGGCGTGGCTTGATCTAGTTACCTACCTGCATCACCATGGCCACGAGGATAAGCT

  P  W  Y  R  G  K  E  W  S  Y  L  R  G  G  L  T  T  L  D  R  D  Y  G  W  I  N  N  I  H  H  D  I  G

    1201 CCCTTGGTACCGAGGAAAGGAGTGGAGTTACCTGAGAGGGGGGCTCACAACGCTTGATCGCGACTATGGATGGATAAACAACATCCACCACGACATAGGG

 T  H  V  I  H  H  L  F  P  Q  I  P  H  Y  H  L  I  E  A  T  E  A  A  K  P  V  L  G  K  Y  Y  K  E   ·

    1301 ACACACGTTATACATCACCTCTTCCCCCAAATCCCGCACTACCATTTGATAGAAGCAACGGAAGCAGCAAAGCCGGTGCTCGGAAAATATTATAAGGAGC

P  Q  K  S  G  P  L  P  L  Y  L  L  G  V  L  A  K  S  M  K  K  D  H  Y  V  S  D  T  G  D  I  V  Y  Y ·

    1401 CTCAGAAATCAGGCCCTCTTCCATTATACTTGTTGGGAGTCCTCGCAAAGAGCATGAAAAAGGATCATTATGTGAGTGACACGGGCGACATTGTGTACTA

  Q  T  D  P  K  L  N  *

    1501 CCAGACCGATCCTAAGCTGAAC**TAA**ACCTCAACATGTCCATCGGTATATCTATAACAAATCGATAAATATGAGACGTGGAGGCTGCTGGGCTCGGAATCA

    1601 GCTCTCGGAACCTCTAAGGGGTTGTCTTCTAGCTGCAGACTCGATTTTTTCGCCCTCCGGTGGAGAGTTATATAGTTATTACAAGATTAAAAAAGATTGA

    1701 TGTGTATTTCTATAAAGATGTAATGACTGATTTCTTGATGCTATTGTAGTAGTGATATGTTTACATGTAAACTAGTGTTTGACATATGAATACTATGAAA

    1801 TAATTATATT***C***AGGTTATGTGATT*CAAAAAAAAAAAAAAAAAAAAAA*

Q: *ShFAD7b*mRNA

       1 *A*GCCACAAATTGCTTCCACTTGCATACTTCAGAGAAGTGGAGCCCTTTATCAACCTCTCAACTCTCTTCCACCCAAATCAACTTGCGCTCTCTTTCCATA

     101 TACAAACAACATTCCCCCAACTTCCTC*A*AG*T*TCACAACTTTCTCCACAGGGAGAGAGCCC*C*TCTT*A*AAAAGGTGAAATCTTTATTGGATTGCCTCTCTCT

        M  A  S  F  V  I  S  G  C  G  L  K  P  L  P  R  I  Y  P  K  P  R  S  V  Q  N  S  F  S  T  S

     201 *C*TCTCTC**ATG**GCGAGTTTTGTCATATCAGGTTGTGGCTTGAAGCCGCTTCCAAGAATCTATCCTAAACCAAGGAGTGTCCAAAATTCTTTCTCCACCTCT

 N  L  R  I  S  R  P  N  Q  F  S  S  S  S  I  G  I  N  Q  K  R  N  W  G  L  G  V  S  A  P  L  R  I   ·

     301 AATTTAAGAATTTCAAGACCCAACCAGTTTTCTTCTTCGTCAATTGGGATTAATCAGAAACGGAATTGGGGTTTGGGAGTGAGTGCGCCATTGAGGATTC

Q  P  L  E  E  E  N  E  E  F  D  P  A  A  P  P  P  F  K  L  S  D  I  K  A  A  I  P  K  H  C  W  V  K ·

     401 AGCCACTGGAAGAAGAGAACGAGGAATTCGACCCGGCGGCGCCGCCTCCGTTCAAGTTGTCTGACATAAAGGCAGCCATTCCTAAGCATTGTTGGGTGAA

  D  P  W  R  S  V  G  Y  V  V  R  D  V  V  A  V  L  G  M  A  A  A  A  A  Y  F  N  S  W  I  V  W  P

     501 GGACCCTTGGAGGTCTGTGGGTTATGTGGTGAGAGATGTGGTGGCTGTTTTGGGGATGGCGGCCGCCGCCGCCTATTTCAACAGTTGGATTGTTTGGCCA

 L  Y  W  F  A  Q  S  T  M  F  W  A  L  F  V  L  G  H  D  C

     601 TTGTACTGGTTTGCTCAGAGCACCATGTTCTGGGCTCTCTTTGTTCTTGGTCATGACTGGTAATGTTTTTGGGAGATTGCTTTTGTGTTTTTTGGTGGGA

                                                    G  H  G  S  F  S  N  N  P  K  L  N  S  V  F  G   ·

     701 GTTTTGTGTTAAAGATTTGATCTTGATGTGCCTTGAATTGATTTATGCAGTGGCCATGGAAGCTTTTCTAACAATCCCAAGTTGAATAGTGTGTTTGGCC

H  F  L  H  S  S  I  L  V  P  Y  H  G  W

     801 ATTTCCTTCACTCTTCAATTTTGGTGCCCTACCATGGATGGTATGTTGATGTCTATTTGTCATTGCATTGGTAATGTTTATGTTTAGTAACTAAAATTGA

                                         R  I  S  H  R  T  H  H  Q  N  H  G  H  V  E  N  D  E  S  W

     901 ATCATGTTGGTTGTTGATTGTTATGGTTGATTGATGTAGGAGAATTAGTCACAGAACTCATCATCAGAACCATGGGCATGTTGAGAATGATGAATCTTGG

 H  P

    1001 CACCCTGTAAGTTTGTTTCTGTGGATTCGAAAACCTTGATTCTTTGTTGATGCTGTTGTATGGATGTCTCACAAAGCCAGAATTTATAATCTTGATTGCA

  M  P  E  K  I  Y  N  S  L  D  S  M  A  K  K  L  R  F  T  L  P  F  P  M  L  A  Y  P  I  Y  L

    1101 GATGCCTGAGAAGATTTACAATAGCTTGGACAGTATGGCCAAGAAATTGAGGTTTACATTGCCTTTCCCTATGTTGGCATATCCTATTTATCTGGTGAGC

                                                                          W  T  R  S  P  G  K  K  G

    1201 ACCTTACTTCTTCGATGTGTTTTTTGCCCTCTGAATTCGTTTTAATGACGTGTTTTTTGTTGTTTGATCGCAGTGGACACGAAGTCCTGGAAAAAAAGGC

 S  H  Y  H  P  D  S  D  L  F  V  P  A  E  R  K  D  V  I  T  S  T  V  C  W  T  A  M  A  A  L  L  V   ·

    1301 TCTCATTACCACCCGGACAGTGATTTGTTTGTCCCGGCTGAGAGGAAAGATGTTATCACCTCAACAGTTTGTTGGACTGCAATGGCTGCCTTGCTTGTAG

G  L  S  F  V  M  G  P  I  Q  L  L  K  L  Y  G  I  P  Y  L

    1401 GACTATCTTTTGTCATGGGTCCGATCCAGCTTCTCAAACTATACGGCATTCCTTACTTGGTCAGTCATTTCAATCCCGTTGATTATACGACTGTGAAATG

    1501 TGGCTTTTCTACTTGTCAAAGATTTGGGCAGTTTTAGTATAAGTTGGTCATGAATTATCCTATCTCGGACTTGTTGGTAAAACTTAGAATTTGACTTGTG

       G  F  V  A  W  L  D  T  V  T  Y  L  H  H  H  G  H  E  D  K  L  P  W  Y  R  G  K

    1601 CTTCAGGGCTTTGTAGCGTGGCTTGATACCGTGACTTATTTGCATCATCATGGCCATGAAGATAAGCTCCCTTGGTACCGCGGAAAGGTAAAACTTAACT

    1701 CATAATACTCCCTCCGTTTCTTAAAAAAACTCTTTCCATTTTGGTCCATTCCATAAAAATAGAAACTTTCCATTTTAGGAAACTTCTCTCTCTCTCTCTC

    1801 TCTCTCTCTCTATATATATATATATATATGAGATGAGACCTATTTTAACACACTTTTGCATTAAAACCCTTGCTGTTTAAAAAATTCCTATTTTTACGAA

                                                                                                  E

    1901 ACGAGGGAGTATGAAAATCCGGGAAGAGAGAATGTTCTCACAACTTGTACTTGCAGTTATTTTTTTATAATGTCTCTATATTCTTTGATAAAACCAGGAA

 W  S  Y  L  R  G  G  L  T  T  L  D  R  D  Y  G  L  I  N  N  I  H  H  D  I  G  T  H  V  I  H  H  L   ·

    2001 TGGAGTTATCTGAGAGGCGGGCTCACGACACTTGATCGCGACTACGGATTGATTAACAACATCCATCATGATATAGGAACTCATGTCATACACCATCTCT

F  P  Q  I  P  H  Y  N  L  I  E  A

    2101 TCCCCCAAATCCCACACTACAACCTAATAGAAGCTGTAAGTTTTTTTTCCATATTTAAAGGCATTACTTTGCCTCAGACAGTTGAGTTGGTTTTACATGT

                                      T  E  A  A  K  G  V  L  G  K  Y  Y  R  E  P  K  K  S  G  P  L

    2201 TTTTGACTCGTGAGCTTGGCCGATTCTTGCAATGCAGACGGAAGCAGCAAAGGGGGTGTTGGGAAAGTACTACAGAGAGCCGAAGAAGTCAGGCCCTCTA

 P  L  H  L  L  G  D  L  V  R  S  L  K  K  D  H  Y  V  S  D  T  G  D  V  V  Y  Y  Q  T  D  P  Q  L   ·

    2301 CCACTCCACTTGCTGGGAGACCTCGTGCGAAGCTTGAAGAAGGATCACTATGTGAGCGACACAGGCGACGTCGTCTATTATCAGACAGATCCTCAACTCA

N  G  G  Q  K  S  *

    2401 ATGGGGGTCAAAAATCT**TAA**GCTGTGATGAAATTTTTTATGGATTCTTTACCAGATGCTAATTCTTTGAAGCTTTTTGTTATGACACTGGTCTGAGCTCA

    2501 TACTGCAAACATTCTGACCTTTGGAA*C*ATATAGATAGTGTTTTTTTTTTTTTTTTTTTGCAACACAATTAATTTTTGCTTCATTTCTTTAATGGAGCAAT

    2601 *C*ATATATTAGTAATAGATTGACTGCATTTGTCAAAGAATATAAATTATAGTGTATTTGATTT*T*TATTTT*G*AGTTGTAGTGTTATGGCATTGAACACTGTA

    2701 TATTTGATTGAAATTG*C*

R: *ShFAD8*gene

       1 *A*GCCACAAATTGCTTCCACTTGCATACTTCAGAGAAGTGGAGCCCTTTATCAACCTCTCAACTCTCTTCCACCCAAATCAACTTGCGCTCTCTTTCCATA

     101 TACAAACAACATTCCCCCAACTTCCTC*A*AG*T*TCACAACTTTCTCCACAGGGAGAGAGCCC*C*TCTT*A*AAAAGGTGAAATCTTTATTGGATTGCCTCTCTCT

        M  A  S  F  V  I  S  G  C  G  L  K  P  L  P  R  I  Y  P  K  P  R  S  V  Q  N  S  F  S  T  S

     201 *C*TCTCTC***A*TG**GCGAGTTTTGTCATATCAGGTTGTGGCTTGAAGCCGCTTCCAAGAATCTATCCTAAACCAAGGAGTGTCCAAAATTCTTTCTCCACCTCT

 N  L  R  I  S  R  P  N  Q  F  S  S  S  S  I  G  I  N  Q  K  R  N  W  G  L  G  V  S  A  P  L  R  I   ·

     301 AATTTAAGAATTTCAAGACCCAACCAGTTTTCTTCTTCGTCAATTGGGATTAATCAGAAACGGAATTGGGGTTTGGGAGTGAGTGCGCCATTGAGGATTC

Q  P  L  E  E  E  N  E  E  F  D  P  A  A  P  P  P  F  K  L  S  D  I  K  A  A  I  P  K  H  C  W  V  K ·

     401 AGCCACTGGAAGAAGAGAACGAGGAATTCGACCCGGCGGCGCCGCCTCCGTTCAAGTTGTCTGACATAAAGGCAGCCATTCCTAAGCATTGTTGGGTGAA

  D  P  W  R  S  V  G  Y  V  V  R  D  V  V  A  V  L  G  M  A  A  A  A  A  Y  F  N  S  W  I  V  W  P

     501 GGACCCTTGGAGGTCTGTGGGTTATGTGGTGAGAGATGTGGTGGCTGTTTTGGGGATGGCGGCCGCCGCCGCCTATTTCAACAGTTGGATTGTTTGGCCA

 L  Y  W  F  A  Q  S  T  M  F  W  A  L  F  V  L  G  H  D  C  G  H  G  S  F  S  N  N  P  K  L  N  S   ·

     601 TTGTACTGGTTTGCTCAGAGCACCATGTTCTGGGCTCTCTTTGTTCTTGGTCATGACTGTGGCCATGGAAGCTTTTCTAACAATCCCAAGTTGAATAGTG

V  F  G  H  F  L  H  S  S  I  L  V  P  Y  H  G  W  R  I  S  H  R  T  H  H  Q  N  H  G  H  V  E  N  D ·

     701 TGTTTGGCCATTTCCTTCACTCTTCAATTTTGGTGCCCTACCATGGATGGAGAATTAGTCACAGAACTCATCATCAGAACCATGGGCATGTTGAGAATGA

  E  S  W  H  P  M  P  E  K  I  Y  N  S  L  D  S  M  A  K  K  L  R  F  T  L  P  F  P  M  L  A  Y  P

     801 TGAATCTTGGCACCCTATGCCTGAGAAGATTTACAATAGCTTGGACAGTATGGCCAAGAAATTGAGGTTTACATTGCCTTTCCCTATGTTGGCATATCCT

 I  Y  L  W  T  R  S  P  G  K  K  G  S  H  Y  H  P  D  S  D  L  F  V  P  A  E  R  K  D  V  I  T  S   ·

     901 ATTTATCTGTGGACACGAAGTCCTGGAAAAAAAGGCTCTCATTACCACCCGGACAGTGATTTGTTTGTCCCGGCTGAGAGGAAAGATGTTATCACCTCAA

T  V  C  W  T  A  M  A  A  L  L  V  G  L  S  F  V  M  G  P  I  Q  L  L  K  L  Y  G  I  P  Y  L  G  F ·

    1001 CAGTTTGTTGGACTGCAATGGCTGCCTTGCTTGTAGGACTATCTTTTGTCATGGGTCCGATCCAGCTTCTCAAACTATACGGCATTCCTTACTTGGGCTT

  V  A  W  L  D  T  V  T  Y  L  H  H  H  G  H  E  D  K  L  P  W  Y  R  G  K  E  W  S  Y  L  R  G  G

    1101 TGTAGCGTGGCTTGATACCGTGACTTATTTGCATCATCATGGCCATGAAGATAAGCTCCCTTGGTACCGCGGAAAGGAATGGAGTTATCTGAGAGGCGGG

 L  T  T  L  D  R  D  Y  G  L  I  N  N  I  H  H  D  I  G  T  H  V  I  H  H  L  F  P  Q  I  P  H  Y   ·

    1201 CTCACGACACTTGATCGCGACTACGGATTGATTAACAACATCCATCATGATATAGGAACTCATGTCATACACCATCTCTTCCCCCAAATCCCACACTACA

N  L  I  E  A  T  E  A  A  K  G  V  L  G  K  Y  Y  R  E  P  K  K  S  G  P  L  P  L  H  L  L  G  D  L ·

    1301 ACCTAATAGAAGCTACGGAAGCAGCAAAGGGGGTGTTGGGAAAGTACTACAGAGAGCCGAAGAAGTCAGGCCCTCTACCACTCCACTTGCTGGGAGACCT

  V  R  S  L  K  K  D  H  Y  V  S  D  T  G  D  V  V  Y  Y  Q  T  D  P  Q  L  N  G  G  Q  K  S  *

    1401 CGTGCGAAGCTTGAAGAAGGATCACTATGTGAGCGACACAGGCGACGTCGTCTATTATCAGACAGATCCTCAACTCAATGGGGGTCAAAAATCT**TAA**GCT

    1501 GTGATGAAATTTTTTATGGATTCTTTACCAGATGCTAATTCTTTGAAGCTTTTTGTTATGACACTGGTCTGAGCTCATACTGCAAACATTCTGACCTTTG

    1601 GAA*C*ATATAGATAGTGTTTTTTTTTTTTTTTTTTTGCAACACAATTAATTTTTGCTTCATTTCTTTAATGGAGCAAT*C*ATATATTAGTAATAGATTGACT

    1701 GCATTTGTCAAAGAATATAAATTATAGTGTATTTGATTT*T*TATTTT*G*AGTTGTAGTGTTATGGCATTGAACACTGTATATTTGATTGAAATTG*CAAAAAA*

    1801 *AAAAAAAAAAAAAAAAAAAAAAAAAAAAAAAAAAAAAAA*

S: *ShFAD8*mRNA

**S1 Fig. Gene and protein sequences of *ω-3 FAD* gene family from chia and perilla (A-S).** The start codon (ATG), and the stop codon (TAA, TAG and TGA) are in underlined in bold face. Alternative transcription sites, and poly(A) tailing sites are underlined and italicized, and the major types are in bold face. The introns, and typical and non-typical poly(A) signals are underlined. The purine-stretches (> 20 bp) and pyrimidine-stretches (> 20 bp) are highlighted by gray background.
