# Supplementary material for: Omega-3 fatty acid desaturase gene family from two ω-3 sources, Salvia hispanica and Perilla frutescens: Cloning, characterization and expression
Source: PLoS One. 2018 Jan 19;13(1):e0191432. doi: 10.1371/journal.pone.0191432 (PMC5774782; doi:10.1371/journal.pone.0191432)
Supplement: S2 Fig — PfrFAD3-1 mRNA (NCBI accession no AF047039.1), PfrFAD3-2 mRNA (KX228917.1), PfrFAD7-1 mRNA (U59477.1) and PfrFAD7-2 mRNA (KP070824.1). (DOCX) [file pone.0191432.s006.docx]

PfFAD3b mRNA      (1) ----------------------------------------------------------------------------------------------------

PfrFAD3-1 mRNA    (1) ----------------------------------------------------------------------------------------------------

PfFAD3a mRNA      (1) ----------------------------------------------------------------------------------------------------

PfrFAD3-2 mRNA    (1) ----------------------------------------------------------------------------------------------------

PfFAD7a mRNA      (1) --GGAGGCATAGGTGGTGGAGTCGGAAA-GAGAGAAGAAATAGAGGCTGAAAATTGTGTATTTAAATGAATCACATTCTG-CTCGGG------TTATAAG

PfFAD7b mRNA      (1) --GGAGGCATAGGTGGTGGAGTCGGAAA-GAGAGAAGAGATAGAGGCTGAAAATTGTGTATTTAAATGAATCACATTCTG-CTCGGG------TTATAAG

PfrFAD7-1 mRNA    (1) --------------------------------------------------------------------------------------------------GG

PfFAD8a mRNA      (1) GGGGAAAGAGAGAAGAAAGAGAGGCAGATGCGAATTGCGATGGCTGTGGCAGTGTCTATATATATATCACCCATCTCCTCTCTCTCTCTCTCTCTCTCCT

PfFAD8b mRNA      (1) GGGGAAAGAGAGAAGAAAGAGAGGCAGATGCGAATTGCGATGGCTGTAGCACTGTGTGTATATATATCAGCCATCTCCTCTCTCTCT------CTCTCCT

PfrFAD7-2 mRNA    (1) ----------------------------------------------------------------------------------------------------

PfFAD3b mRNA      (1) ----------------------------------------------------------------------------------------------------

PfrFAD3-1 mRNA    (1) ----------------------------------------------------------------------------------------------------

PfFAD3a mRNA      (1) ----------------------------------------------------------------------------------------------------

PfrFAD3-2 mRNA    (1) ----------------------------------------------------------------------------------------------------

PfFAD7a mRNA     (91) CC-TCAAGAAAATCCAAATCAACAAGTTGGGATTCTTGGAAGCCATAA------ATTTGAGGAGA-----GAATCCCTTGTATTATCTACTA---ACTTA

PfFAD7b mRNA     (91) CC-TCAAGAAAATCCAAATCAACAAGTTGGGATTCTTGGAAGCCATAA------ATTTGAGGAGA-----GAATCCCTTGTATTATCTACTA---ACTTA

PfrFAD7-1 mRNA    (3) CACGAGAGAAAATCCAAATCAACAAGTTGGGATTCTTGGAAGCCATAA------ATTTGAGGAGA-----GAATCCCTTGTATTATCTACTA---ACTTA

PfFAD8a mRNA    (101) CCCTAAAAACAACCCACAACGCCAACTTGTGTCCACTTGCGGCCCTAACGTGAGAGGAGAGGAGAGGAGAGGAGCCCTTTTATCATCAACTGCTCACCTC

PfFAD8b mRNA     (95) CCCTAGAAAAAACCCACAACACCAACTTGTGTCCACTTGCGGCCCTAACGTGAGAGGAGAGGAGA-----GGAGCCCTTTTATCATCAACTACTCACCTC

PfrFAD7-2 mRNA    (1) ----------------------------------------------------------------------------------------------------

PfFAD3b mRNA      (1) ----------------------------------------------------------------------------------------------------

PfrFAD3-1 mRNA    (1) ----------------------------------------------------------------------------------------------------

PfFAD3a mRNA      (1) ----------------------------------------------------------------------------------------------------

PfrFAD3-2 mRNA    (1) ----------------------------------------------------------------------------------------------------

PfFAD7a mRNA    (176) AGCATATACATACATATTCTGCAAACC----CCTCAA------------GTCCACACACTATTCTCT----CTCTTTCTCTCTCAT-----TCTAGTGAG

PfFAD7b mRNA    (176) AGCATATACATACATATTCTGCAAACC----CCTCAA------------GTCCACACACTATTCTCT----CTCTTTCTCTCTCAT-----TCTAGTGAG

PfrFAD7-1 mRNA   (89) AGCATATACATACATATTCTGCAAACC----CCTCAA------------GTCCACACACTATTCTCT----CTCTTTCTCTCTCAT-----TCTAGTGAG

PfFAD8a mRNA    (201) TCAATCCAGCGGAATCATCTGCATTCCTGCACATAAAATACGTGTATACATACAAGCAATATTCTGCCAAGCCCTTAAGTCCACAAGTTTCTCTAGAGAG

PfFAD8b mRNA    (190) TCAATCCAGCGGAATCATCTGCATTCCTGCACATAAA-TACGTGTGTACATACAAACAATATTCTGCCAAGCCCTTAAGTCCACAAGTTTCTCTAGAGAG

PfrFAD7-2 mRNA    (1) ----------------------------------------------------------------------------------------------------

PfFAD3b mRNA      (1) ----------------------------------------------------------------------------------------------------

PfrFAD3-1 mRNA    (1) ------------------------------------------------------------------CGGCACGAGCTCCTCCACAAATCTCAGTTACACA

PfFAD3a mRNA      (1) ----------------------------------------------------------------------------------------------------

PfrFAD3-2 mRNA    (1) ----------------------------------------------------------------------------------------------------

PfFAD7a mRNA    (251) AGA-------ACCCCAAT--AAAGGTGAAATCTTGATTACTTTCAGAGTTGGGGC-------------------TCTCTCTCTGAAATGGCGAGTTGGGT

PfFAD7b mRNA    (251) AGA-------ACCCCAAT--AAAGGTGAAATCTTGATTACTTTCAGAGTTGGGGC-------------------TCTCTCTCTGAAATGGCGAGTTGGGT

PfrFAD7-1 mRNA  (164) AGA-------ACCCCAAT--AAAGGTGAAATCTTGATTACTTTCAGAGTTGGGGC-------------------TCTCTCTCTGAAATGGCGAGTTGGGT

PfFAD8a mRNA    (301) AGAAAGAGAGAGCTTTTTTAAAAGGTGAATTCTTGGTTGGGTTCTGAATTGGGCCGCCTCTCT------TTCTTTCCCTCTCTGTAATGGCGAGTTTCGT

PfFAD8b mRNA    (289) AGAAAGAGGGAGCTTTTTTAAAAGGTGAATTCTTGGTTGGGTTCTGAATTGGGCCGCCTCTCTCTCTCTTTCTTTCCCTCTCTGTAATGGCGAGTTTCGT

PfrFAD7-2 mRNA    (1) --------------------------------------------------------------------------------------ATGGCGAGTTTCGT

PfFAD3b mRNA      (1) ------------------------------------------------------------------------GTTCTAAC---TGAAACTCGCTAGTTTA

PfrFAD3-1 mRNA   (35) CACATCGCCCCCCTCTCTCTCTAAACACTTTCCTCTCTCTCTATATATATGCCAACCTCATGTATTCCATTTGTTCTAAC---TGAAACTCGCTAGTTTA

PfFAD3a mRNA      (1) ------------------------------------------------------------------------GTTCTAAC---TGAAACTCGCTAGTTTA

PfrFAD3-2 mRNA    (1) ----------------------------------------------------------------------------------------------------

PfFAD7a mRNA    (323) GTTATCAGAATGTGGTTTAAGGCCACTTCCAAGAATATACCCCAAGCCAAGAACTGGCCA-GTATCTCTCCAATTCCAACCCCTCAAAGTTGAGACTCTC

PfFAD7b mRNA    (323) GTTATCAGAATGTGGTTTAAGGCCACTTCCAAGAATATACCCCAAGCCAAGAACTGGCCA-GTATCTCTCCAATTCCAACCCCTCAAAGTTGAGACTCTC

PfrFAD7-1 mRNA  (236) GTTATCAGAATGTGGTTTAAGGCCACTTCCAAGAATATACCCCAAGCCAAGAACTGGCCA-GTATCTCTCCAATTCCAACCCCTCAAAGTTGAGACTCTC

PfFAD8a mRNA    (395) TATATCAGAATGTGGCTTGAAGCCACTTCCAAGAATCTATCCCAAACCAAGAGCTGCCCA-GCCTCTCTCGAGTTCTAATC--TGAGATTTTCAAGAACA

PfFAD8b mRNA    (389) TATATCAGAATGTGGCTTGAAGCCACTTCCAAGAATCTATCCCAAACCGAGAGCTGCCCA-GCCTCTCTCGAGTTCTAATC--TGAGATTTTCAAGAACA

PfrFAD7-2 mRNA   (15) TATATCAGAATGTGGCTTGAAGCCACTTCCAAGAATCTATCCCAAACCGAGAGCTGCCCA-GCCTCTCTCGAGTTCTAATC--TGAGATTTTCAAGAACA

PfFAD3b mRNA     (26) TTGATTCAAACCCTCCCCAAAAGAATGG--CCGTTTCTTCCGGTGCCCGCCTCTCGAAGAGTGGCGCTGATGGAGAGGTTTTCGACGGCCAA--CAACAA

PfrFAD3-1 mRNA  (132) TTGATTCAAACCCTCCCCAAAAGAATGG--CCGTTTCTTCCGGTGCCCGCCTCTCGAAGAGTGGCGCTGATGGAGAGGTTTTCGACGGCCAA--CAACAA

PfFAD3a mRNA     (26) TTGATTCAAACCCTCCCCAAAAGAATGG--CCGTTTCTTCCGGTGCCCGCCTCTCGAAGAGTGGCGCTGATGGAGAGGTTTTCGACGGCCAA--CAACAA

PfrFAD3-2 mRNA    (1) --------------------------ATGGCCGTTTCTTCCGGTGCCCGCCTCTCGAAGAGTGGCGCTGATGGAGAGGTTTTCGACGGCCAA--CAACAA

PfFAD7a mRNA    (422) ---AAGAACAGGTTTTTCAAGTGATTCC--TCATTCAGTTTGGTTGGTAGAGAGAGAAACTGGGGTTTGAAGGTGAG-TGCTCCACTGAGATTTCAGGAG

PfFAD7b mRNA    (422) ---AAGAACAGGTTTTTCAAGTGATTCC--TCATTCAGTTTGGTTGGTAGAGAGAGAAACTGGGGTTTGAAGGTGAG-TGCTCCACTGAGATTTCAGGAG

PfrFAD7-1 mRNA  (335) ---AAGAACAGGTTTTTCAAGTGATTCC--TCATTCAGTTTGGTTGGTAGAGAGAGAAACTGGGGTTTGAAGGTGAG-TGCTCCACTGAGATTTCAGGAG

PfFAD8a mRNA    (492) ---AATCAACGGTTTAATTCTTCATTCTGTTCATCAACTGGGATTATTAAGGAACGGAATTGGGCTTTGAGAGTGAG-TGCCCCATTAAGAATTCAGCCA

PfFAD8b mRNA    (486) ---AATCAACGGTTTAATTCTTCATTCTGTTCATCAAGTGGGATTAATAAGGAACGGAATTGGGCTTTGAGAGTGAG-TGCCCCATTAAGAATTCAGCCA

PfrFAD7-2 mRNA  (112) ---AATCAACGGTTTAATTCTTCATTCTGTTCATCAAGTGGGATTAATAAGGAACGGAATTGGGCTTTGAGAGTGAG-TGCCCCATTAAGAATTCAGCCA

PfFAD3b mRNA    (122) TACGAAGGAATCG-------GAAAACGT--------------GCGGCCGACAAATTCGACCCCGCCGCGCCGCCGCCGTTCAAGATCGCCGACATCCGAG

PfrFAD3-1 mRNA  (228) TACGAAGGAATCG-------GAAAACGT--------------GCGGCCGACAAATTCGACCCCGCCGCGCCGCCGCCGTTCAAGATCGCCGACATCCGAG

PfFAD3a mRNA    (122) TACGAAGGAATCG-------GAAAGCGT--------------GCGGCCGACAAATTCGACCCCGCCGCGCCGCCGCCCTTCAAGATCGCCGACATCCGAG

PfrFAD3-2 mRNA   (73) TACGAAGGAATCG-------GAAAGCGT--------------GCGGCCGACAAATTCGACCCCGCCGCGCCGCCGCCCTTCAAGATCGCCGACATCCGAG

PfFAD7a mRNA    (516) GTGGAGGAAGAGAGTGAAGAGAGAGGGAGTGTAATAGTAAATGGTGTTGATGAATTCGACCCTGGTGCACCACCACCATTCAAGCTGTCTGATATTCGGG

PfFAD7b mRNA    (516) GTGGAGGAAGAGAGTGAAGAGAGAGGGAGTGTAATAGTAAATGGTGTTGATGAATTCGACCCTGGTGCTCCACCACCATTCAAGCTGTCTGATATTCGGG

PfrFAD7-1 mRNA  (429) GTGGAGGAAGAGAGTGAAGAGAGAGGGAGTGTAATAGTAAATGGTGTTGATGAATTCGACCCTGGTGCACCACCACCATTCAAGCTGTCTGATATTCGGG

PfFAD8a mRNA    (588) GTGGAAGAAGAGA------ACAGAGCGA---------TAAACGGCGGCGAAGAATTCGACCCGGCGGCGCCGCCTCCGTTTAAGTTGTCCGATATAAAGG

PfFAD8b mRNA    (582) GTGGAAGAAGAGA------ACAGAGCGA---------TAAACGGCGGCGAAGAATTCGACCCGGCGGCGCCGCCTCCGTTTAAGTTGTCCGATATTAAGG

PfrFAD7-2 mRNA  (208) GTGGAAGAAGAGA------ACAGAGCGA---------TAAACGGCGGCGAAGAATTCGACCCGGCGGCGCCGCCTCCGTTTAAGTTGTCCGATATTAAGG

PfFAD3b mRNA    (201) CGGCCATACCGGCGCATTGCTGGGTGAAGAATCCGTGGCGCTCATTGAGCTACGTCGTTTGGGACGTCGCCGCCGTCTTCGCGCTGCTCGCCGCCGCCGT

PfrFAD3-1 mRNA  (307) CGGCCATACCGGCGCATTGCTGGGTGAAGAATCCGTGGCGCTCATTGAGCTACGTCGTTTGGGACGTCGCCGCCGTCTTCGCGCTGCTCGCCGCCGCCGT

PfFAD3a mRNA    (201) CGGCCATACCGGCGCATTGCTGGGTGAAGAGTCCGTGGCGCTCATTGAGCTACGTCGTTTGGGACGTCGCCGCCGTATTCGCGCTGCTCGCCGCCGCCGT

PfrFAD3-2 mRNA  (152) CGGCCATACCGGCGCATTGCTGGGTGAAGAGTCCGTGGCGCTCATTGAGCTACGTCGTTTGGGACGTCGCCGCCGTATTCGCGCTGCTCGCCGCCGCCGT

PfFAD7a mRNA    (616) CAGCCATTCCTAAACATTGTTGGGTTAAGGATCCATGGAGGTCTATGAGCTATGTTGTAAGAGATGTTGTTGTTGTTTTTGGATTGGCTGCCGCTGCAGC

PfFAD7b mRNA    (616) CAGCCATTCCTAAACATTGTTGGGTTAAGGATCCATGGAGGTCTATGAGCTATGTTGTAAGAGATGTTGTTGTTGTTTTTGGATTGGCTGCCGCTGCAGC

PfrFAD7-1 mRNA  (529) CAGCCATTCCTAAACATTGTTGGGTTAAGGATCCATGGAGGTCTATGAGCTATGTTGTAAGAGATGTTGTTGTTGTTTTTGGATTGGCTGCCGCTGCAGC

PfFAD8a mRNA    (673) CAGCCATTCCGAAGCATTGTTGGGTGAAGGACCCATGGAGGTCTGTGAGCTATGTGGTGAGGGATGTGGTGGCGGTTTTTGGGATGGCGGCGGCGGCGGC

PfFAD8b mRNA    (667) CAGCCATTCCGAAGCATTGTTGGGTGAAGGACCCATGGAGGTCTGTGAGCTATGTGGTGAGGGATGTGGTGGCGGTTTTTGGGATGGCGGCGGCGGCGGC

PfrFAD7-2 mRNA  (293) CAGCCATTCCGAAGCATTGTTGGGTGAAGGACCCATGGAGGTCTGTGAGCTATGTGGTGAGGGATGTGGTGGCGGTTTTTGGGATGGCGGCGGCGGCGGC

PfFAD3b mRNA    (301) TTACATCAACAGCTGGGCGTTTTGGCCGGTTTACTGGATTGCTCAGGGAACCATGTTTTGGGCCCTTTTCGTTCTTGGGCATGATTGTGGGCACGGGAGT

PfrFAD3-1 mRNA  (407) TTACATCAACAGCTGGGCGTTTTGGCCGGTTTACTGGATTGCTCAGGGAACCATGTTTTGGGCCCTTTTCGTTCTTGGGCATGATTGTGGGCACGGGAGT

PfFAD3a mRNA    (301) TTACATCAACAGCTGGGCGTTTTGGCCGGTTTACTGGATTGCTCAGGGCACCATGTTTTGGGCCCTTTTCGTTCTCGGGCATGATTGTGGGCACGGGAGT

PfrFAD3-2 mRNA  (252) TTACATCAACAGCTGGGCGTTTTGGCCGGTTTACTGGATTGCTCAGGGCACCATGTTTTGGGCCCTTTTCGTTCTCGGGCATGATTGTGGGCACGGGAGT

PfFAD7a mRNA    (716) CTATTTCAACAACTGGGCTGTTTGGCCTATCTACTGGTTCGCTCAGTCAACTATGTTTTGGGCTCTGTTTGTTCTTGGACATGATTGTGGACATGGGAGC

PfFAD7b mRNA    (716) CTATTTCAACAACTGGGCTGTTTGGCCTATCTACTGGTTCGCTCAGTCAACTATGTTTTGGGCTCTGTTTGTTCTTGGACATGATTGTGGACATGGGAGC

PfrFAD7-1 mRNA  (629) CTATTTCAACAACTGGGCTGTTTGGCCTATCTACTGGTTCGCTCAGTCAACTATGTTTTGGGCTCTGTTTGTTCTTGGACATGATTGTGGACATGGGAGC

PfFAD8a mRNA    (773) CTATTTCAACAATTGGCTTGTTTGGCCTTTGTATTGGTTTGCTCAGAGCACCTTATTCTGGGCTCTCTTTGTTCTTGGCCATGACTGTGGTCATGGAAGC

PfFAD8b mRNA    (767) CTATTTCAATAATTGGCTTGTTTGGCCTTTGTATTGGTTTGCTCAGAGCACCTTATTCTGGGCTCTCTTTGTTCTTGGGCATGATTG-------------

PfrFAD7-2 mRNA  (393) CTATTTCAATAATTGGCTTGTTTGGCCTTTGTATTGGTTTGCTCAGAGCACCTTATTCTGGGCTCTCTTTGTTCTTGGCCATGACTGTGGTCATGGAAGC

PfFAD3b mRNA    (401) TTTTCGGACAATACCACGCTGAATAACGTGGTGGGACATGTGCTTCACTCATCAATTCTTGTACCGTATCATGGATGGCGAATCAGCCATAGAACTCACC

PfrFAD3-1 mRNA  (507) TTTTCGGACAATACCACGCTGAATAACGTGGTGGGACATGTGCTTCACTCATCAATTCTTGTACCGTATCATGGATGGCGAATCAGCCATAGAACTCACC

PfFAD3a mRNA    (401) TTTTCAGACAATACCACGCTGAATAACGTGGTGGGACATGTGCTTCACTCATCAATTCTTGTACCGTATCATGGATGGCGAATCAGCCATAGAACTCACC

PfrFAD3-2 mRNA  (352) TTTTCAGACAATACCACGCTGAATAACGTGGTGGGACATGTGCTTCACTCATCAATTCTTGTACCGTATCATGGATGGCGAATCAGCCATAGAACTCACC

PfFAD7a mRNA    (816) TTTTCTAATGATCCCAAGTTGAATAGTGTTGCTGGTCACCTGCTTCACTCTTCCATTCTTGTTCCTTATCATGGATGGAGAATTAGTCACAGGACACACC

PfFAD7b mRNA    (816) TTTTCTAATGATCCCAAGTTGAATAGTGTTGCTGGTCACCTGCTTCACTCTTCCATTCTTGTTCCTTATCATGGATGGAGAATTAGTCACAGGACACACC

PfrFAD7-1 mRNA  (729) TTTTCTAATGATCCCAAGTTGAATAGTGTTGCTGGTCACCTGCTTCACTCTTCCATTCTTGTTCCTTATCATGGATGGAGAATTAGTCACAGGACACACC

PfFAD8a mRNA    (873) TTTTCAAACAACCCCAAGCTGAATAGTGTGTTTGGCCATCTTCTTCACTCTTCAATTCTGGTGCCCTACCATGGATGGAGAATTAGCCATAGAACTCATC

PfFAD8b mRNA    (854) ----------------------------------------------------------------------------------------------------

PfrFAD7-2 mRNA  (493) TTTTCAAACAATCCCAAGCTGAATAGTGTGTTTGGCCATCTTCTTCACTCTTCAATTCTGGTGCCCTACCATGGATGGAGAATTAGCCATAGAACTCATC

PfFAD3b mRNA    (501) ACCAAAACCATGGACATGTCGAGAAGGACGAGTCGTGGGTGCCGTTGCCTGAGAATTTGTACAAGAAGTTGGATTTTTCCACCAAATTCTTGAGATACAA

PfrFAD3-1 mRNA  (607) ACCAAAACCATGGACATGTCGAGAAGGACGAGTCGTGGGTGCCGTTGCCTGAGAATTTGTACAAGAAGTTGGATTTTTCCACCAAATTCTTGAGATACAA

PfFAD3a mRNA    (501) ACCAAAACCATGGACATGTCGAGAAGGACGAGTCGTGGGTGCCGTTGCCTGAGAATTTGTACAAGAAGTTGGATTTTTCCACCAAATTCTTGAGATACAA

PfrFAD3-2 mRNA  (452) ACCAAAACCATGGACATGTCGAGAAGGACGAGTCGTGGGTGCCGTTGCCTGAGAATTTGTACAAGAAGTTGGATTTTTCCACCAAATTCTTGAGATACAA

PfFAD7a mRNA    (916) ATCAGAACCATGGACATGTTGAAAATGATGAATCATGGCATCCAATACCTGAGAAGATATACAGGACTTTGGATTTTGCCACCAAGAAGTTGAGGTTCAC

PfFAD7b mRNA    (916) ATCAGAACCATGGACATGTTGAAAATGATGAATCATGGCATCCAATACCTGAGAAGATATACAGGACTTTGGATTTTGCCACCAAGAAGTTGAGGTTCAC

PfrFAD7-1 mRNA  (829) ATCAGAACCATGGACATGTTGAAAATGATGAATCATGGCATCCAATACCTGAGAAGATATACAGGACTTTGGATTTTGCCACCAAGAAGTTGAGGTTCAC

PfFAD8a mRNA    (973) ATCAGAACCATGGACATGTTGAGAATGATGAATCTTGGCACCCGTTACCTGAGAAGATTTACAATAGCTTGGATAATAATACCAAGATGTTGAGGTTCAC

PfFAD8b mRNA    (854) ----------------------------------------------------------------------------------------------------

PfrFAD7-2 mRNA  (593) ATCAGAACCATGGACATGTTGAGAATGATGAATCTTGGCACCCGTTACCTGAGAAGATTTACAATAGCTTGGATAATAATACCAAGATGTTGAGGTTCAC

PfFAD3b mRNA    (601) AATCCCATTCCCCATGTTTGCATACCCTTTATATTTGTGGTATAGAAGTCCGGGAAAAACTGGATCTCACTTCAACCCTTACAGCGATTTGTTTAAACCA

PfrFAD3-1 mRNA  (707) AATCCCATTCCCCATGTTTGCATACCCTTTATATTTGTGGTATAGAAGTCCGGGAAAAACTGGATCTCACTTCAACCCTTACAGCGATTTGTTTAAACCA

PfFAD3a mRNA    (601) AATCCCATTCCCCATGTTTGCATATCCTTTATATTTGTGGTATAGAAGTCCGGGAAAAACTGGATCTCACTTCAACCCTTACAGCGATTTGTTTAAACCA

PfrFAD3-2 mRNA  (552) AATCCCATTCCCCATGTTTGCATATCCTTTATATTTGTGGTATAGAAGTCCGGGAAAAACTGGATCTCACTTCAACCCTTACAGCGATTTGTTTAAACCA

PfFAD7a mRNA   (1016) TTTGCCTTTCCCCATGCTGGCTTATCCCTTCTATCTGTGGGGAAGAAGTCCTGGCAAGAAAGGCTCTCATTTCCATCCAGACAGTGATTTGTTCGTTCCA

PfFAD7b mRNA   (1016) TTTGCCTTTCCCCATGCTGGCTTATCCCTTCTATCTGTGGGGAAGAAGTCCTGGCAAGAAAGGCTCTCATTTCCATCCAGACAGTGATTTGTTCGTTCCA

PfrFAD7-1 mRNA  (929) TTTGCCTTTCCCCATGCTGGCTTATCCCTTCTATCTGTGGGGAAGAAGTCCTGGCAAGAAAGGCTCTCATTTCCATCCAGACAGTGATTTGTTCGTTCCA

PfFAD8a mRNA   (1073) ATTGCCTTTCCCTATGTTGGCATACCCCTTTTATCTGTGGAGTAGAAGTCCCGGGAAGAAAGGCTCTCATTTCCACCCAGAGAGTGATTTGTTTGTGCCA

PfFAD8b mRNA    (854) ----------------------------------------------------------------------------------------------------

PfrFAD7-2 mRNA  (693) ATTGCCTTTCCCTATGTTGGCATACCCCTTTTATCTGTGGAGTAGAAGTCCCGGGAAGAAAGGCTCTCATTTCCACCCAGAGAGTGATTTGTTTGTGCCA

PfFAD3b mRNA    (701) AATGAGAGGGGTTTGATAGTGACTTCAACAATGTGCTGGGCTGCAATGGGTGTTTTCCTCCTCTATGCCTCCACCATTGTTGGTCCAAACATGATGTTCA

PfrFAD3-1 mRNA  (807) AATGAGAGGGGTTTGATAGTGACTTCAACAATGTGCTGGGCTGCAATGGGTGTTTTCCTCCTCTATGCCTCCACCATTGTTGGTCCAAACATGATGTTCA

PfFAD3a mRNA    (701) AATGAGAGGGGCTTGATAGTGACTTCAACAATGTGCTGGGCTGCAATGGGTGTTTTCCTCCTCTATGCCTCCACCATTGTTGGTCCAAACATGATGTTCA

PfrFAD3-2 mRNA  (652) AATGAGAGGGGCTTGATAGTGACTTCAACAATGTGCTGGGCTGCAATGGGTGTTTTCCTCCTCTATGCCTCCACCATTGTTGGTCCAAACATGATGTTCA

PfFAD7a mRNA   (1116) AACGAGAGGAAAGATGTTATCACCTCAACTGTTTGTTGGACAGCAATGGTTGCAATACTTGCAGGACTATCTTTTGTTATGGGTCCTGTTCAGTTGCTTA

PfFAD7b mRNA   (1116) AACGAGAGGAAAGATGTTATCACCTCAACTGTTTGTTGGACAGCAATGGTTGCAATACTTGCAGGACTATCTTTTGTTATGGGTCCTGTTCAGTTGCTTA

PfrFAD7-1 mRNA (1029) AACGAGAGGAAAGATGTTATCACCTCAACTGTTTGTTGGACAGCAATGGTTGCAATACTTGCAGGACTATCTTTTGTTATGGGTCCTGTTCAGTTGCTTA

PfFAD8a mRNA   (1173) AATGAGAGGAAAGACGTTATTACCTCAACAGTTTGTTGGACTGCAATGGCTGCATTGCTCGTAGGACTATCTTTTGTTATCGGTCCACTCCAGCTGCTCA

PfFAD8b mRNA    (854) ----------------------------------------------------------------------------------------------------

PfrFAD7-2 mRNA  (793) AATGAGAGGAAAGATGTTATTACCTCAACAGTTTGTTGGACTGCAATGGCTGCATTGCTTGTAGGACTATCTTTTGTTATCGGTCCACTCCAGCTGCTCA

PfFAD3b mRNA    (801) AGCTCTACGGCGTACCGTATTTGATATTCGTGATGTGGTTGGACACGGTAACATACTTACACCACCACGGTTATGACAAGAAACTCCCTTGGTACCGCAG

PfrFAD3-1 mRNA  (907) AGCTCTACGGCGTACCGTATTTGATATTCGTGATGTGGTTGGACACGGTAACATACTTACACCACCACGGTTATGACAAGAAACTCCCTTGGTACCGCAG

PfFAD3a mRNA    (801) AGCTCTACGGCGTACCATATTTGATATTCGTGATGTGGTTGGACACGGTAACATACTTACACCACCACGGTTATGACAAGAAACTCCCTTGGTACCGCAG

PfrFAD3-2 mRNA  (752) AGCTCTACGGCGTACCATATTTGATATTCGTGATGTGGTTGGACACGGTAACATACTTACACCACCACGGTTATGACAAGAAACTCCCTTGGTACCGCAG

PfFAD7a mRNA   (1216) AACTCTATGGCATACCTTATATTGGATTTGTGGCATGGCTTGATTTAGTTACCTACTTACACCACCATGGCCACGATGAGAAGCTTCCTTGGTACCGAGG

PfFAD7b mRNA   (1216) AACTCTATGGCATACCTTATATTGGATTTGTGGCATGGCTTGATTTAGTTACCTACTTACACCACCATGGCCACGATGAGAAGCTTCCTTGGTACCGAGG

PfrFAD7-1 mRNA (1129) AACTCTATGGCATACCTTATATTGGATTTGTGGCATGGCTTGATTTAGTTACCTACTTACACCACCATGGCCACGATGAGAAGCTTCCTTGGTACCGAGG

PfFAD8a mRNA   (1273) AACTATACGGCGTTCCTTACTTGGGATTCGTAGCGTGGCTTGATCTTGTGACCTATTTGCATCACCACGGGCATGAAGATAAGCTCCCTTGGTACCGTGG

PfFAD8b mRNA    (854) ----------------------------------------------------------------------------------------------------

PfrFAD7-2 mRNA  (893) AACTATACGGCATTCCTTACTTGGGATTCGTAGCGTGGCTTGATCTCGTGACCTATTTGCATCACCATGGGCATGAAGATAAGCTCCCTTGGTACCGTGG

PfFAD3b mRNA    (901) CAAGGAATGGAGTTATTTACGAGGAGGATTGACGACCGTAGATCAAGATTATGGATTTTTTAATAAAATTCACCACGATATTGGCACCCATGTTATACAC

PfrFAD3-1 mRNA (1007) CAAGGAATGGAGTTATTTACGAGGAGGATTGACGACCGTAGATCAAGATTATGGATTTTTTAATAAAATTCACCACGATATTGGCACCCATGTTATACAC

PfFAD3a mRNA    (901) CAAGGAATGGAGTTATTTACGAGGAGGATTGACGACCGTAGATCAAGATTATGGATTTTTTAATAAAATTCACCACGATATTGGCACCCATGTTATACAC

PfrFAD3-2 mRNA   852) CAAGGAATGGAGTTATTTACGAGGAGGATTGACGACCGTAGATCAAGATTATGGATTTTTTAATAAAATTCACCACGATATTGGCACCCATGTTATACAC

PfFAD7a mRNA   (1316) AAAGGAATGGAGTTACCTGAGAGGGGGGCTCACGACACTTGATCGCGATTATGGATGGATAAACAACATCCACCATGACATAGGGACGCATGTTATACAT

PfFAD7b mRNA   (1316) AAAGGAATGGAGTTACCTGAGAGGGGGGCTCACGACACTTGATCGCGATTATGGATGGATAAACAACATCCACCATGACATAGGGACGCATGTTATACAT

PfrFAD7-1 mRNA (1229) AAAGGAATGGAGTTACCTGAGAGGGGGGCTCACGACACTTGATCGCGATTATGGATGGATAAACAACATCCACCATGACATAGGGACGCATGTTATACAT

PfFAD8a mRNA   (1373) AAAGGAATGGAGTTATCTGAGAGGGGGGCTCACGACACTTGATCGTGACTACGGATTGATCAACAACATCCACCATGACATAGGAACTCATGTCATACAC

PfFAD8b mRNA    (854) ----------------------------------------------------------------------------------------------------

PfrFAD7-2 mRNA  (993) AAAGGAATGGAGTTATCTGAGAGGGGGGCTCACGACACTTGACCGCGACTACGGATTGATCAACAACATCCACCACGACATAGGAACTCATGTCATACAC

PfFAD3b mRNA   (1001) CATCTATTCCCTCAGATCCCACATTACCACTTAGTGGAGGCGACAAGGGAGGCGAAAAGGGTGCTGGGGAATTACTACAGGGAGCCCAGAAAATCTGGGC

PfrFAD3-1 mRNA (1107) CATCTATTCCCTCAGATCCCACATTACCACTTAGTGGAGGCGACAAGGGAGGCGAAAAGGGTGCTGGGGAATTACTACAGGGAGCCCAGAAAATCTGGGC

PfFAD3a mRNA   (1001) CATCTATTCCCTCAGATCCCACATTACCACTTAGTGGAGGCGACAAGGGAGGCGAAAAGGGTGCTGGGGAATTACTACAGGGAGCCCAGAAAATCTGGGC

PfrFAD3-2 mRNA  (952) CATCTATTCCCTCAGATCCCACATTACCACTTAGTGGAGGCGACAAGGGAGGCGAAAAGGGTGCTGGGGAATTACTACAGGGAGCCCAGAAAATCTGGGC

PfFAD7a mRNA   (1416) CACCTCTTCCCACAAATACCACACTACCATTTGATAGAAGCAACTGCAGCAGCTAAGCCAGTTCTAGGAAAATATTACAAGGAGCCTAAGAAATCAGGCC

PfFAD7b mRNA   (1416) CACCTCTTCCCACAAATACCACACTACCATTTGATAGAAGCAACTGCAGCAGCTAAGCCAGTTCTAGGAAAATATTACAAGGAGCCTAAGAAATCAGGCC

PfrFAD7-1 mRNA (1329) CACCTCTTCCCACAAATACCACACTACCATTTGATAGAAGCAACTGCAGCAGCTAAGCCAGTTCTAGGAAAATATTACAAGGAGCCTAAGAAATCAGGCC

PfFAD8a mRNA   (1473) CACCTCTTCCCCCAAATCCCACACTACCATTTGATAGAAGCTACTGAAGCAGCTAAGGGGGTATTAGGCAAGTACTACAGGGAGCCGAAAAAGTCGGGCC

PfFAD8b mRNA    (854) ----------------------------------------------------------------------------------------------------

PfrFAD7-2 mRNA (1093) CACCTCTTCCCCCAAATTCCACACTACCATTTGATAGAAGCTACTGAAGCAGCTAAGGGGGTATTAGGCAAGTACTACAGGGAGCCGAAAAAGTCGGGCC

PfFAD3b mRNA   (1101) CAGTTCCACTACACTTAATTCCTGCCTTGTTGAAAAGTCTTGGTAGAGATCATTATGTTAGTGATAATGGAGACATAGTTTATTATCAAACAGATGATGA

PfrFAD3-1 mRNA (1207) CAGTTCCACTACACTTAATTCCTGCCTTGTTGAAAAGTCTTGGTAGAGATCATTATGTTAGTGATAATGGAGACATAGTTTATTATCAAACAGATGATGA

PfFAD3a mRNA   (1101) CAGTTCCACTGCACTTAATTCCTGCCTTGTTGAAAAGTCTTGGTAGAGATCATTATGTTAGTGATAATGGAGACATAGTTTATTATCAAACAGATGATGA

PfrFAD3-2 mRNA (1052) CAGTTCCACTGCACTTAATTCCTGCCTTGTTGAAAAGTCTTGGTAGAGATCATTATGTTAGTGATAATGGAGACATAGTTTATTATCAAACAGATGATGA

PfFAD7a mRNA   (1516) CCTTTCCATTCTACTTGTTGGGAGTCCTCCAAAAAAGCATGAAAAAGGATCACTATGTGAGTGACACGGGCGATATCGTTTACTACCAGACCGATCCTGA

PfFAD7b mRNA   (1516) CCTTTCCATTCTACTTGTTGGGAGTCCTCCAAAAAAGCATGAAAAAGGATCACTATGTGAGTGACACGGGCGATATCGTTTACTACCAGACCGATCCTGA

PfrFAD7-1 mRNA (1429) CCTTTCCATTCTACTTGTTGGGAGTCCTCCAAAAAAGCATGAAAAAGGATCACTATGTGAGTGACACGGGCGATATCGTTTACTACCAGACCGATCCTGA

PfFAD8a mRNA   (1573) CTCTACCGTTACACTTGTTGGGAGACCTCCTGAGAAGCATGAAGAAGGATCACTACGTGAGCGACACCGGCGACATTGTCTATTATCAGACAGATCCTCA

PfFAD8b mRNA    (854) ----------------------------------------------------------------------------------------------------

PfrFAD7-2 mRNA (1193) CTCTACCGTTACACTTGTTGGGAGAACTCCTGAGAAGCATGAAGAAGGATCACTATGTGAGCGACACCGGCGACATTGTCTATTATCAGACAGATCCTCA

PfFAD3b mRNA   (1201) GCTCTTTCCTTC-CAAAAAGATTTAGTGATGGACTCTTGATTGCCAAATTAGAT-----TTAATTTACAGTAGTCCTTTGTGCCACAATATTTTGTTTAG

PfrFAD3-1 mRNA (1307) GCTCTTTCCTTC-CAAAAAGATTTAGTGATGGACTCTTGATTGCCAAATTAGAT-----TTAATTTACAGTAGTCCTTTGTGCCACAATATTTTGTTTAG

PfFAD3a mRNA   (1201) GCTCTTTCCTTC-CAAAAAGATTTAGTGATGGACTCTTGATTGCCAAATTAGAT-----TTAATTTACAGTAGTCCTTTGTGCCACAATATTTTGTTTAG

PfrFAD3-2 mRNA (1152) GCTCTTTCCTTCCAAAAAGATTTAG---------------------------------------------------------------------------

PfFAD7a mRNA   (1616) GCTGAATTGAACTCAAGAATGATAAGAGTTTGAATGTTGTTATCAGTATATGTAAAAGCTGTCTCTAATTGAGTTCTCGAGACCTCTAAGGATCGCGTTC

PfFAD7b mRNA   (1616) GCTGAATTGAACTCAAGAATGATAAGAGTTTGAATGTTGTTATCAGTATATGTAAAAGCTGTCTCTAATTGAGTCCTCGAGACCTCTAAGGATCGCGTTC

PfrFAD7-1 mRNA (1529) GCTGAATTGAACTCAAGAATGATAAGAGTTTGAATGTTGTTATCAGTATATGTAAAAGCTGTCTCTAATTGAGTTCTCGAGACCTCTAAGGATCGCGTTC

PfFAD8a mRNA   (1673) GCTCAATGGAGGTCGCAAATCTTAGGCTGTAGTGAAAGAATTTATCGATTCTTTGTCAGCTGAGTCTAATTATTAGTGGCTGTTAAGGATAGTGTATGAG

PfFAD8b mRNA    (854) ----------------------------------------------------------------------------------------------------

PfrFAD7-2 mRNA (1293) GCTCAATGGAGGTCGCAAATCTTAG---------------------------------------------------------------------------

PfFAD3b mRNA   (1295) GCCAGGAAATATTGTGTGCACAAATTAAATAACTCTAGTGAGTT---TTTTTTGGATCAAGTGTTTGTTACCTTTTTTTTTTTT------CCTGTGATAA

PfrFAD3-1 mRNA (1401) GCCAGGAAATATTGTGTGCACAAATTAAATAACTCTAGTGAGTTT--TTTTTTGGATCAAGTGTTTGTTACCTTTTTTTTTTTTTTTTTTCCTGTGATAA

PfFAD3a mRNA   (1295) GCCAGGAAATATTGTGTGCACAAATTAAATAACTCTAGTGAGTTTTTTTTTTTGGATCAAGTGTTTGTTACCTTTTCTTTTTC--------CTGTGATAA

PfrFAD3-2 mRNA (1177) ----------------------------------------------------------------------------------------------------

PfFAD7a mRNA   (1716) AGCTGCAGAATTGATATATATATTTTTTTCTCTTCAACGATGGAGAGGCGATACTAGAGATTACAGAATATTGATGTGTATTT--------GTATGA-GG

PfFAD7b mRNA   (1716) AGCTGCAGAATTGATATATATATTTTTTTCTCTTCAACGATGGAGAGGCGATACTAGAGATTACAGAATATTGATGTGTATTT--------GTATGA-GG

PfrFAD7-1 mRNA (1629) AGCTGCAGAATTGATATATATATTTTTTTCTCTTCAACGATGGAGAGGCGATACTAGAGATTACAGAATATTGATGTGTATTT--------GTATGA-GG

PfFAD8a mRNA   (1773) CTCATATCATACTACCATGCCAACATAGGTAATTTTTTTCGAATACAATTGATTTTTGCTTCAATCCATTGATGGAGCACCGT--------ATAAAT-AC

PfFAD8b mRNA    (854) ----------------------------------------------------------------------------------------------------

PfrFAD7-2 mRNA (1318) ----------------------------------------------------------------------------------------------------

PfFAD3b mRNA   (1386) ATGTAATGTTCCTTAAATAAACTTATTTTT----------GGTTATTTTGTTGACCTTTGAAAAAAAAAAAAAAAAAAAAAAAAAAAAAA----------

PfrFAD3-1 mRNA (1499) ATGTAATGTTCCTTAAATAAACTTATTTTT----------GGTTATTTTGTTGACC--------------------------------------------

PfFAD3a mRNA   (1387) ATGTAATGTTCCTTAAATAAACTTATTTTTGGTTTTTGTGGGTTATTTTGTTGACCTCTAAAAAAAAAAAAAAAAAAAAAAAAAAAAAAA----------

PfrFAD3-2 mRNA (1177) ----------------------------------------------------------------------------------------------------

PfFAD7a mRNA   (1807) ATGTACTGATGCTATAATGTATCTTGATAATTAATGAGAGGGAAAGCCAGATTATTCTCTAAAAAAAAAAAAAAAAAAAAAAAAAAAAA-----------

PfFAD7b mRNA   (1807) ATGTACTGATGCTATAATGTATCTTGATAATTAATGAGAGGGAAAGCCAGATTATTCTCTAAAAAAAAAAAAAAAAAAAAAAAAAAAAA-----------

PfrFAD7-1 mRNA (1720) ATGTACTGATGCTATAATGTATCTTGATAATTAATGAGAGGGAAAGCCAGATTATTCTCAAAAAAAAAAAAAAAAAAA----------------------

PfFAD8a mRNA   (1864) GTATTTATTGCATTTGTCCTAGAATATGAATGATAAAGTAATGAATTTCCATGTCGAAGGGAAACTCTTTTTCCCCACGGCTTCTTGTTCTTCCAAAAAA

PfFAD8b mRNA    (854) ----------------------------------------------------------------------------------------------------

PfrFAD7-2 mRNA (1318) ----------------------------------------------------------------------------------------------------

PfFAD3b mRNA   (1466) ---------------------

PfrFAD3-1 mRNA (1545) ---------------------

PfFAD3a mRNA   (1477) ---------------------

PfrFAD3-2 mRNA (1177) ---------------------

PfFAD7a mRNA   (1896) ---------------------

PfFAD7b mRNA   (1896) ---------------------

PfrFAD7-1 mRNA (1798) ---------------------

PfFAD8a mRNA   (1964) AAAAAAAAAAAAAAAAAAAAA

PfFAD8b mRNA    (854) ---------------------

PfrFAD7-2 mRNA (1318) ---------------------

**S2 Fig. Multi-alignment of perilla *ω-3 FAD* mRNAs in this study and previous reports.** *PfrFAD3-1* (NCBI accession no AF047039.1), *PfrFAD3-2* (KX228917.1), *PfrFAD7-1* (U59477.1) and *PfrFAD7-2* (KP070824.1).
