# Supplementary material for: Omega-3 fatty acid desaturase gene family from two ω-3 sources, Salvia hispanica and Perilla frutescens: Cloning, characterization and expression
Source: PLoS One. 2018 Jan 19;13(1):e0191432. doi: 10.1371/journal.pone.0191432 (PMC5774782; doi:10.1371/journal.pone.0191432)
Supplement: S3 Fig — They were predicted by TOPCONS (http://topcons.net/) [59], with default parameters. (DOCX) [file pone.0191432.s007.docx]

**ShFAD3-1 ShFAD3-2**


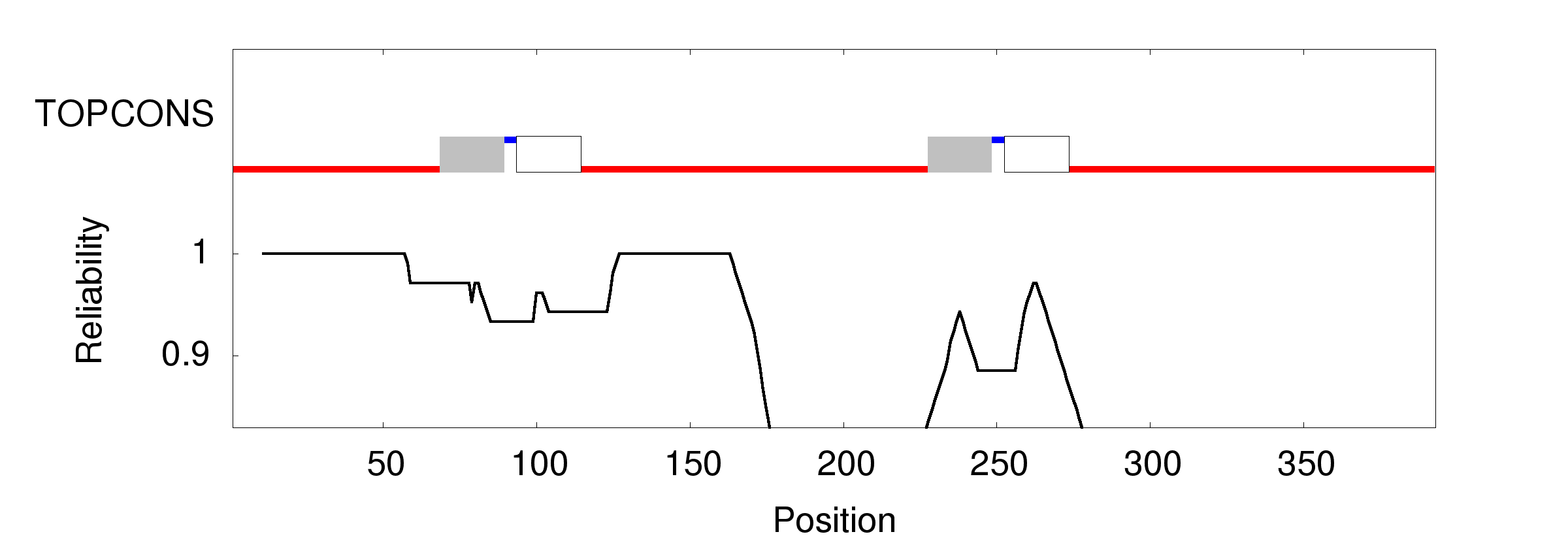

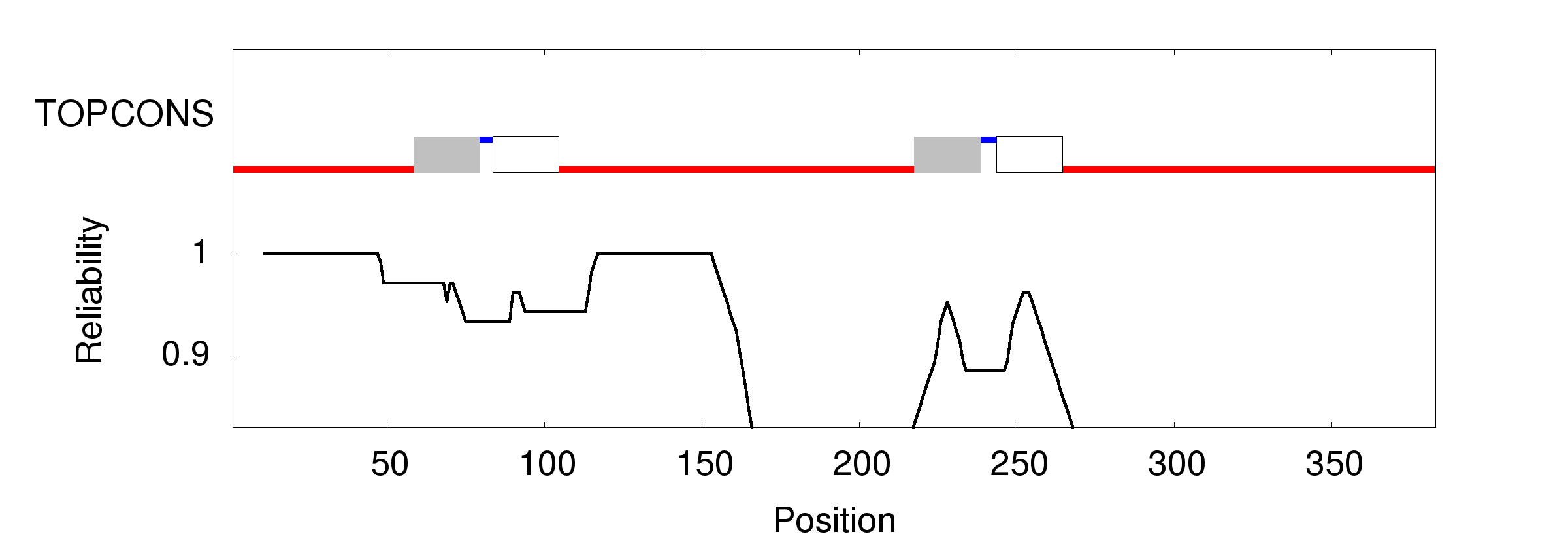


**ShFAD7a/b ShFAD8**


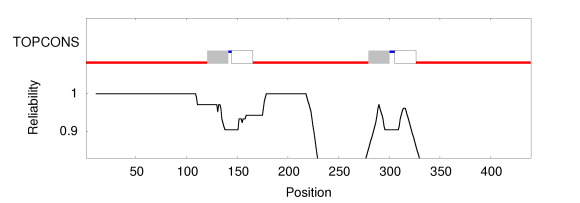

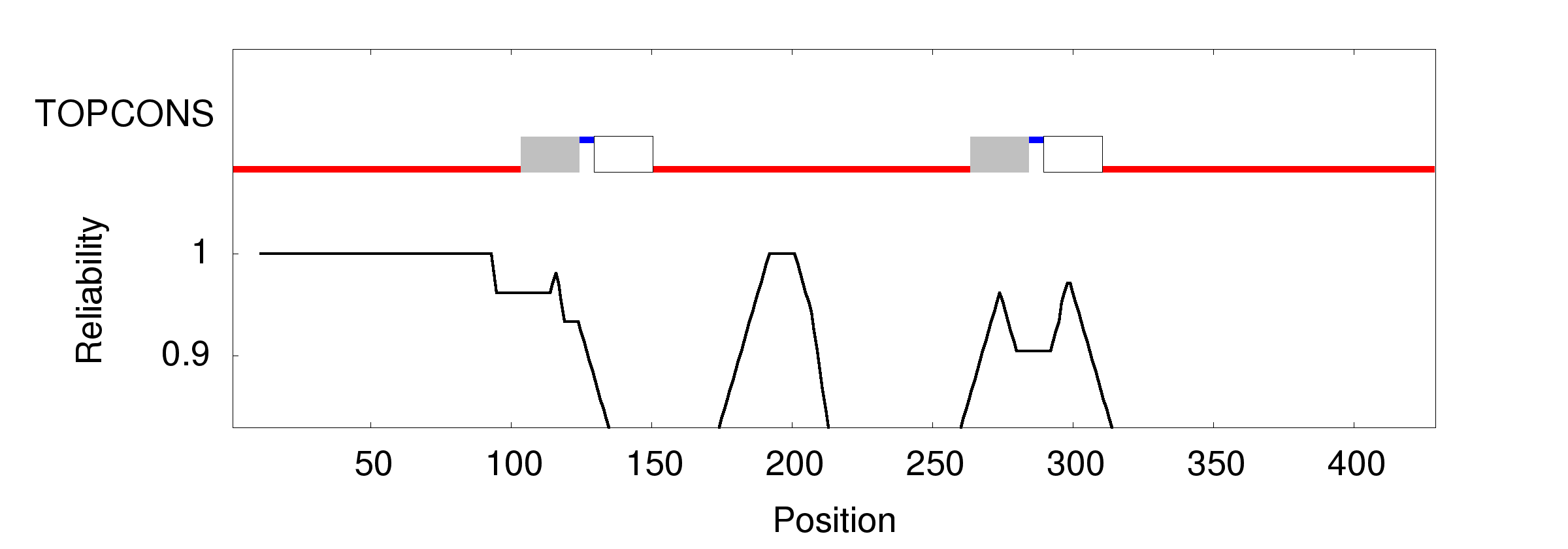


**PfFAD3a PfFAD3b**


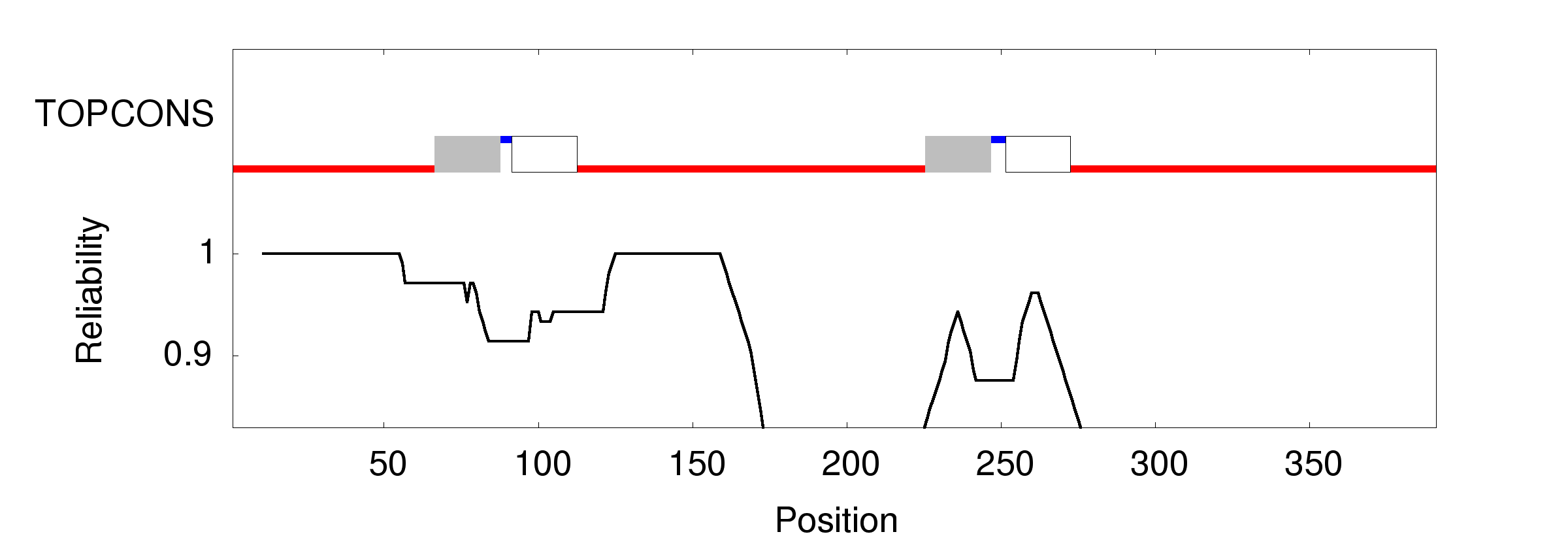

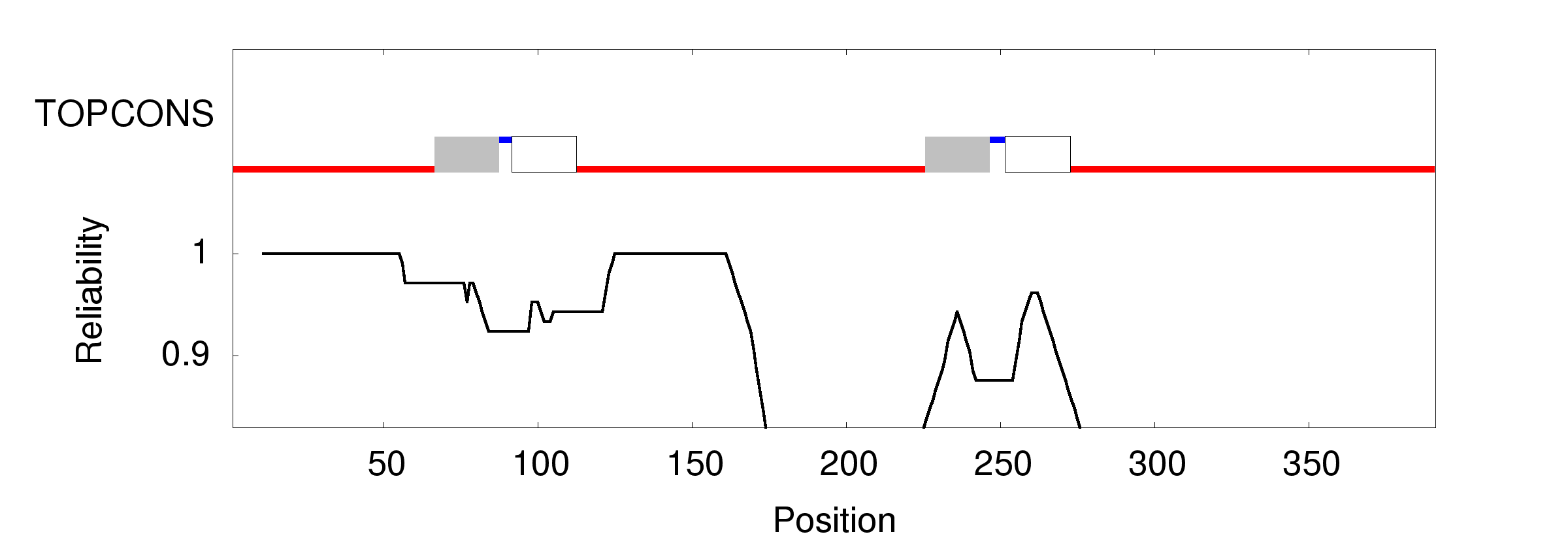


**PfFAD7a/b PfFAD8a**


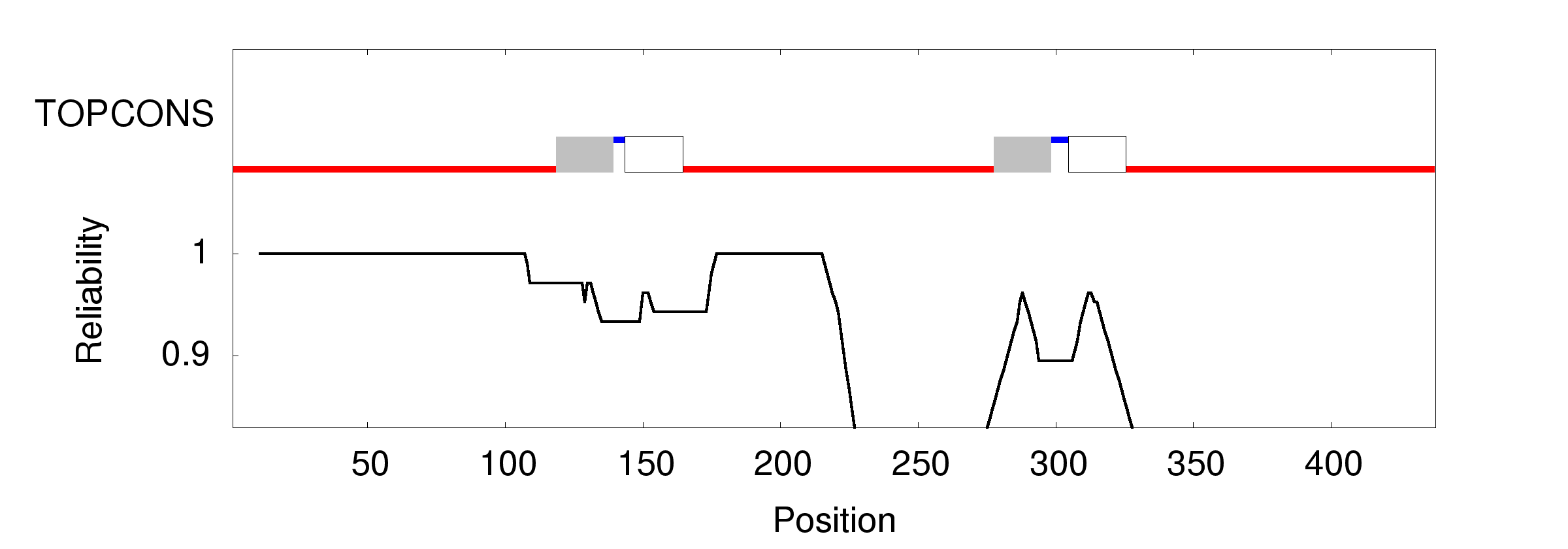

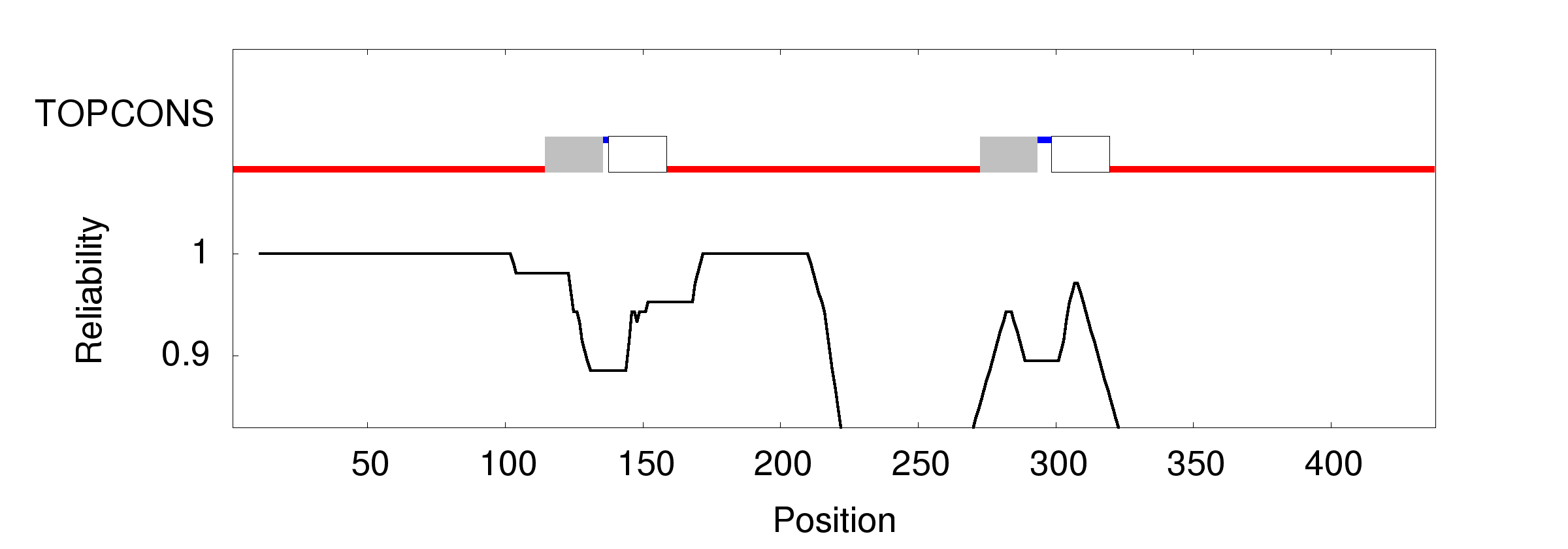


**PfFAD8b**


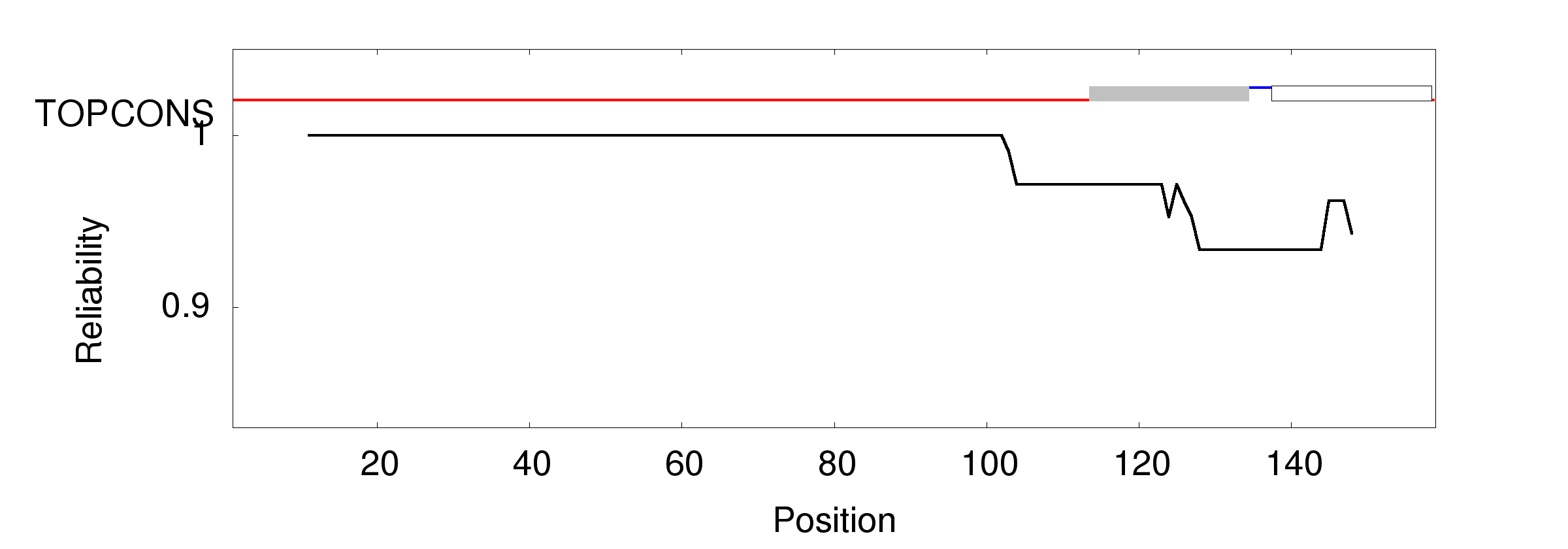

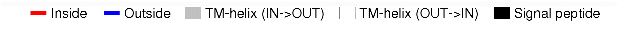


**S3 Fig. Transmebrane helices of ω-3 FAD proteins from chia and perilla.** They were predicted by TOPCONS (<http://topcons.net/>) [59], with default parameters.
