# Supplementary material for: Omega-3 fatty acid desaturase gene family from two ω-3 sources, Salvia hispanica and Perilla frutescens: Cloning, characterization and expression
Source: PLoS One. 2018 Jan 19;13(1):e0191432. doi: 10.1371/journal.pone.0191432 (PMC5774782; doi:10.1371/journal.pone.0191432)
Supplement: S4 Fig — They were predicted by SOPMA [70]. Alfa-helix, extended strand, β-turn and random coils are shown with the longest, middle long, short and the shortest vertical bars, respectively. (DOCX) [file pone.0191432.s008.docx]

**ShFAD3-1**


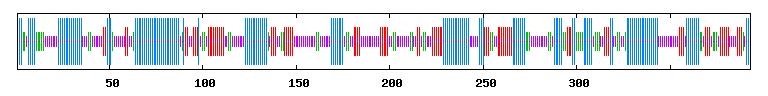


**ShFAD3-2**


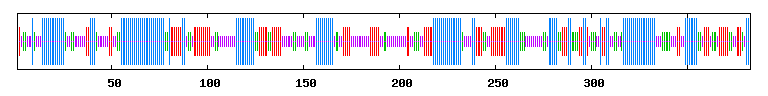


**ShFAD7a/b**


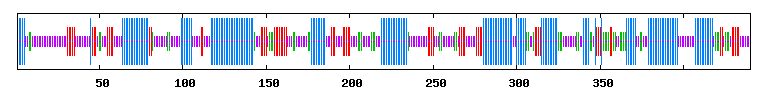


**ShFAD8**


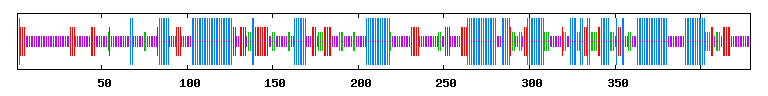


**PfFAD3a**


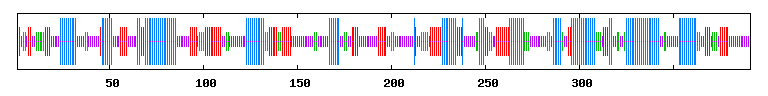


**PfFAD3b**


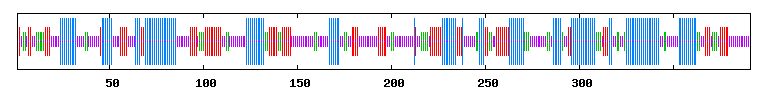


**PfFAD7a/b**


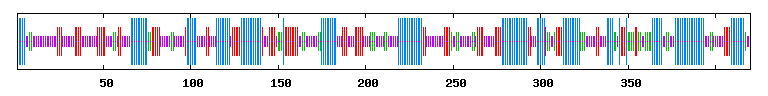


**PfFAD8a**


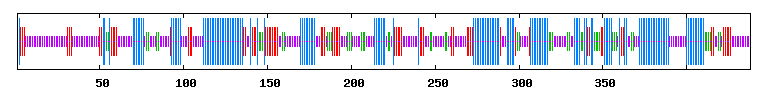


**PfFAD8b**


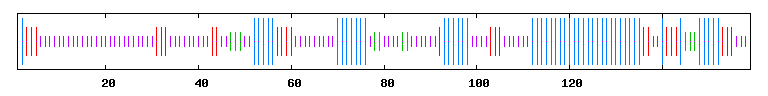


**S4 Fig. Secondary structures ofω-3 FAD proteins from chia and perilla.** They were predicted by SOPMA [70]. Alfa-helix, extended strand, β-turn and random coil are showed with the longest, middle long, short and the shortest vertical bars, respectively.
